# Expression of the primate-specific *LINC00473* RNA in mouse neurons promotes excitability and CREB-regulated transcription

**DOI:** 10.1016/j.jbc.2023.104671

**Published:** 2023-04-03

**Authors:** Priit Pruunsild, C. Peter Bengtson, Isabel Loss, Benjamin Lohrer, Hilmar Bading

**Affiliations:** Department of Neurobiology, Interdisciplinary Center for Neurosciences (IZN), Heidelberg University, Heidelberg, Germany

**Keywords:** gene regulation, transcriptomics, human, brain, molecular evolution, electrophysiology

## Abstract

The *LINC00473* (*Lnc473*) gene has previously been shown to be associated with cancer and psychiatric disorders. Its expression is elevated in several types of tumors and decreased in the brains of patients diagnosed with schizophrenia or major depression. In neurons, *Lnc473* transcription is strongly responsive to synaptic activity, suggesting a role in adaptive, plasticity-related mechanisms. However, the function of *Lnc473* is largely unknown. Here, using a recombinant adeno-associated viral vector, we introduced a primate-specific human *Lnc473* RNA into mouse primary neurons. We show that this resulted in a transcriptomic shift comprising downregulation of epilepsy-associated genes and a rise in cAMP response element-binding protein (CREB) activity, which was driven by augmented CREB-regulated transcription coactivator 1 nuclear localization. Moreover, we demonstrate that ectopic *Lnc473* expression increased neuronal excitability as well as network excitability. These findings suggest that primates may possess a lineage-specific activity-dependent modulator of CREB-regulated neuronal excitability.

*LINC00473* (*Lnc473*) is among the few neuronal activity-regulated genes in human that do not have an ortholog in common experimental model species, such as the mouse (1, 2, 3). Nonetheless, *Lnc473* expression is controlled by evolutionarily conserved synapse-to-nucleus signaling mechanisms and it belongs to the core set of activity-responsive genes in human neurons (1). *Lnc473* is a target of the transcription factors cAMP response element-binding protein (CREB) and neuronal PAS domain protein 4 (NPAS4) (1), which in rodents are key controllers of neuronal plasticity, network excitability, acquired neuroprotection, and memory formation (4, 5, 6, 7, 8). Thus, this lineage-specific addon to the synaptic activity-driven gene program may contribute to plasticity-involving adaptations that are required for brain development and maturation, determine cognitive performance, and could kindle psychiatric disorders when dysregulated (9, 10, 11, 12, 13, 14, 15). Indeed, DNA copy number variations overlapping the *Lnc473* locus are associated with schizophrenia (16) and *Lnc473* expression levels in the brain are decreased in major depressive disorder in females (17). Moreover, *Lnc473* has been highlighted as a hub gene of a neuronal activity-dependent gene network module, which is downregulated in schizophrenia (18). Collectively, these data suggest that a disturbance in expression of *Lnc473* in humans may be linked to heightened risk for psychiatric disorders.

*Lnc473* has been shown to code for a nuclear RNA both in nonneuronal cells and in neurons (17, 19) but its localization is likely not restricted to the nucleus (20, 21). Partial cytoplasmic localization is supported by the findings that *Lnc473* is a target of the microRNA miR-34a (22), which is a psychiatric disease-associated microRNA that in neurons regulates synaptic maturation (23), and functions as a thermogenic regulator in adipocytes, where it localizes to the mitochondrial–lipid droplet interphase (20). *Lnc473* expression is low in general, with the exception of some tissues, such as the ovary, the pituitary gland, and the fallopian tube (gtexportal.org (24)). However, it is strongly inducible by cAMP signaling (20, 25) as well as synaptic activity (1), and, accordingly, is clearly detectable in the brain postnatally (brainspan.org (26, 27)) when environmental stimuli start to affect brain development. *Lnc473* is also elevated in diverse types of cancers (19, 22, 28, 29) and it may be oncogenic *via* various routes (see for example (30, 31)). Yet, a conspicuous function of *Lnc473* in tumor cells appears to be amplification of its own expression by a positive feedback loop that involves CREB-mediated transcription (19). Of note, *Lnc473* has also been proposed to be a negative regulator of CREB-dependent transcription, since its knock-down was observed to positively affect cAMP-responsive gene expression (25). The link to CREB function is significant in the context of psychiatric disorders as disturbances in CREB levels as well as activity are implicated in major depression (32) and schizophrenia (33, 34), which are both associated with downregulated *Lnc473* in the brain (17, 18). Moreover, ectopic expression of *Lnc473* in mouse prefrontal cortex was recently shown to perturb gene regulation, possibly including cAMP/CREB-dependent transcription, selectively in females, where this correlated with rescue from depression- as well as anxiety-related behavior (17).

To investigate the functional impact of *Lnc473* expression on neurons we used a gain-of-function approach in mouse cells that lack this RNA. The activity-regulated *Lnc473* is absent also from the latest human genome assembly (hg38), because the multiple transcripts previously assigned to it (hg19) coalesce into the *PDE10A* gene, which is not activity-responsive *en bloc* (1). We therefore identified the *Lnc473* transcript that is induced by synaptic activity from the human *PDE10A* locus and focused on the effects it has on gene expression and electrophysiological properties in mouse primary hippocampal neurons.

## Results

The *PDE10A* gene locus where *Lnc473* resides contains conserved exons in the downstream segment where most of the coding exons are located but includes several exons that lack a mouse counterpart in the upstream portion (Fig. S1, *A* and *B*). We refer to RNAs produced from this largely nonconserved upstream region as *Lnc473* transcripts. However, in hg38 *Lnc473* is interpreted as a structural constituent of *PDE10A* because two of the 5′ exons are shared between species and can be used in *PDE10A* mRNAs that include the conserved downstream protein-coding exons. The intricate gene structure prompted us to ask what exactly is induced by neuronal activity in this locus?

### Specific Lnc473 exons are upregulated in response to synaptic activity

**Figure 1:**
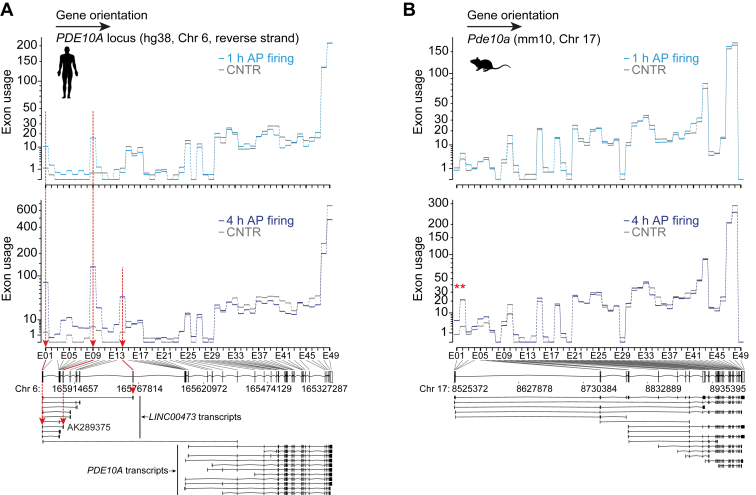
**Human and mouse *PDE10A*/*Pde10a* exon usage in response to synaptic activity.** Samples of human induced pluripotent stem cell (iPSC)-derived neuron and mouse primary hippocampal neuron cocultures (1) were used to determine expression levels of individual human *PDE10A* (*A*) or mouse *Pde10a* (*B*) exons with DEXSeq (103). Exon usage after 1 or 4 h of action potential (AP) firing, stimulated with bicuculline (Bic, 50 μM) and 4-aminopyridine (250 μM), in comparison to the control condition (CNTR) is shown (n = 4, for CNTR and 1 h AP firing; n = 3, for 4 h AP firing). Gene structures and transcript models are shown below exon usage plots. *A*, *red arrows* and *lines* indicate the exons the usage of which was strongly upregulated by AP firing. GenBank accession number AK289375 specifies the *Lnc473* transcript used in this study for ectopic expression in mouse primary neurons. *B*, note that, in the mouse gene annotation the two upstream 5′ exons are each divided into two counting bins (E01 + E02 and E03 + E04). The bins displaying more usage after 4 h of AP firing (E01 and E02) are marked with *red asterisks* and in sum represent the ortholog of human exon E01. *A* and *B*, exon usage measures the level of inclusion of the exon into transcripts relative to inclusion levels of all other exons of the same gene. For overall, per gene expression data see Table S5. Also, DEXSeq counting bin numbers of the human and mouse genes do not represent one-to-one exon orthologs.

To investigate differential activity-dependent exon usage across human and mouse *PDE10A* loci, we reanalyzed an RNA-seq dataset of gene expression changes upon synaptic activity in human and mouse neurons (1). The pattern of exon usage in the human gene locus after 1 h of increased synaptic activity (action potential (AP) firing; Fig. 1*A* and Table S1) is consistent with upregulation of a particular two-exon transcript from the upstream segment of the gene. This upregulation was more apparent at the 4-h time point, where, additionally, increased usage of some other nonconserved human exons was detectable (*e.g.*, E14; Fig. 1*A* and Table S2). In mouse neurons usage of the first 5′ *Pde10a* exon was elevated after 4 h of AP bursting with a concurrent increase in usage of the two nearest internal exons (Fig. 1*B*, Tables S3 and S4). This helps to explain the upregulation of the mouse *Pde10a* gene, which contrasts with human *PDE10A* that was not significantly increased by synaptic activity ((1) and Table S5). The results of this analysis indicated that the RNA species that is rapidly and particularly strongly upregulated in response to synaptic activity specifically from the human *PDE10A* gene locus corresponds to the *Lnc473* transcript variant with the GenBank accession number AK289375 (hereafter the only transcript variant denoted by *Lnc473*).

### Lnc473 RNA is conserved in higher primates

**Figure 2:**
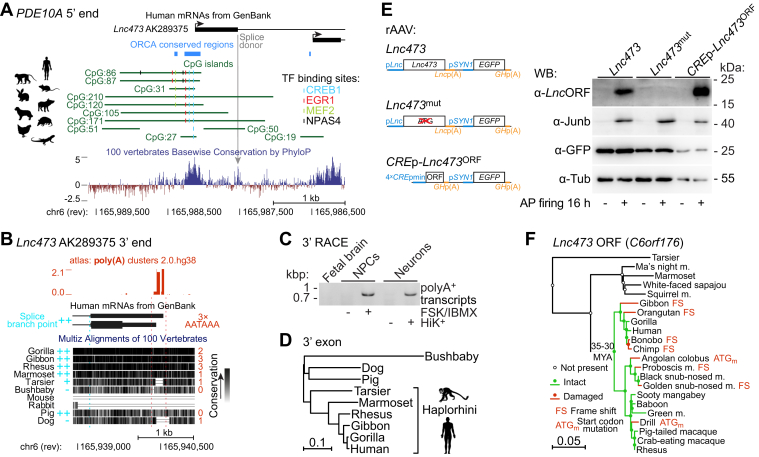
***Lnc473* RNA is conserved in higher primates.***A*, genomic region of the human *PDE10A* 5′ end visualized in the UCSC genome browser. Arrows attached to GenBank mRNAs denote transcription start sites (TSSs). The *Lnc473* AK289375 transcript 5′ exon-coding sequence is preceded by a highly conserved region, which comprises activity-regulated transcription factor (TF) binding sites. The “ORCA conserved regions” track displays sequence blocks containing conserved transcription factor binding sites detected by phylogenetic footprinting with the ORCA toolkit (110). Basewise conservation in vertebrates by PhyloP assigns positive scores to conserved positions (*blue*) and negative scores to fast-evolving sites (*reddish brown*). *B*, genomic region covering the human *Lnc473* AK289375 3′ exon visualized in the UCSC genome browser. “atlas: poly(A) clusters 2.0.hg38” track (35) shows transcription termination sites. The thicker region of a AK289375 3′ exon in the GenBank mRNA track designates the ORF *C6orf176* (see below). Alignment of the genomic region with orthologous sequences in exemplar mammals is also shown. Presence of a splice branch point is indicated by *light blue* (++, strong signal; + weak signal; -, no signal) and presence of polyadenylation (poly(A)) signals is indicated with red (poly(A) count). *C*, 3′ rapid amplification of cDNA ends (3′ RACE) analysis results show termination of transcription at the *Lnc473* AK289375 3′ exon poly(A) cluster in human neuronal cells. NPCs and Neurons, human cultured iPSC-derived neuronal precursor cells and neurons, respectively. Cells were left untreated (−) of were treated (+) for 4 h with forskolin (FSK, 10 μM) and IBMX (500 μM), or a high potassium concentration-containing solution (HiK^+^, 50 mM) to induce *Lnc473* expression. Human fetal brain RNA was used as a control. *D*, phylogenetic tree based on orthologous sequences of the human *Lnc473* 3′ exon. *E*, Western blot analysis showing expression of the *Lnc473* ORF (*Lnc473*^ORF^) in mouse primary neurons after induction of AP firing. rAAV infection was used to deliver the full-length WT human *Lnc473* cDNA or the *Lnc473* cDNA with mutated ORF start codon (*Lnc473*^mut^), both under the control of the native promoter of *Lnc473* (p*Lnc*); or the *Lnc473*^ORF^ cDNA under the control of four CRE elements and a minimal promoter (4 × *CRE*pmin; *CRE*p-*Lnc473*^ORF^) into mouse primary neurons. AP firing was induced with Bic (50 μM). Expression of Junb, EGFP, and Tubulin was used to test for Bic activation, viral infection, and sample loading, respectively. Note that comparatively less of *Lnc473*^ORF^ samples were loaded because of the strong induction response of 4 × *CRE*pmin. *F*, phylogenetic tree of dry-nosed primate *Lnc473* 3′ exon orthologs. Ancestral sequence reconstruction reveals the intact *Lnc473* ORF (*green*) and loss of the ORF (*red*) by frame-shift (FS) or start codon mutations (ATG_m_). CRE, cAMP response element; m, monkey; MYA, million years ago; NPC, neuronal precursor cell.

We sought to describe this human *Lnc473* transcript with respect to molecular evolution. Its activity-responsive 5′ exon is linked to a highly conserved stretch of DNA containing *cis*-regulatory elements for the neuronal activity-regulated transcription factors CREB, early growth response protein 1, myocyte enhancer factor 2, or NPAS4 (Fig. 2*A*). This promoter sequence is more conserved than the exon, although the flanking splice donor is also highly conserved in vertebrates, excluding fish (Fig. 2*A*). Notably, the other 5′ exon that was not induced by AP firing (Fig. 1*A*) is conserved but pertains to a more divergent promoter (Fig. 2*A*). These data suggest that in response to neuronal activity a *Lnc473* transcript-like 5′ exon could be produced and spliced in most vertebrates. Nonetheless, expression of the full-length, spliced 3′ exon-containing RNA cannot be common, because the downstream genomic region is largely distinct in nonmammalian vertebrates and is much less conserved in mammals, being remarkably different in glires, including mice (Figs. 2*B* and S1). However, in simian primates the splicing acceptor and branch point as well as polyadenylation (poly(A)) signals required for transcript maturation are available (Fig. 2*B*). Some of these sites can be found also in other mammalian species, although their presence seems random and a lack of any of them would exclude analogous exon usage (*e.g.*, in pig or dog, Fig. 2*B*). The human *Lnc473* 3′ exon poly(A) sites are functional (Fig. 2*B*, atlas: poly(A) clusters track (35)) and used in neuronal cells (Fig. 2*C*). All these data, together with the phylogenetic tree based on the 3′ exon orthologs in exemplar mammals (Fig. 2*D*) suggest that RNAs related to the neuronal activity-induced *Lnc473* transcript are expressed in dry-nosed primates (suborder Haplorhini) including apes and Old World as well as New World monkeys but excluding Lemuriformes, such as bushbaby.

Sequences identical to human *Lnc473* have been formerly categorized as protein-coding (termed *C6orf176* in hg18) (36, 37). Moreover, a recent ribosome profiling study showed that *Lnc473* RNA is translated in human embryonic stem cell-derived neurons after membrane depolarization (38). We determined by Western blot analysis with a custom-made antibody (ab) that an ORF (UniProt A8K010) of *Lnc473* (*Lnc473*^ORF^) can indeed be translated when the full-length *Lnc473* complementary DNA (cDNA) under the control of its own promoter (p*Lnc*, Fig. 2*E*) is delivered into mouse primary hippocampal neurons with a recombinant adeno-associated virus (rAAV) (Fig. 2*E*, *Lnc473*; *EGFP* under the control of the human *Synapsin I* promoter (p*SYN1*) was included in tandem for monitoring infection). However, the levels of the protein were high enough to be detectable only if AP firing was induced with the GABA type A receptor antagonist bicuculline (Bic) in the cultures, similar to the expression of a control construct where the *Lnc473*^ORF^ was under activity-responsive regulation (four cAMP response elements (CREs) and a minimal promoter (pmin), *i.e.*, 4 × *CRE*pmin; Fig. 2*E*). Specificity of the antibodies was confirmed by mutation of the translation initiation codon (*Lnc473*^mut^). This result and the ribosome profiling study (38) both raise the possibility that *Lnc473* has evolved into a protein coding gene in primates. To assess the evolutionary significance of the potential gain of translational capacity we performed ancestral sequence reconstruction of *Lnc473* using 22 dry-nosed primate *Lnc473* orthologs. This revealed that the *Lnc473*^ORF^ most likely emerged *de novo* in the common ancestor of Old World monkeys and apes but has been lost due to frame shift or translation initiation codon mutations independently of lineage in about half of extant Old World anthropoids (*e.g.*, in chimpanzee; Fig. 2*F*). Thus, the *Lnc473*^ORF^ has been subject to evolutionary turnover with stochastic persistence, implying that it probably has evolved neutrally (39). Additionally, we did not detect its expression in human induced pluripotent stem cell (iPSC)-derived neuronal precursors or neurons. Given these findings we suggest that, although intriguing, the role of the putative protein may be negligible and we thus returned our focus to the *Lnc473* transcript.

### Ectopic Lnc473 alters expression of epilepsy-associated genes and CREB targets

To study gain-of-function effects of the dry-nose primate-specific *Lnc473* in neurons, we started by performing an RNA-seq experiment. For this we infected mouse primary neurons with the rAAV encoding the human *Lnc473* and EGFP (rAAV/*Lnc473*) or only EGFP as a control (rAAV/EGFP). Before commencing RNA-seq we determined that ectopic *Lnc473* was expressed at higher levels in mouse neurons than endogenous *Lnc473* in human iPSC-derived neurons in basal conditions or when depolarized with a high potassium concentration-containing solution to induce *Lnc473* transcription (Fig. S2*A*). As is expected for the activity-regulated p*Lnc* (1), ectopic expression of *Lnc473* was responsive to AP firing (Fig. S2*A*). *Lnc473* RNA was nearly equally distributed in the nuclear and cytoplasmic compartments in mouse neurons, whereas the endogenous spliced transcript was more abundant in the cytoplasm and the unspliced *Lnc473* preRNA was exclusively nuclear in human neurons (Fig. S2*B*). We also confirmed that *Lnc473* expression increased neither basal cell death of mouse neurons nor their vulnerability to an excitotoxic insult (Fig. S2*C*).

**Figure 3:**
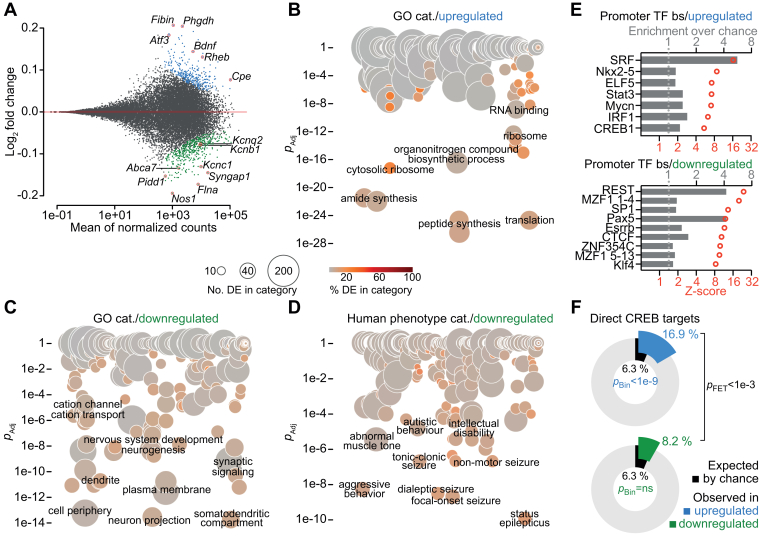
**Ectopic *Lnc473* alters expression of epilepsy-associated genes and CREB targets in mouse primary neurons.** Comparison of the transcriptomes of mouse primary neurons expressing either ectopic *Lnc473* and EGFP, or EGFP. RNA-seq analysis of the effect of *Lnc473* are shown. *A*, MA plot of gene expression changes determined with the DESeq2 package (106). *Blue* or *green dots* represent significantly (*p*_adj_ < 0.1, Benjamini–Hochberg correction) upregulated or downregulated genes, respectively. Some notable genes are marked and labeled. n = 4. *B* and *C*, bubble scatterplots show gene ontology category (GO cat.) overrepresentation among genes whose expression was regulated up (*B*) or down (*C*) as determined with the goseq package (107) (*p*_adj_, Holm correction). *D*, bubble scatterplot shows human phenotype category overrepresentation among downregulated genes as determined with g:Profiler (108) (*p*_adj_, Bonferroni correction). *B*–*D*, DE, differentially expressed. *E*, bargraph representation of results with oPOSSUM3 (109) for the enrichment of conserved transcription factor (TF) binding sites in promoters (±2 kb of TSS) of the upregulated or downregulated genes. The most significantly overrepresented sites are shown as fold enrichment over chance level. *F*, donut charts show proportions of direct CREB targets among the upregulated or downregulated genes. *p*_Bin_, Binominal tests (two-tailed *p*); *p*_FET_, Fisher’s exact test (two-tailed *p*). ns = not significant (*p* > 0.05). CREB, cAMP response element-binding protein; TSS, transcription start site.

Next, we studied the transcriptome of *Lnc473*-supplemented mouse cells. *Lnc473* expression produced subtle but clear differences, and we distinguished 290 upregulated and 414 downregulated genes (*p*_adj_ < 0.1, Fig. 3*A*, Tables S6 and S7). Notably, among the upregulated genes was the neurotrophin gene *Bdnf* and the sorting receptor gene *Cpe*, which encodes the carboxypeptidase E protein necessary for the activity-dependent secretion of Bdnf (40). Human orthologs of many of the downregulated genes, such as *Kcnc1*, *Kcnb1*, *Kcnq2*, *Flna*, *Nos1*, *Pidd1*, *Syngap1*, and *Abca7* for example, are associated with epilepsy, intellectual disability, psychiatric disorder, and/or Alzheimer’s disease (41, 42, 43, 44, 45, 46, 47, 48, 49, 50, 51). We analyzed gene ontology category overrepresentation among the differentially expressed genes (DEGs) to characterize the profile of effects potentially arising from changes to the neuronal transcriptome. The results suggested an increased demand for protein synthesis (Fig. 3*B* and Table S8), perhaps supported in part by Bdnf signaling and elevated levels of *Rheb* (Fig. 3*A*), which both can stimulate translation *via* activation of the mechanistic target of rapamycin complex 1 (52, 53). This was accompanied by a pattern corresponding to a reduced requirement for gene products targeted to neurites and/or membranes, including synapses (Fig. 3*C* and Table S9). Additionally, downregulated genes displayed a striking enrichment of human phenotype ontology categories that cover epilepsy in particular but include also intellectual disability and autistic behavior (Fig. 3*D* and Table S10). Genetic associations with epileptic seizure etiologies often originate from loss-of-function mutations that affect neuron or network excitability and result in hyperactivity (54), reflecting the potential consequences of reduced levels of such genes in *Lnc473*-expressing cells.

Transcriptional changes may expose altered regulatory activity. To investigate the possibility that *Lnc473* expression may have attuned the function of (a) certain transcription factor(s) we counted occurrences of conserved DNA *cis*-regulatory elements in the promoters (delimited here to ±2 kb of the transcription start site) of DEGs for overrepresentation analyses. We found enrichment of binding sites for several transcription factors (Fig. 3*E*, Tables S11 and S12). Serum response factor and RE1 silencing transcription factor motifs were significantly overrepresented, although not many promoters contained these sites (4 and 10 genes, respectively), which is typical of lengthy, rare DNA elements. Analogous in this regard was the notable enrichment of paired box protein 5 (Pax5) motif (three genes; Fig. 3*E*). Especially interesting was the prevalence of conserved binding sites of CREB in promoters of upregulated genes (45 motifs in 39 genes; Fig. 3*E*) because CREB-mediated transcription has been shown to be involved in *Lnc473* autoregulation in cancer (19) and cAMP/CREB signaling has been proposed to be altered by ectopic cortical *Lnc473* expression in female mice (17). Moreover, indicative of enhanced CREB activity, we found significantly more CREB targets among the upregulated genes in neurons expressing *Lnc473* than expected by chance (49 genes; Fig. 3*F* and Table S6).

Together, the transcriptomic changes in mouse neurons primatized with *Lnc473* expression suggest that *Lnc473* may modulate neuronal network activity, perhaps in part by accentuating CREB function (7, 55, 56) and/or resulting in accentuated CREB function (7, 57, 58).

### Lnc473 expression stimulates CREB-mediated transcription

**Figure 4:**
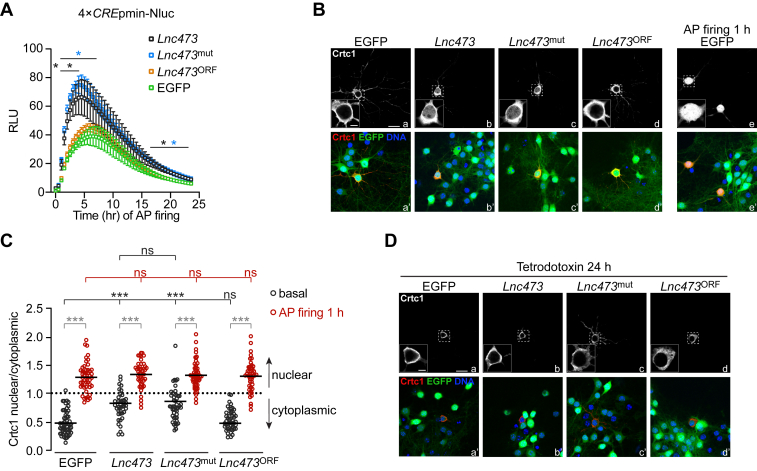
**Ectopic *Lnc473* RNA stimulates CREB-mediated transcription.** Mouse primary neurons were infected with either the rAAV encoding WT human *Lnc473* and EGFP (*Lnc473*), the rAAV encoding *Lnc473* with mutated translational start codon of its ORF (UniProt A8K010) and EGFP (*Lnc473*^mut^), the rAAV encoding *Lnc473* ORF (under control of p*SYN1*) and EGFP (*Lnc473*^ORF^), or the rAAV encoding EGFP (EGFP). Where indicated AP firing was induced with Bic (50 μM). *A*, results of a live reporter assay for CRE-dependent transcription. Coinfection with an rAAV encoding for the Nanoluc (Nluc) luciferase under the control of four CRE elements and a minimal promoter (4 × *CRE*pmin) with the indicated rAAVs was used. Luminescence was measured every 30 min for 24 h after addition of Bic. Relative light units (RLUs) were normalized to the mean baseline levels of EGFP-expressing control cells. *B*, representative immunocytochemistry results of Crtc1 localization in neurons infected with the indicated rAAVs. Infected neurons were transfected with a construct encoding for the mouse Crtc1 protein tagged with hemagglutinin (HA). Localization of Crtc1 was monitored in basal conditions and after 1 h of AP firing to stimulate its translocation into the nucleus. Images show HA immunofluorescence (insets a, b, c, d, and e) and HA immunofluorescence combined with EGFP and Hoechst fluorescence for concurrent detection of infection and cell nuclei, respectively (insets a’, b’, c’, d’, and e’). The effect of AP firing is shown only for EGFP-expressing cells, as this is representative of all groups. *C*, quantification of *B*. The ratio of the mean nuclear and cytoplasmic HA-Crtc1 immunofluorescence signal intensity was calculated for each transfected cell displaying EGFP fluorescence. *D*, same as *B* but neurons were treated with tetrodotoxin for 24 h to block AP firing. The scale bars in *B* and *D* represent 20 μm (*large images*) and 5 μm (*insets*). *Squares* in *A* and *lines* in *C* show means. Error bars in *A* denote ± SD. n = 3 with four replicates each (*A*) and n = 40 to 57 cells from three preparations (*C*). *A*, ∗*q* ≤ 0.05. Repeated measures two-way ANOVA (*F*_1.37, 10.95_ = 316.6, time; *F*_3, 8_ = 14.45, virus) and comparisons with the EGFP condition corrected by Benjamini and Hochberg FDR. *C*, ns = not significant, ∗∗∗*p* ≤ 0.001. Two-way ANOVA (*F*_3, 383_ = 14.76, virus; *F*_1, 383_ = 750, treatment) with Tukey's test. AP, action potential; CRE, cAMP response element; CREB, cAMP response element-binding protein; Crtc1, CREB-regulated transcription coactivator 1; rAAV, recombinant adeno-associated virus.

We used a live reporter assay to study the impact of *Lnc473* expression on CREB activity. To this end, we coinfected mouse primary neurons with rAAV/*Lnc473* (or rAAV/EGFP) and an rAAV encoding a *CRE*pmin-controlled Nanoluc (Nluc) luciferase. In addition, in this analysis we included the *Lnc473*^mut^ control virus and an rAAV encoding the *Lnc473* ORF under the control of p*SYN1* (*Lnc473*^ORF^) to test the potential role of the *Lnc473*^ORF^. AP firing was induced to better discern CREB activation. The assay distinguished *Lnc473* RNA-expressing and EGFP- or *Lnc473*^ORF^-expressing neurons, showing that irrespective of the ORF, the CREB-mediated transcriptional response to AP firing was enhanced by *Lnc473* RNA (Fig. 4*A*). CREB is predominantly activated by phosphorylation at serine 133 (S133) and recruitment of CREB binding protein (57), although CREB-regulated transcription coactivator (CRTC) nuclear translocation and binding to CREB, which can take place independently of the former, also promotes its function (59). We carried out Western blot analyses for CREB S133 phosphorylation and immunocytochemistry for localization of recombinant Crtc1 to find out the potential route of the observed effect. Whereas CREB phosphorylation at S133 was unaffected by *Lnc473*, *Lnc473*^mut^ or *Lnc473*^ORF^ (Fig. S3, *A* and *B*), we detected Crtc1 to be more nuclear in basal conditions in both WT *Lnc473*- as well as ORF-mutant *Lnc473*-expressing neurons than in the cells expressing EGFP or *Lnc473*^ORF^ (Fig. 4, *B* and *C*). One hour of AP firing triggered similar accumulation of Crtc1 into the nuclei in all cell groups analyzed (Fig. 4, *B* and *C*). We next investigated if the effect of *Lnc473* expression on CREB activity additionally includes a direct, local enhancement of the transcriptional coactivator capacity of Crtc1. A transfection-based assay with a constitutively nuclear Gal4 DNA binding domain (G4DBD)-Crtc1 fusion protein (Fig. S3*C*) driving expression of a reporter under the control of Gal4 binding sites revealed no differences between the cultures expressing the different viral constructs in basal conditions or after 4 h of AP firing (Fig. S3*D*). Together, these results indicate that, independent of its translational potential, *Lnc473* RNA expression enhances CREB-mediated transcription primarily by augmenting Crtc1 nuclear translocation, possibly without increasing RNA polymerase II recruitment to Crtc1 locally in the nucleus. Furthermore, the surge of Crtc1 into the nucleus was prevented by treatment of the cultures with the sodium channel blocker tetrodotoxin (TTX), confirming that this effect requires basal neuronal activity (Fig. 4*D*). Thus, CREB may be primed for activation by CRTC in the presence of *Lnc473* RNA in neurons.

### Lnc473 increases neuronal and network excitability

**Figure 5:**
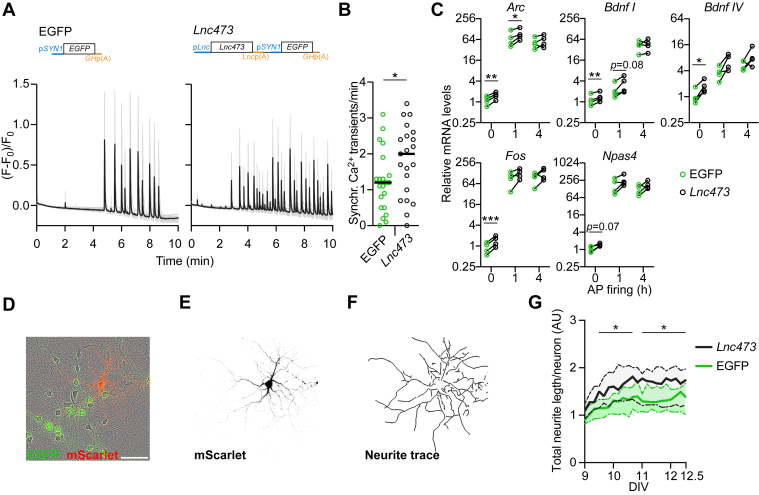
***Lnc473* expression promotes synchronous activity, its associated gene expression and neurite growth in cultures of mouse primary neurons.** Mouse primary neuron cultures expressing ectopic *Lnc473* and EGFP (*Lnc473*) or only EGFP (EGFP) were analyzed. *A*, representative results of imaging with the recombinant calcium indicator jRGECO1a introduced into cells by rAAV infection. *Gray traces* are from individual cells, *black traces* represent their average. *B*, quantification of *A* showing the frequency of synchronous somatic calcium transients per minute in unstimulated cultures during a 10-min imaging period. *C*, RT-qPCR analyses results of expression levels of the indicated genes in basal conditions or after induction of AP firing for 1 or 4 h with Bic (50 μM). mRNA levels were normalized to *GAPDH* expression and are plotted relative to the respective mRNA levels in uninfected cultures. *Bdnf I* and *IV* refer to *Bdnf* transcripts containing exon I or exon IV, respectively. *D*–*G*, at day *in vitro* (DIV) 7 the infected neurons were transfected with a plasmid encoding mScarlet. Starting at DIV 9, phase contrast images, and EGFP and mScarlet fluorescence images were obtained with the Incucyte system at 4 h intervals. mScarlet expression was used to generate traces for neurite length measurements. An example of a transfected neuron and its neurite trace in an rAAV/EGFP-infected culture is shown in *D*–*F*. Total neurite lengths per neuron were quantified and are presented in arbitrary units (AU) relative to EGFP-expressing cells at DIV 9 (*G*). The scale bar in *D* represents 100 μm and applies to *D*–*F*. *Lines* in *B* represent median. In *C*, *lines* connect data points from the same experimental replicate. In *D*, medians (*lines*) and interquartile ranges (*dashed lines*) are shown. n = 21 cultures from six independent preparations (*B*), n = 4 (*C*), and n = 22 wells (each containing ∼10–20 transfected neurons) from three independent preparations (*G*). *B* and *C*, ∗*p* ≤ 0.05, ∗∗*p* ≤ 0.01, ∗∗∗*p* ≤ 0.001. *B*, *t*_40_ = 2.12, two-tailed *t* test. *C*, *t*_3_ = 9.61, *Arc* 0 h; *t*_3_ = 4.36, *Arc* 1 h; *t*_3_ = 1.11, *Arc* 4 h; *t*_3_ = 8.75, *Bdnf I* 0 h; *t*_3_ = 3.46, *Bdnf I* 1 h; *t*_3_ = 0.48, *Bdnf I* 4 h; *t*_3_ = 5.83, *Bdnf IV* 0 h; *t*_3_ = 3.13, *Bdnf IV* 1 h; *t*_3_ = 1.66, *Bdnf I* 4 h; *t*_3_ = 23.5, *Fos* 0 h; *t*_3_ = 1.94, *Fos* 1 h; *t*_3_ = 1.47, *Fos* 4 h; *t*_3_ = 4.31, *Npas4* 0 h; *t*_3_ = 2.17, *Npas4* 1 h; *t*_3_ =1.84, *Npas4* 4 h; two-tailed ratio paired *t* tests with Holm-Sidak *p* adjustment. *G*, ∗*q* ≤ 0.05. Repeated measures two-way ANOVA (*F*_21, 882_ = 19.76, time; *F*_1, 42_ = 7.05, virus) and comparisons with the EGFP condition corrected by Benjamini and Hochberg FDR. rAAV, recombinant adeno-associated virus.

Synaptic activity stimulates CREB-mediated gene expression (57, 58, 60) and one of the key roles of this response is increasing intrinsic neuronal excitability (55, 56, 61). We thus hypothesized that the detected effects of *Lnc473* on gene regulation (Figs. 3 and 4) may be associated with enhanced excitatory activity, possibly involving altered excitability. To gain support for this idea, we first used calcium imaging in mouse hippocampal primary neuron cultures to detect synchronous network activity, which can be changed by altered excitability (62), and analyzed expression levels of prototypic activity-regulated genes as a proxy of neuronal activity. Fluorescence recordings of the virally-delivered recombinant calcium indicator jRGECO1a showed that while both EGFP- and *Lnc473*-expressing neurons displayed spontaneous synchronous periodic increases in intracellular calcium concentration, the frequency of these events was higher with *Lnc473* (Fig. 5, *A* and *B*), implying more activity. In line with this, *Lnc473* expression increased basal *Arc*, *Bdnf*, *Fos*, and *Npas4* levels (Fig. 5*C*). Induction of synaptic activity in the cultures by Bic treatment strongly stimulated the expression of the tested genes, whereas the effect of *Lnc473* was largely lost (Fig. 5*C*). These results indicate that *Lnc473* expression may promote network activity and facilitate the associated transcriptional responses, in which CREB has a major role (11, 63). As a consequence, *Lnc473* would be expected to affect structural development, since neuronal activity is known to stimulate dendrite outgrowth *via* CREB/CRTC (64, 65). Indeed, using live monitoring of primary neurons expressing the red fluorescent protein mScarlet to trace cell morphology (Fig. 5, *D*–*F*), we observed an increased total neurite length per neuron in *Lnc473*-expressing cultures at day *in vitro* (DIV) 9.5 to 12.5 (Fig. 5*G*). This conforms to a *Lnc473*-mediated rise in network activity and suggests a role for *Lnc473* in activity-dependent structural plasticity.

**Figure 6:**
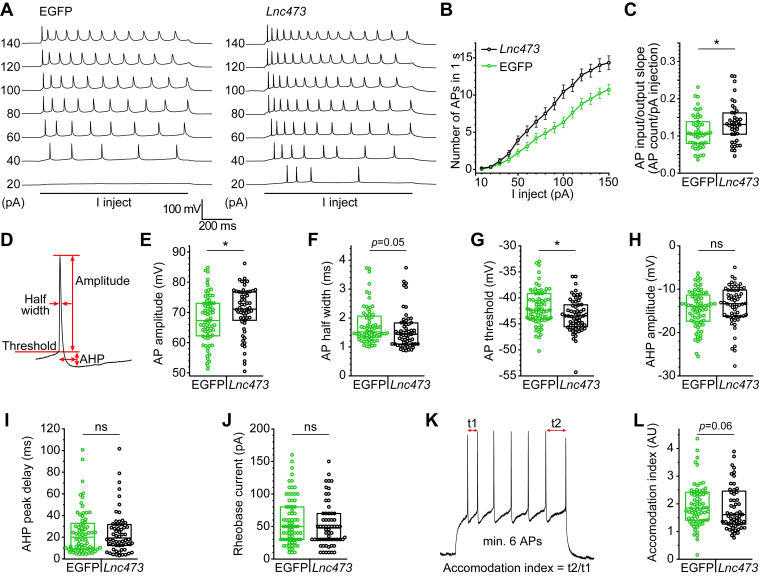
**Ectopic *Lnc473* increases excitability of mouse cultured neurons.** Whole cell patch clamp analyses of the effects of *Lnc473* expression on APs evoked by depolarizing current injection in mouse primary neurons. Cells infected with the rAAV encoding WT human *Lnc473* and EGFP (*Lnc473*) or with the rAAV encoding EGFP (EGFP) were compared. *A*, representative examples of APs evoked by 1 s of current injection (I inject) at the levels indicated (in pA) beside each trace. *B* and *C*, analysis of recordings similar to those in *A* quantifying the number of APs evoked by current injection steps. *D*, schematic illustration of AP parameter definitions. *E*–*I*, analysis of the indicated parameter of the first AP evoked by minimal current injection (*i.e.*, the rheobase current shown in *J*). *K*, schematic explanation of the quantification of the accommodation index shown in *L*. In *B*, means ± SEM are shown. All other plots show values from individual cells (*circles*), their median (*line*) and interquartile range (*box*). For n numbers see Table S13. ns = not significant, ∗*p* ≤ 0.05; Kolmogorov-Smirnov tests. AHP, afterhyperpolarization; AP, action potential; rAAV, recombinant adeno-associated virus.

To directly assess potential electrophysiological changes, we performed whole cell patch clamp recordings of mouse hippocampal primary neurons infected with rAAV/*Lnc473* or rAAV/EGFP. We found that whole cell capacitance, membrane resistance and resting membrane potential did not differ between the groups (Table S13 and Fig. S4, *A*–*C*). However, the number of APs generated in response to depolarizing current injection was increased, as was AP amplitude, indicating an increased excitability of *Lnc473*-expressing neurons (Fig. 6, *A*–*F*). This was further supported by a more negative AP threshold potential (Fig. 6*G*) and reduced AP accommodation (Fig. 6, *K* and *L*). Other AP and afterhyperpolarization (AHP) parameters were not altered (Fig. 6, *H*–*J*). To investigate the mechanism behind such effects we examined three channel types present in hippocampal excitatory neurons known to affect excitability and resting membrane potential—the hyperpolarization-activated cyclic nucleotide-gated (HCN) cationic channel, responsible for the h current, the potassium inward rectifier (Kir) channel, and Kv4.2, a member of the A-type potassium channel family (66, 67, 68, 69, 70, 71). *Lnc473*-expression did not, however, affect these conductances (Fig. S4, *D*–*F*). To explore potential effects on synaptic connectivity, we recorded miniature excitatory postsynaptic currents (mEPSCs) and examined their inter-event interval as well as amplitude, which were also not affected by *Lnc473*-expression (Fig. S4, *G*–*I* and Table S13). In summary, *Lnc473* expression enhanced AP generation as well as AP amplitude and brought the AP threshold potential closer to the resting membrane potential, demonstrating an increased excitability of mouse neurons in the presence of the primate-specific *Lnc473*.

## Discussion

Here, we found that ectopic expression of a higher primate-specific *Lnc473* RNA in mouse primary hippocampal cultures resulted in an elevated CREB-regulated transcriptional output that correlated with increased network activity and excitability of neurons. A possible interpretation of these effects is that *Lnc473* in primates may play a role in improving the ability of CREB to enhance intrinsic neuronal excitability (7, 55, 56, 61).

Expression of the mature, spliced *Lnc473* transcript is selectively preserved in higher primates, including humans. In thermogenic adipocytes this lineage-specific *Lnc473* expression was recently shown to regulate coupling of mitochondrial respiration and lipolysis (20). In addition, several lines of evidence suggest that it may also play a role in brain maturation and function (1, 14, 17), which appears to have evolved with an oncogenic trade-off (*e.g.*, (19, 28, 30)). As in cancer (19), we found that also in neurons *Lnc473* accentuates CREB-dependent transcription. However, we can conclusively attribute this effect to a spliced *Lnc473* transcript, whereas in cancerous cells the bulk of *Lnc473* remains unspliced (72) and may be functional especially at its own gene locus (17, 19) where it is readily detectable (17, 19, 72). Our data suggests that the mature *Lnc473*, which is located in the cytoplasm and the nucleus in neurons, affects CREB function by facilitating activity-dependent nuclear accumulation of the CREB coactivator CRTC, possibly involving interaction with the nuclear non-POU domain containing octamer binding protein Nono (19, 73). Considering the detected increases in excitability and neurite length, and the role of CREB in regulating both (7, 55, 56, 61, 64, 65, 74), we hypothesize that *Lnc473* expression in mouse neurons could have launched a primate-specific positive feedback loop of CREB activation that stimulates a rise in excitability and excitatory network activity, which in turn fosters CRTC nuclear translocation. This idea is further supported by the finding that the cAMP/CREB pathway may indeed be perturbed in presence of *Lnc473* expression also in the mouse brain cortex *in vivo* (17). Note, however, that these observed effects have been produced by chronic high levels of ectopic *Lnc473*, thus they likely just reveal the directions of the fine-tuning actions of endogenous *Lnc473* in primate neurons, where it reaches high levels only after strong synaptic stimulation (1). Moreover, prolonged neuronal activity may induce protein expression from the *Lnc473* RNA in humans ((38), Fig. 2, *E* and *F*) and other primates with an intact *Lnc473* ORF. Although the apparently stochastic evolutionary retention of the *Lnc473*^ORF^ in primates (present in, *e.g.*, humans and gorillas, but not in, *e.g.*, chimpanzees) suggests that this protein is functionally inert or trivial, species-dependent modulatory roles of the *Lnc473*^ORF^ cannot be ruled out.

The impact of ectopic *Lnc473* expression on intrinsic and network excitability is supported by the detected transcriptomic shift. CREB target genes *Bdnf* (75, 76) and *Rheb* (77) that were upregulated by *Lnc473*, are both implicated in neuronal hyperactivity and epileptogenesis (78, 79, 80), and, additionally, can themselves stimulate CREB (81, 82, 83). Moreover, we found extensive downregulation of genes that, if mutated in human, cause seizures by diverse mechanisms, including perturbed cation channel conductance and synaptic signaling (*e.g.*, *Kcnc1* (42), *Kcnb1* (50), *Kcnq2* (51), *Cacna1a* (84, 85), *Dnm1* (86), *Pcdh19* (87), *Syngap1* (45); Table S14), substantiating a wide-ranging repertoire of modulatory factors that may affect excitability. In line with this we found increased intrinsic excitability (*i.e.*, increased AP generation in response to prolonged depolarization) contradicting previous evidence that *Lnc473* expression does not change excitability (17). While spontaneous EPSC frequency and amplitude were shown to be increased by *Lnc473* expression in female but not male mice (17), such synaptic activity is largely AP-mediated and, thus, may reflect a gender-specific rise in spontaneous presynaptic AP activity. However, the lack of effect on mEPSCs in our study indicates no effect of ectopic *Lnc473* expression on excitatory synaptic strength, connectivity, and/or release probability.

In the cerebral cortex of schizophrenics, *Lnc473* expression is downregulated together with an activity-regulated gene module that contains many CREB targets, including *BDNF* (18). Our *Lnc473* gain-of-function results in mouse neurons are in line with these data, implying a role for *Lnc473* in enhancing neuronal activity and CREB function. A CREB-regulated increase in excitability is required for memory allocation to ensembles of neurons activated in synchrony during learning (88, 89, 90). Intriguingly, formation of cortical ensembles is impaired in mouse models of schizophrenia (91) and neuronal synchrony in cortical activation is deficient in schizophrenia patients, resulting in “noisy” microcircuit activity and memory malfunction (92, 93, 94, 95). As a higher primate-specific additional positive modulator of CREB-mediated excitability, *Lnc473* may introduce a refinement mechanism into the regulation of functional neuronal connectivity. If this fails, however, it can possibly contribute to alterations in cognitive functions. Thus, the human *Lnc473*, whose copy number variations are associated with schizophrenia (16), is emerging as an example for the idea that the genetic origins of advanced social cognitive abilities and of predisposition to mental illness overlap (96, 97, 98). To our knowledge *Lnc473* is also the first example of a human lineage-specific gene that alters the electrophysiological properties of a neuron. This suggests that priming of neurons for activity-dependent upregulation of excitability, perhaps in conjunction with an enhancement of structural plasticity as shown here, may have contributed to evolutionary advancements of brain functions in higher primates. Appearance of the *Lnc473* gene complements these uniquely human genetic changes that have previously been identified to increase the proliferative capacity of neuronal precursor cells or the synaptic density and connectivity of neurons (99).

In summary, we showed that *Lnc473* expression in neurons leads to increased excitability and CREB activity. The results encourage further investigations of the role of *Lnc473* in cognitive functions, particularly in memory, and its dysfunction in schizophrenia.

## Experimental procedures

### Differential exon usage analysis

Paired-end RNA-seq reads from mouse primary neuron and human iPSC-derived neuron coculture samples (1) were aligned to GRCh38/hg38 or GRCm38/mm10 with Bowtie2 (2.2.6.2) (100). To remove pairs with one aligned read, the unaligned pairs were joined and split again using the FASTQ joiner and FASTQ splitter tools, respectively (101). Genome-unaligned paired-end reads were aligned to either hg38 or mm10 transcriptomes (UCSC Genes/knownGene), respectively, with Bowtie2. Reads not aligned to human genome and transcriptome, or not aligned to mouse genome and transcriptome, were then aligned to gene model annotations of mm10 or hg38, respectively, with HISAT2 (2.1.0) (102). Exon abundancies were estimated using DEXSeq-Count (1.28.1.0). Differential exon usage of human *PDE10A* and mouse *Pde10a* was tested with DEXSeq (1.28.1) (103). All tools were used *via* the Galaxy platform (104). Bonferroni correction was applied for multiple testing of differential usage of *PDE10A* or *Pde10a* exons.

### RNA sequencing and data analysis

Poly(A)^+^ RNA was isolated from mouse hippocampal neuron total RNA (obtained with the RNeasy Mini Kit (Qiagen) and on-column DNase digestion) with the NEBNext Poly(A) mRNA Magnetic Isolation Module (New England Biolabs). cDNA libraries were prepared with NEBNext Ultra Directional RNA Library Prep Kit for Illumina (New England Biolabs). NEBNext Multiplex Oligos for Illumina (New England Biolabs) were used to provide adaptors and permit multiplexing. This preparative work was performed by Heidelberg University Deep Sequencing Facility. Sequencing was conducted in EMBL Genomics Core Facility with NextSeq 500 (Illumina) to obtain 75 bp single-end reads. Reads were mapped to the mouse genome assembly GRCm38/mm10 with HISAT2 (2.1.0) (102). Mapped reads per gene were counted with HTSeq-count (0.6.1) (105) and DESeq2 (2.11.40.6) (106) was used to determine DEGs. Overrepresentation of gene ontology categories among DEGs was tested with the goseq tool (1.44.0) (107) using the Wallenius method with Holm *p* adjustment. As the background, all genes detected to be expressed in the primary culture samples were used. All these tools were used *via* the Galaxy platform (104). Overrepresentation of human phenotype categories was tested with the g:GOSt functional profiling tool within g:Profiler (108) using only annotated genes and Bonferroni *p* correction. Again, all genes detected to be expressed in the primary culture samples were used as the background. Enrichment analysis of conserved transcription factor binding sites in the promoters of DEGs was performed with oPOSSUM3 (109) in single site analysis mode using JASPAR core vertebrate transcription factor binding site profiles, all genes detected to be expressed in the primary culture samples as background, and the conservation cutoff at phastCons score of 0.6. Promoters were defined as ±2 kb genomic DNA relative of the transcription start sites. For the analysis of CREB target gene abundancies, a list of 1247 CREB-regulated genes with experimental evidence was combined from the studies noted next to Table S6.

### Conservation assessment of the *PDE10A* gene locus and *Lnc473* transcripts

We used the human *PDE10A* gene coordinates of GRCh38/hg38 in the UCSC genome browser (https://genome.ucsc.edu). Convert tool was used to display and retrieve corresponding sequences from different assemblies or orthologous sequences from other species. In sequence conservation evaluation we used the Multiz alignments and Basewise conservation by phyloP tracks. Phylogenetic footprinting with the ORCA toolkit (110) was used to identify conserved transcription factor binding sites in noncoding regions of the genomic sequence corresponding to the *PDE10A* gene 5′ end that includes the promoter of the *Lnc473* AK289375 transcript. Splice branch points were scored with SVM-BPfinder (111), poly(A) signals with Poly(A) Signal Miner (112). Sequence alignments and Newick tree generation were performed with Clustal Omega and Simple Phylogeny tools (113) using neighbor-joining clustering. Ancestral sequence reconstruction of the *Lnc473* ORF was performed with the GRASP-suite (95). Phylogenetic trees were displayed and customized with the iTOL tool (114).

### 3′ rapid amplification of cDNA ends

An oligo(dT) primer linked with an adapter sequence (5′-GGC CAC GCG TCG ACT AGT ACT TTT TTT TTT TTT T-3′) was used to synthesize cDNA with the SuperScript III Reverse Transcriptase (Thermo Fisher Scientific) using total RNA from human fetal brain (Agilent, #540157) or human iPSC-derived neuronal precursor cells or neurons (from the iPSC line D1). A detailed protocol of neuronal precursor cell and neuron differentiation from iPSCs as well as their characterization has been published before (1). A *Lnc473*-specific sense primer (5′-GTA CAC GCG TGT CTA CGT GCT ATA GCC TGG AAA TG-3′) and an adapter-targeting antisense primer (5′- GGC CAC GCG ACT AGT AC-3′) were used to perform *Lnc473* AK289375 transcript-specific 3′ rapid amplification of cDNA ends. PCR products were visualized by agarose gel electrophoresis and cloned. Poly(A) sites were verified by sequencing to map to the AK289375 transcript poly(A) cluster that has been detected as the primary transcription termination site for such transcripts also in other human cell types (35) (see Fig. 2*B*).

### Mouse hippocampal primary cell cultures

Newborn C57BL/6N mice were used to prepare hippocampal primary cell cultures mixed of male and female mouse cells. Cells were plated with the density of ∼80,000/cm^2^ and were first kept in Neurobasal-A (Life Technologies) with B27 (Life Technologies), 0.5 mM L-glutamine, and 1% rat serum. On DIV 8 the medium was changed to a salt-glucose-glycine solution [10 mM Hepes (pH 7.4), 1 mM glycine, 26.1 mM NaHCO_3_, 5.3 mM KCI, 1 mM MgCI_2_, 2 mM CaCI_2_, 30 mM glucose, 114 mM NaCI, 0.5 mM sodium pyruvate] and Eagle’s minimum essential medium (vol:vol 9:1), with insulin, transferrin, and sodium selenite (ITS, all 7.5 ng/ml, Sigma-Aldrich). Experiments were performed at DIV 10 or 11. Bic (50 μM) was used to induce AP firing and N-methyl-D-aspartate (30 μM) to induce excitotoxic neuron death.

### rAAV constructs

For ectopic *Lnc473* expression the human *Lnc473* AK289375 transcript cDNA together with its promoter and poly(A) cluster region was cloned into an rAAV vector. The final *Lnc473*-ecoding rAAV plasmid contained the human genomic regions spanning hg38 chr6:165,987,529-165,988,752 (reverse strand, promoter, and the first exon) followed by chr6:165,939,377-165,940,766 (reverse strand, the second exon and poly(A) sites). The rAAV produced with this construct is named rAAV/*Lnc473*. Note that this virus also expresses EGFP (see below). To generate *Lnc47*3^mut^ the ATG start codon of the *Lnc473* ORF (hg38 chr6:165,940,198-165,940,755, reverse strand) in the rAAV/*Lnc473* construct was mutated to AGG (rAAV/*Lnc473*^mut^). To generate the rAAV/*CRE*p-*Lnc473*^ORF^ construct, the ORF of *Lnc473* was cloned downstream of four CRE elements and a TATA-box minimal promoter (4 × *CRE*pmin), obtained from pGL4.29[luc2P/CRE/Hygro (Promega) and upstream the human growth hormone poly(A) signal. For the rAAV/*Lnc473*^ORF^ construct the ORF of *Lnc473* was cloned between p*SYN1* and human growth hormone poly(A). A p*SYN1*-driven *EGFP* cassette (p*SYN1*-*EGFP*) was present downstream of each *Lnc473* construct. To generate a viral luciferase reporter for CRE-mediated transcription, 4 × *CRE*pmin was cloned in front of Nluc in pNL1.2[NlucP] (Promega) and the 4 × *CRE*pmin-NlucP cassette was cloned from there into an rAAV vector (to obtain rAAV/4 × *CRE*pmin-NlucP). All rAAV plasmids were verified by sequencing. Viral particles (serotype AAV1/2) produced in AAV-293 cells (Stratagene, #240073) were purified by affinity chromatography (115). A p*SYN1*-*EGFP*-encoding rAAV (rAAV/EGFP) was produced to be used as a control in all experiments. A p*SYN1-jRGECO1a*-ecnoding rAAV was produced for calcium imaging. Mouse primary neurons were infected at DIV 3. EGFP levels were used to titer ectopic expression. Infection rate was >90%.

### Western blotting

An affinity-purified *Lnc473* ORF ab was produced in rabbit by GL Biochem Ltd (product ID: AB007693) with the peptide Cys-QAPWREFTGRHRTE as the antigen. For Western blotting cultured neurons in 4-well plates (Thermo Fisher Scientific, #176740) were collected into 100 μl SDS sample buffer (160 mM Tris–HCl (pH 6.8), 4% SDS, 30% glycerol, 10 mM dithiothreitol, and 0.02% bromophenol blue) and boiled at 95 °C for 5 min. Fifteen μl of samples were separated by 10% or 12% SDS-polyacrylamide gel electrophoresis and transferred onto 0.45 μm nitrocellulose membranes (Amersham, Protran #10600002). The membranes were blocked with blocking buffer containing dry milk (5% non-fat dry milk in PBS + 0.1% Tween-20) or, when probing for phospho-CREB, containing bovine serum albumin (BSA) (5% BSA in PBS + 0.05% Tween-20) and probed with a primary ab (reported as “ab”, “dilution in blocking buffer”): α-*Lnc473*^ORF^, 1:200; rabbit α-Junb (Cell Signaling Technology, #3753), 1:1000; rabbit α-GFP (Stratagene, #240142), 1:20,000; mouse α-tubulin-alpha (Sigma Aldrich, #T9026), 1:20,000; mouse α-CREB Phospho-Ser133 (Millipore, #05-667), 1:1000; or rabbit α-CREB (Cell Signaling Technology, #4820), 1:2000. Horseradish peroxidase-conjugated secondary abs were applied to prepare the membranes for protein detection with chemiluminescence. Goat α-rabbit IgG (Dianova #111-035-144, 1:5000) or α-mouse IgG (Dianova #115-035-003, 1:5000) and the Clarity Western ECL (Bio-Rad #1705060) reagents were used. Western blot signal intensities were quantified in ImageJ (https://imagej.nih.gov) using lane profile plots.

### RT-qPCR analyses of RNA levels and RNA localization

Total RNA was isolated with the RNeasy Mini Kit (Qiagen) with on-column DNase digestion. For RNA isolation separately from nuclear and cytoplasmic fractions the PARIS Kit (Thermo Fisher Scientific) was used, followed by DNase treatment using the Turbo DNA-free kit (Invitrogen). Human iPSC-derived neuron RNA was isolated from cells differentiated as described before (1). cDNA was produced with the SuperScript III Reverse Transcriptase (Thermo Fisher Scientific). All quantitative polymerase chain reaction (qPCR) reactions were performed with the Power SYBR Green Master Mix (Thermo Fisher Scientific) in the StepOnePlus Real-Time PCR System (Applied Biosystems). The following primer pairs were used: *Lnc473*, 5′-GAA CTC GAA ATG AAG CGG AAA G-3′ and 5′-GCA GCC GAC AGT TCC AT-3′; *Lnc473* preRNA, 5′-AGT GAT AAA GGC TGC TGA ATT GTG CT-3′ and 5′-GCA GCC GAC AGT TCC AT-3′; *GAPDH*, 5′-CAA AAT CAA GTG GGG CGA TGC T-3′ and 5′-TTG GCT CCC CCC TGC AAA TGA-3′; *NEAT1*, 5′-TGT GTG TGT AAA AGA GAG AAG TTG TGG-3′ and 5′-GTG AGA GCT GGG TGC CT-3′; *GAPDH*, 5′-CAC TCT TCC ACC TTC GAT GCC-3′ and 5′-GGG TGG GTG GTC CAG GGT T-3′; Malat, 5′-CAC CAG TGG ACA AAA TGA GGA-3′ and 5′-AAA AGG CTT AGC GCC CAC C-3′; *Arc*, 5′-AGA CCT GAC ATC CTG GCA CC-3′ and 5′-GCT CTG CTC TTC TTC ACT GGT A-3′; *Bdnf I*, 5′-AAC AAG ACA CAT TAC CTT CCT GCA T-3′ and 5′-CTC TTC TCA CCT GGT GGA ACA TT-3′; *Bdnf IV*, 5′-GCT GCC TTG ATG TTT ACT TTG A-3′ and 5′-GCA ACC GAA GTA TGA AAT AAC C-3′; *Fos*, 5′-GGC AGA AGG GGC AAA GTA GAG-3′ and 5′-TGT CAG CTC CCT CCT CCG ATT C-3′; and *Npas4*, 5′-CAG GGC GAC AGT ATC TAC GAT-3′ and 5′-CAA CGG AAA AGG CGA TCA GCA-3′. For the analysis of nuclear-cytoplasmic distribution of RNA, cDNA was synthesized from proportionally equal volume of nuclear and cytoplasmic RNA. qPCR Ct values were used to estimate relative abundances of the respective gene products in nuclear and cytoplasmic samples. For statistical analysis and SD calculation, proportion data was arcsine transformed. For presentation the data were back-transformed.

### Estimation of the proportion of dead cells in cultures

Cultures of mouse primary neurons on glass coverslips were fixed with 4% paraformaldehyde, washed with PBS, and stained with Hoechst 33258 (Serva; 1 μg/ml). After mounting in Mowiol (Merck) nuclei were visualized by Hoechst fluorescence and counted. Shrunken nuclei were considered to be dead cells.

### Luciferase reporter assay of CRE-mediated transcription

Mouse primary neurons, plated into transparent flat-bottom white-walled 96-well tissue culture plates (Greiner Bio-One), were coinfected at DIV 3 with rAAV/4 × *CRE*pmin-NlucP and either rAAV/*Lnc473*, rAAV/*Lnc473*^mut^, rAAV/*Lnc473*^ORF^, or rAAV/EGFP. At DIV 10, Nano-Glo Endurazine (Promega) was added into the medium at a 1:200 dilution. Then, after 8 h Bic (50 μM) was added and luminescence from live cultures was measured every 30 min for 24 h at 37 °C with the EnSpire Plate Reader (PerkinElmer). All data were normalized to the baseline means of rAAV/EGFP infections.

### Analysis of Crtc1 subcellular localization

Mouse primary neurons on glass coverslips in 4-well plates were infected at DIV 3 with either rAAV/*Lnc473*, rAAV/*Lnc473*^mut^, rAAV/*Lnc473*^ORF^, or rAAV/EGFP. At DIV 8, the cells were transfected with a plasmid encoding human cytomegalovirus (CMV) promoter (pCMV)-driven mouse Crtc1 N-terminally tagged with the human influenza hemagglutinin (HA) epitope using Lipofectamine 2000 (LF) (Invitrogen; 0.5 μg DNA per well, 1:2 DNA (μg) to LF (μl) ratio). At DIV 10 the cells were either left untreated or treated with Bic (50 μM) for 1 h and fixed with 3% paraformaldehyde and 3% sucrose in PBS. Then, the cells were washed with PBS and treated with 50 mM NH_4_Cl in PBS, permeabilized with 0.3% Triton X-100 in PBS, blocked with blocking buffer (2% BSA in PBS + 0.1% Tween-20), and probed with a mouse α-HA-Tag ab (Cell Signaling Technology, #2367), diluted 1:500 in blocking buffer. After washes with PBS + 0.1% Tween-20, an Alexa594-conjugated goat α-mouse IgG secondary ab (Dianova #115-585-146, 1:1000 in blocking buffer) was applied. After washes with PBS + 0.1% Tween-20 the cells were mounted in Mowiol (Merck) containing Hoechst 33258 (Serva; 1 μg/ml). Confocal images of EGFP, Alexa594, and Hoechst florescence were obtained. Somatic HA staining signal intensities in nuclei and outside nuclei in EGFP-expressing cells were quantified with ImageJ. Nuclei were defined by their Hoechst signal. Relative distribution of HA-Crtc1 in each cell is presented as ratio between the mean nuclear signal intensity and the mean cytoplasmic signal intensity.

### Luciferase reporter assay of nuclear Crtc1 function

Mouse primary neurons in 48-well plates were infected at DIV 3 with rAAV/*Lnc473*, rAAV/*Lnc473*^mut^, rAAV/*Lnc473*^ORF^, or rAAV/EGFP. At DIV 8 the cells were transfected with a plasmid encoding the Nluc reporter (Promega) under the control of four G4DBD-binding *cis*-elements and pmin (4 × *UAS*pmin-NlucP; 0.25 μg/well) and either pCMV-G4DBD-Crtc1 or pCMV-Crtc1 (both 0.25 μg/well), together with a firefly luciferase (FFluc)-encoding normalizer construct pEF1α-FFlucP (0.02 μg/well) (1:2 DNA (μg) to LF (μl) ratio). After 2 days the cells were either left untreated or stimulated with Bic for 4 h. Cells were lysed with the Passive Lysis Buffer (Promega, 50 μl/well) and 20 μl of each lysate was used to perform the Nano-Glo Dual-Luciferase Reporter Assay (Promega) according to the instructions of the manufacturer. Luminescence was measured with the CLARIOstar^*Plus*^ plate reader (BMG Labtech).

### Calcium imaging

The genetically encoded red calcium indicator jRGECO1a under the control of p*SYN1* (a gift from Rolf Sprengel) was delivered into mouse primary neurons on glass coverslips by rAAV infection at DIV 3. Images of jRGECO1a fluorescence in unstimulated cells were obtained at DIV 10 at room temperature, at the rate of 2 Hz for 10 min. One recording per coverslip was performed. Data is shown as (F − F_0_)/F_0_ where F represents the mean background-subtracted fluorescence intensity in a region of interest (*i.e.*, a cell soma) and F_0_ is the baseline background-subtracted fluorescence in the same region of interest. As a control, after the 10 min recording time, addition of Bic (50 μM) was used to trigger synchronous periodic transient increases in calcium concentration, caused by AP bursting activity. Transient increases in somatic calcium concentration in the unstimulated condition were considered synchronous events if the mean peak jRGECO1a fluorescence intensity in all cells in the imaging field (∼15–30 cells) was at least 1/10 of that measured for the first calcium transient after addition of Bic.

### Analysis of neurite length

Mouse primary neurons in 24-well plates were infected at DIV 2 with rAAV/*Lnc473* or rAAV/EGFP. At DIV 7, transfection with a p*SYN1*-mScarlet plasmid was performed (0.5 μg DNA/well, 1:2 DNA (μg) to LF (μl) ratio). Plates were transferred into an Incucyte (Sartorius) live cell imaging incubator and phase contrast as well as EGFP and mScarlet fluorescence images were obtained at 4 h intervals during DIV 9 to 12.5. mScarlet expression and the Incucyte Neurotrack Analysis Software Module (Sartorius; https://www.sartorius.com/en/products/live-cell-imaging-analysis/live-cell-analysis-software/incucyte-neurotrack-analysis-software) with Top-Hat cell body cluster segmentation, 5 μm minimum cell width cleanup, 100 μm^2^ minimum cell body area filter, and neurite coarse and fine sensitivity set at 9 and 0.7, respectively, were used to define cell somas and generate neurite traces of transfected neurons. In each well total neurite length per cell was quantified.

### Electrophysiology recordings and analysis

Whole-cell patch clamp recordings were made from one neuron per coverslip. Coverslips were secured with a platinum harp in the recording chamber (OAC-1; Science Products) and submerged in continuously flowing (3 ml/min) artificial cerebrospinal fluid (in mM: NaCl, 125; KCl, 3.5; CaCl_2_, 2.4; MgCl_2_, 1.3; NaH_2_PO_4_, 1.2; glucose, 25; NaHCO_3_, 26; gassed with 96% O_2_ and 4% CO_2_) maintained at 32 to 34 °C with an in-line perfusion heater (TC324B; Warner Instruments). Patch electrodes (3–5 MΩ) were made from 1.5 mm borosilicate glass and filled with a potassium-based internal solution (in mM: Kgluconate, 118; EGTA, 0.2; Hepes, 10; K_2_Phosphocreatine, 10; KCl, 20; NaCl, 10; Mg-ATP, 2; Na_3_-GTP, 0.3). Recordings were made with a Multiclamp 700A or 700B amplifier, digitized through a Digidata 1550B A/D converter, and acquired and analyzed using pClamp 10 and 11 software (Molecular Devices, www.moleculardevices.com). Passive properties were monitored throughout the recordings and stabilized values were used for analysis. Pipette access resistance was maintained below 25 MΩ, range: 10 to 25 MΩ. Voltage clamp recordings were made with 20 kHz sampling and a low-pass filtering of 2 kHz. Current clamp recordings were made with a sampling frequency of 250 kHz and a low-pass filter of 10 kHz. Pipette, but not whole cell capacitance, was compensated in all recordings. Rheobase current (the minimum current injection needed to evoke an AP), AP threshold, AP amplitude, half width, AHP amplitude, and AHP delay to peak were assessed from the first AP evoked by 1 s depolarizing current injection steps applied in 5 or 10 pA increments from the resting membrane potential. Spontaneous APs or any AP coinciding with current injection onset were excluded. AP threshold was taken as the point where the first derivative of the voltage trace exceeded 20 mV/ms. AP and AHP amplitude and AHP delay were calculated relative to this threshold. Accommodation index was calculated as the ratio of the time interval between the first and last pairs of APs in response to a 1 s current injection evoking at least six APs. AP input/output functions were generated using identical current injection steps but from a membrane potential of approximately −70 mV maintained by constant current injection. HCN and Kir channels were activated with 1 s hyperpolarizing steps from −60 to −130 mV in 10 mV increments in the presence of TTX (0.5 μM) and picrotoxin (30 μM, Hello Bio). Hyperpolarization-activated currents were measured directly following the voltage step-induced capacitive transient (instantaneous current) and at the end of the step (steady state current). Linear fitting was used to calculate slope conductances. The Kir conductance was quantified as the difference between the instantaneous slope conductance at −70 to −90 mV and −110 to −130 mV. The HCN conductance was quantified as the slope of the difference between the instantaneous and steady-state currents in the range −110 to −130 mV. mEPSCs were recorded at −80 mV in the presence of TTX (0.5 μM) and picrotoxin (30 μM). The complete blockade of mEPSCs by the AMPA receptor inhibitor 2,3-dioxo-6-nitro-1,2,3,4-tetrahydrobenzo[f]quinoxaline-7-sulfonamide disodium salt (10 μM, Biotrend) was confirmed in five cells. mEPSC data were obtained from recordings of at least 200 events detected using the Mini Analysis Program (Synaptosoft). All events were visually verified and events occurring less than 10 ms after the previous event exhibited summation and were excluded from the amplitude but not the frequency analysis. Depolarization activated K^+^ currents were assessed from 500 ms long depolarizing steps from −100 mV to potentials between −60 mV and +35 mV in 5 mV increments. These current families were recorded in TTX (0.5 μM), picrotoxin (30 μM), verapamil (30 μM, Biotrend), and tetraethylammonium (5 mM, Sigma). Recordings were repeated after the addition of the potassium channel blocker 4-aminopyridine (5 mM) and digitally subtracted from the previous recording to isolate the tetraethylammonium insensitive, 4-aminopyridine sensitive, rapidly inactivating outward current, which peaked 3 to 13 ms after depolarization in a voltage-dependent manner. This current is mediated by an A-type delayed rectifier K^+^ channel most likely encoded by Kv4.2 and its conductance was quantified as the slope of its current–voltage relationship between −20 and +35 mV. All membrane potentials have been corrected for the calculated junction potential of 10 mV.

### Statistics and reproducibility

Data formats and details about the applied statistical methods as well as their outcomes are reported in the legend of each figure. For experiments with bulk samples (RNA-seq, RT-qPCR, Western blotting, luciferase assay), n denotes the number of independent neuron culture preparations. In calcium imaging or in single cell analysis (immunocytochemistry, electrophysiology), n denotes numbers of wells of cultures or cells, respectively; a statement about the number of independent culture preparations is added. The experimenter was not blinded to sample identities. Statistical analyses and plotting of data other than electrophysiology were performed using Prism version 8.2.1 (GraphPad; https://www.graphpad.com). For electrophysiology data OriginPro 2016 or 2022 (OriginLab, www.originlab.com) was used. Shapiro–Wilk tests were applied for normality. In some sets of the electrophysiology data normality was rejected (*p* < 0.05) and thus nonparametric statistics (median ± interquartile range) and hypothesis testing (Kolmogorov–Smirnov tests for independent samples) were used.

## Data availability

RNA-seq data that support the findings of this study are available in Gene Expression Omnibus with the identifiers GSE88773 (for *PDE10a*/*Pde10a* exon usage) and GSE201643 (for the effect of *Lnc473* expression in mouse neurons).

## Supporting information

This article contains supporting information (1, 42, 50, 51, 63, 84, 85, 86, 87, 103, 106, 116, 117, 118, 119, 120, 121, 122, 123, 124, 125, 126, 127, 128).

## Conflict of interest

The authors declare that they have no conflicts of interest with the contents of this article.

## References

[1] Pruunsild P., Bengtson C.P., Bading H. (2017). Networks of cultured iPSC-derived neurons reveal the human synaptic activity-regulated adaptive gene program. Cell Rep..

[2] Ataman B., Boulting G.L., Harmin D.A., Yang M.G., Baker-Salisbury M., Yap E.L. (2016). Evolution of osteocrin as an activity-regulated factor in the primate brain. Nature.

[3] Boulting G.L., Durresi E., Ataman B., Sherman M.A., Mei K., Harmin D.A. (2021). Activity-dependent regulome of human GABAergic neurons reveals new patterns of gene regulation and neurological disease heritability. Nat. Neurosci..

[4] Barco A., Marie H. (2011). Genetic approaches to investigate the role of CREB in neuronal plasticity and memory. Mol. Neurobiol..

[5] Lin Y., Bloodgood B.L., Hauser J.L., Lapan A.D., Koon A.C., Kim T.K. (2008). Activity-dependent regulation of inhibitory synapse development by Npas4. Nature.

[6] Sun X., Lin Y. (2016). Npas4: linking neuronal activity to memory. Trends Neurosci..

[7] Benito E., Barco A. (2010). CREB's control of intrinsic and synaptic plasticity: implications for CREB-dependent memory models. Trends Neurosci..

[8] Hardingham G.E., Bading H. (2010). Synaptic versus extrasynaptic NMDA receptor signalling: implications for neurodegenerative disorders. Nat. Rev. Neurosci..

[9] Hensch T.K. (2004). Critical period regulation. Annu. Rev. Neurosci..

[10] West A.E., Greenberg M.E. (2011). Neuronal activity-regulated gene transcription in synapse development and cognitive function. Cold Spring Harb. Perspect. Biol..

[11] Yap E.L., Greenberg M.E. (2018). Activity-regulated transcription: bridging the gap between neural activity and behavior. Neuron.

[12] Bading H. (2013). Nuclear calcium signalling in the regulation of brain function. Nat. Rev. Neurosci..

[13] Marin O. (2016). Developmental timing and critical windows for the treatment of psychiatric disorders. Nat. Med..

[14] Hardingham G.E., Pruunsild P., Greenberg M.E., Bading H. (2018). Lineage divergence of activity-driven transcription and evolution of cognitive ability. Nat. Rev. Neurosci..

[15] Pruunsild P., Bading H. (2019). Shaping the human brain: evolutionary cis-regulatory plasticity drives changes in synaptic activity-controlled adaptive gene expression. Curr. Opin. Neurobiol..

[16] Tam G.W., van de Lagemaat L.N., Redon R., Strathdee K.E., Croning M.D., Malloy M.P. (2010). Confirmed rare copy number variants implicate novel genes in schizophrenia. Biochem. Soc. Trans..

[17] Issler O., van der Zee Y.Y., Ramakrishnan A., Wang J., Tan C., Loh Y.E. (2020). Sex-specific role for the long non-coding RNA LINC00473 in depression. Neuron.

[18] Gandal M.J., Zhang P., Hadjimichael E., Walker R.L., Chen C., Liu S. (2018). Transcriptome-wide isoform-level dysregulation in ASD, schizophrenia, and bipolar disorder. Science.

[19] Chen Z., Li J.L., Lin S., Cao C., Gimbrone N.T., Yang R. (2016). cAMP/CREB-regulated LINC00473 marks LKB1-inactivated lung cancer and mediates tumor growth. J. Clin. Invest..

[20] Tran K.V., Brown E.L., DeSouza T., Jespersen N.Z., Nandrup-Bus C., Yang Q. (2020). Human thermogenic adipocyte regulation by the long noncoding RNA LINC00473. Nat. Metab..

[21] Mas-Ponte D., Carlevaro-Fita J., Palumbo E., Hermoso Pulido T., Guigo R., Johnson R. (2017). LncATLAS database for subcellular localization of long noncoding RNAs. RNA.

[22] Shi C., Yang Y., Yu J., Meng F., Zhang T., Gao Y. (2017). The long noncoding RNA LINC00473, a target of microRNA 34a, promotes tumorigenesis by inhibiting ILF2 degradation in cervical cancer. Am. J. Cancer Res..

[23] Bavamian S., Mellios N., Lalonde J., Fass D.M., Wang J., Sheridan S.D. (2015). Dysregulation of miR-34a links neuronal development to genetic risk factors for bipolar disorder. Mol. Psychiatry.

[24] GTEx Consortium (2013). The genotype-tissue expression (GTEx) project. Nat. Genet..

[25] Reitmair A., Sachs G., Im W.B., Wheeler L. (2012). C6orf176: a novel possible regulator of cAMP-mediated gene expression. Physiol. Genomics.

[26] Hawrylycz M.J., Lein E.S., Guillozet-Bongaarts A.L., Shen E.H., Ng L., Miller J.A. (2012). An anatomically comprehensive atlas of the adult human brain transcriptome. Nature.

[27] Miller J.A., Ding S.L., Sunkin S.M., Smith K.A., Ng L., Szafer A. (2014). Transcriptional landscape of the prenatal human brain. Nature.

[28] Dinh T.A., Vitucci E.C., Wauthier E., Graham R.P., Pitman W.A., Oikawa T. (2017). Comprehensive analysis of the Cancer Genome Atlas reveals a unique gene and non-coding RNA signature of fibrolamellar carcinoma. Sci. Rep..

[29] Zhang W., Song Y. (2018). LINC00473 predicts poor prognosis and regulates cell migration and invasion in gastric cancer. Biomed. Pharmacother..

[30] Chen H., Yang F., Li X., Gong Z.J., Wang L.W. (2018). Long noncoding RNA LNC473 inhibits the ubiquitination of survivin via association with USP9X and enhances cell proliferation and invasion in hepatocellular carcinoma cells. Biochem. Biophys. Res. Commun..

[31] Han P.B., Ji X.J., Zhang M., Gao L.Y. (2018). Upregulation of lncRNA LINC00473 promotes radioresistance of HNSCC cells through activating Wnt/beta-catenin signaling pathway. Eur. Rev. Med. Pharmacol. Sci..

[32] Duman R.S., Heninger G.R., Nestler E.J. (1997). A molecular and cellular theory of depression. Arch. Gen. Psychiatry.

[33] Ren X., Rizavi H.S., Khan M.A., Bhaumik R., Dwivedi Y., Pandey G.N. (2014). Alteration of cyclic-AMP response element binding protein in the postmortem brain of subjects with bipolar disorder and schizophrenia. J. Affect Disord..

[34] Wang H., Xu J., Lazarovici P., Quirion R., Zheng W. (2018). cAMP response element-binding protein (CREB): a possible signaling molecule link in the pathophysiology of schizophrenia. Front. Mol. Neurosci..

[35] Herrmann C.J., Schmidt R., Kanitz A., Artimo P., Gruber A.J., Zavolan M. (2020). PolyASite 2.0: a consolidated atlas of polyadenylation sites from 3' end sequencing. Nucleic Acids Res..

[36] Thierry-Mieg D., Thierry-Mieg J. (2006). AceView: a comprehensive cDNA-supported gene and transcripts annotation. Genome Biol..

[37] Reitmair A., Lambrecht N.W., Yakubov I., Nieves A., Old D., Donde Y. (2010). Prostaglandin E2 receptor subtype EP2- and EP4-regulated gene expression profiling in human ciliary smooth muscle cells. Physiol. Genomics.

[38] Duffy E.E., Finander B., Choi G., Carter A.C., Pritisanac I., Alam A. (2022). Developmental dynamics of RNA translation in the human brain. Nat. Neurosci..

[39] Schmitz J.F., Ullrich K.K., Bornberg-Bauer E. (2018). Incipient de novo genes can evolve from frozen accidents that escaped rapid transcript turnover. Nat. Ecol. Evol..

[40] Lou H., Kim S.K., Zaitsev E., Snell C.R., Lu B., Loh Y.P. (2005). Sorting and activity-dependent secretion of BDNF require interaction of a specific motif with the sorting receptor carboxypeptidase e. Neuron.

[41] Parrini E., Ramazzotti A., Dobyns W.B., Mei D., Moro F., Veggiotti P. (2006). Periventricular heterotopia: phenotypic heterogeneity and correlation with filamin A mutations. Brain.

[42] Muona M., Berkovic S.F., Dibbens L.M., Oliver K.L., Maljevic S., Bayly M.A. (2015). A recurrent de novo mutation in KCNC1 causes progressive myoclonus epilepsy. Nat. Genet..

[43] Reif A., Herterich S., Strobel A., Ehlis A.C., Saur D., Jacob C.P. (2006). A neuronal nitric oxide synthase (NOS-I) haplotype associated with schizophrenia modifies prefrontal cortex function. Mol. Psychiatry.

[44] Zaki M.S., Accogli A., Mirzaa G., Rahman F., Mohammed H., Porras-Hurtado G.L. (2021). Pathogenic variants in PIDD1 lead to an autosomal recessive neurodevelopmental disorder with pachygyria and psychiatric features. Eur. J. Hum. Genet..

[45] Mignot C., von Stulpnagel C., Nava C., Ville D., Sanlaville D., Lesca G. (2016). Genetic and neurodevelopmental spectrum of SYNGAP1-associated intellectual disability and epilepsy. J. Med. Genet..

[46] De Roeck A., Van Broeckhoven C., Sleegers K. (2019). The role of ABCA7 in Alzheimer's disease: evidence from genomics, transcriptomics and methylomics. Acta Neuropathol..

[47] Wang H.Y., Bakshi K., Frankfurt M., Stucky A., Goberdhan M., Shah S.M. (2012). Reducing amyloid-related Alzheimer's disease pathogenesis by a small molecule targeting filamin A. J. Neurosci..

[48] Galimberti D., Scarpini E., Venturelli E., Strobel A., Herterich S., Fenoglio C. (2008). Association of a NOS1 promoter repeat with Alzheimer's disease. Neurobiol. Aging.

[49] Teerlink C.C., Miller J.B., Vance E.L., Staley L.A., Stevens J., Tavana J.P. (2022). Analysis of high-risk pedigrees identifies 11 candidate variants for Alzheimer's disease. Alzheimers Dement..

[50] Torkamani A., Bersell K., Jorge B.S., Bjork R.L., Friedman J.R., Bloss C.S. (2014). De novo KCNB1 mutations in epileptic encephalopathy. Ann. Neurol..

[51] Nappi P., Miceli F., Soldovieri M.V., Ambrosino P., Barrese V., Taglialatela M. (2020). Epileptic channelopathies caused by neuronal Kv7 (KCNQ) channel dysfunction. Pflugers Arch..

[52] Lipton J.O., Sahin M. (2014). The neurology of mTOR. Neuron.

[53] Takei N., Inamura N., Kawamura M., Namba H., Hara K., Yonezawa K. (2004). Brain-derived neurotrophic factor induces mammalian target of rapamycin-dependent local activation of translation machinery and protein synthesis in neuronal dendrites. J. Neurosci..

[54] Oyrer J., Maljevic S., Scheffer I.E., Berkovic S.F., Petrou S., Reid C.A. (2018). Ion channels in genetic epilepsy: from genes and mechanisms to disease-targeted therapies. Pharmacol. Rev..

[55] Dong Y., Green T., Saal D., Marie H., Neve R., Nestler E.J. (2006). CREB modulates excitability of nucleus accumbens neurons. Nat. Neurosci..

[56] Lopez de Armentia M., Jancic D., Olivares R., Alarcon J.M., Kandel E.R., Barco A. (2007). cAMP response element-binding protein-mediated gene expression increases the intrinsic excitability of CA1 pyramidal neurons. J. Neurosci..

[57] Shaywitz A.J., Greenberg M.E. (1999). CREB: a stimulus-induced transcription factor activated by a diverse array of extracellular signals. Annu. Rev. Biochem..

[58] Ginty D.D., Kornhauser J.M., Thompson M.A., Bading H., Mayo K.E., Takahashi J.S. (1993). Regulation of CREB phosphorylation in the suprachiasmatic nucleus by light and a circadian clock. Science.

[59] Conkright M.D., Canettieri G., Screaton R., Guzman E., Miraglia L., Hogenesch J.B. (2003). TORCs: transducers of regulated CREB activity. Mol. Cell.

[60] Hagenston A.M., Bading H. (2011). Calcium signaling in synapse-to-nucleus communication. Cold Spring Harb. Perspect. Biol..

[61] Lisman J., Cooper K., Sehgal M., Silva A.J. (2018). Memory formation depends on both synapse-specific modifications of synaptic strength and cell-specific increases in excitability. Nat. Neurosci..

[62] Penn Y., Segal M., Moses E. (2016). Network synchronization in hippocampal neurons. Proc. Natl. Acad. Sci. U. S. A..

[63] Benito E., Valor L.M., Jimenez-Minchan M., Huber W., Barco A. (2011). cAMP response element-binding protein is a primary hub of activity-driven neuronal gene expression. J. Neurosci..

[64] Li S., Zhang C., Takemori H., Zhou Y., Xiong Z.Q. (2009). TORC1 regulates activity-dependent CREB-target gene transcription and dendritic growth of developing cortical neurons. J. Neurosci..

[65] Finsterwald C., Fiumelli H., Cardinaux J.R., Martin J.L. (2010). Regulation of dendritic development by BDNF requires activation of CRTC1 by glutamate. J. Biol. Chem..

[66] Poolos N.P., Migliore M., Johnston D. (2002). Pharmacological upregulation of h-channels reduces the excitability of pyramidal neuron dendrites. Nat. Neurosci..

[67] Frick A., Magee J., Johnston D. (2004). LTP is accompanied by an enhanced local excitability of pyramidal neuron dendrites. Nat. Neurosci..

[68] Day M., Carr D.B., Ulrich S., Ilijic E., Tkatch T., Surmeier D.J. (2005). Dendritic excitability of mouse frontal cortex pyramidal neurons is shaped by the interaction among HCN, Kir2, and Kleak channels. J. Neurosci..

[69] Kase D., Imoto K. (2012). The role of HCN channels on membrane excitability in the nervous system. J. Signal Transduct..

[70] Kim J., Wei D.S., Hoffman D.A. (2005). Kv4 potassium channel subunits control action potential repolarization and frequency-dependent broadening in rat hippocampal CA1 pyramidal neurones. J. Physiol..

[71] Simkin D., Hattori S., Ybarra N., Musial T.F., Buss E.W., Richter H. (2015). Aging-related hyperexcitability in CA3 pyramidal neurons is mediated by enhanced A-type K+ channel function and expression. J. Neurosci..

[72] Chujo T., Yamazaki T., Kawaguchi T., Kurosaka S., Takumi T., Nakagawa S. (2017). Unusual semi-extractability as a hallmark of nuclear body-associated architectural noncoding RNAs. EMBO J..

[73] Shav-Tal Y., Zipori D. (2002). PSF and p54(nrb)/NonO--multi-functional nuclear proteins. FEBS Lett..

[74] Redmond L., Kashani A.H., Ghosh A. (2002). Calcium regulation of dendritic growth via CaM kinase IV and CREB-mediated transcription. Neuron.

[75] Shieh P.B., Hu S.C., Bobb K., Timmusk T., Ghosh A. (1998). Identification of a signaling pathway involved in calcium regulation of BDNF expression. Neuron.

[76] Tao X., Finkbeiner S., Arnold D.B., Shaywitz A.J., Greenberg M.E. (1998). Ca2+ influx regulates BDNF transcription by a CREB family transcription factor-dependent mechanism. Neuron.

[77] Liu Z., Chen X., Wang Y., Peng H., Wang Y., Jing Y. (2014). PDK4 protein promotes tumorigenesis through activation of cAMP-response element-binding protein (CREB)-Ras homolog enriched in brain (RHEB)-mTORC1 signaling cascade. J. Biol. Chem..

[78] Kokaia M., Ernfors P., Kokaia Z., Elmer E., Jaenisch R., Lindvall O. (1995). Suppressed epileptogenesis in BDNF mutant mice. Exp. Neurol..

[79] Croll S.D., Suri C., Compton D.L., Simmons M.V., Yancopoulos G.D., Lindsay R.M. (1999). Brain-derived neurotrophic factor transgenic mice exhibit passive avoidance deficits, increased seizure severity and *in vitro* hyperexcitability in the hippocampus and entorhinal cortex. Neuroscience.

[80] Proietti Onori M., Koene L.M.C., Schafer C.B., Nellist M., de Brito van Velze M., Gao Z. (2021). RHEB/mTOR hyperactivity causes cortical malformations and epileptic seizures through increased axonal connectivity. PLoS Biol..

[81] Bonni A., Ginty D.D., Dudek H., Greenberg M.E. (1995). Serine 133-phosphorylated CREB induces transcription via a cooperative mechanism that may confer specificity to neurotrophin signals. Mol. Cell. Neurosci..

[82] Esvald E.E., Tuvikene J., Sirp A., Patil S., Bramham C.R., Timmusk T. (2020). CREB family transcription factors are major mediators of BDNF transcriptional autoregulation in cortical neurons. J. Neurosci..

[83] Nam J.H., Leem E., Jeon M.T., Jeong K.H., Park J.W., Jung U.J. (2015). Induction of GDNF and BDNF by hRheb(S16H) transduction of SNpc neurons: neuroprotective mechanisms of hRheb(S16H) in a model of Parkinson's disease. Mol. Neurobiol..

[84] Damaj L., Lupien-Meilleur A., Lortie A., Riou E., Ospina L.H., Gagnon L. (2015). CACNA1A haploinsufficiency causes cognitive impairment, autism and epileptic encephalopathy with mild cerebellar symptoms. Eur. J. Hum. Genet..

[85] Reinson K., Oiglane-Shlik E., Talvik I., Vaher U., Ounapuu A., Ennok M. (2016). Biallelic CACNA1A mutations cause early onset epileptic encephalopathy with progressive cerebral, cerebellar, and optic nerve atrophy. Am. J. Med. Genet. A.

[86] von Spiczak S., Helbig K.L., Shinde D.N., Huether R., Pendziwiat M., Lourenco C. (2017). DNM1 encephalopathy: a new disease of vesicle fission. Neurology.

[87] Samanta D. (2020). PCDH19-related epilepsy syndrome: a comprehensive clinical review. Pediatr. Neurol..

[88] Han J.H., Kushner S.A., Yiu A.P., Cole C.J., Matynia A., Brown R.A. (2007). Neuronal competition and selection during memory formation. Science.

[89] Yiu A.P., Mercaldo V., Yan C., Richards B., Rashid A.J., Hsiang H.L. (2014). Neurons are recruited to a memory trace based on relative neuronal excitability immediately before training. Neuron.

[90] Chen L., Cummings K.A., Mau W., Zaki Y., Dong Z., Rabinowitz S. (2020). The role of intrinsic excitability in the evolution of memory: significance in memory allocation, consolidation, and updating. Neurobiol. Learn Mem..

[91] Hamm J.P., Peterka D.S., Gogos J.A., Yuste R. (2017). Altered cortical ensembles in mouse models of schizophrenia. Neuron.

[92] Winterer G., Ziller M., Dorn H., Frick K., Mulert C., Wuebben Y. (2000). Schizophrenia: reduced signal-to-noise ratio and impaired phase-locking during information processing. Clin. Neurophysiol..

[93] Winterer G., Coppola R., Goldberg T.E., Egan M.F., Jones D.W., Sanchez C.E. (2004). Prefrontal broadband noise, working memory, and genetic risk for schizophrenia. Am. J. Psychiatry.

[94] Winterer G. (2006). Cortical microcircuits in schizophrenia--the dopamine hypothesis revisited. Pharmacopsychiatry.

[95] Rolls E.T., Loh M., Deco G., Winterer G. (2008). Computational models of schizophrenia and dopamine modulation in the prefrontal cortex. Nat. Rev. Neurosci..

[96] Kuttner R.E., Lorincz A.B., Swan D.A. (1967). The schizophrenia gene and social evolution. Psychol. Rep..

[97] Randall P.L. (1983). Schizophrenia, abnormal connection, and brain evolution. Med. Hypotheses.

[98] Crow T.J. (1997). Is schizophrenia the price that Homo sapiens pays for language?. Schizophr. Res..

[99] Schmidt E.R.E., Polleux F. (2021). Genetic mechanisms underlying the evolution of connectivity in the human cortex. Front. Neural Circuits.

[100] Langmead B., Salzberg S.L. (2012). Fast gapped-read alignment with Bowtie 2. Nat. Methods.

[101] Blankenberg D., Gordon A., Von Kuster G., Coraor N., Taylor J., Nekrutenko A. (2010). Manipulation of FASTQ data with Galaxy. Bioinformatics.

[102] Kim D., Langmead B., Salzberg S.L. (2015). HISAT: a fast spliced aligner with low memory requirements. Nat. Methods.

[103] Anders S., Reyes A., Huber W. (2012). Detecting differential usage of exons from RNA-seq data. Genome Res..

[104] Goecks J., Nekrutenko A., Taylor J., Galaxy T. (2010). Galaxy: a comprehensive approach for supporting accessible, reproducible, and transparent computational research in the life sciences. Genome Biol..

[105] Anders S., Pyl P.T., Huber W. (2015). HTSeq--a Python framework to work with high-throughput sequencing data. Bioinformatics.

[106] Love M.I., Huber W., Anders S. (2014). Moderated estimation of fold change and dispersion for RNA-seq data with DESeq2. Genome Biol..

[107] Young M.D., Wakefield M.J., Smyth G.K., Oshlack A. (2010). Gene ontology analysis for RNA-seq: accounting for selection bias. Genome Biol..

[108] Raudvere U., Kolberg L., Kuzmin I., Arak T., Adler P., Peterson H. (2019). g:Profiler: a web server for functional enrichment analysis and conversions of gene lists (2019 update). Nucleic Acids Res..

[109] Kwon A.T., Arenillas D.J., Worsley Hunt R., Wasserman W.W. (2012). oPOSSUM-3: advanced analysis of regulatory motif over-representation across genes or ChIP-Seq datasets. G3 (Bethesda).

[110] Portales-Casamar E., Arenillas D., Lim J., Swanson M.I., Jiang S., McCallum A. (2009). The PAZAR database of gene regulatory information coupled to the ORCA toolkit for the study of regulatory sequences. Nucleic Acids Res..

[111] Corvelo A., Hallegger M., Smith C.W., Eyras E. (2010). Genome-wide association between branch point properties and alternative splicing. PLoS Comput. Biol..

[112] Liu H., Han H., Li J., Wong L. (2003). An in-silico method for prediction of polyadenylation signals in human sequences. Genome Inform..

[113] Madeira F., Park Y.M., Lee J., Buso N., Gur T., Madhusoodanan N. (2019). The EMBL-EBI search and sequence analysis tools APIs in 2019. Nucleic Acids Res..

[114] Letunic I., Bork P. (2021). Interactive Tree Of Life (iTOL) v5: an online tool for phylogenetic tree display and annotation. Nucleic Acids Res..

[115] McClure C., Cole K.L., Wulff P., Klugmann M., Murray A.J. (2011). Production and titering of recombinant adeno-associated viral vectors. J. Vis. Exp..

[116] Impey S., McCorkle S.R., Cha-Molstad H., Dwyer J.M., Yochum G.S., Boss J.M. (2004). Defining the CREB regulon: a genome-wide analysis of transcription factor regulatory regions. Cell.

[117] Zhang X., Odom D.T., Koo S.H., Conkright M.D., Canettieri G., Best J. (2005). Genome-wide analysis of cAMP-response element binding protein occupancy, phosphorylation, and target gene activation in human tissues. Proc. Natl. Acad. Sci. U. S. A..

[118] Xu W., Kasper L.H., Lerach S., Jeevan T., Brindle P.K. (2007). Individual CREB-target genes dictate usage of distinct cAMP-responsive coactivation mechanisms. EMBO J..

[119] Pardo L., Valor L.M., Eraso-Pichot A., Barco A., Golbano A., Hardingham G.E. (2017). CREB regulates distinct adaptive transcriptional programs in astrocytes and neurons. Sci. Rep..

[120] Carvill G.L., Regan B.M., Yendle S.C., O'Roak B.J., Lozovaya N., Bruneau N. (2013). GRIN2A mutations cause epilepsy-aphasia spectrum disorders. Nat. Genet..

[121] Bailey C.S., Moldenhauer H.J., Park S.M., Keros S., Meredith A.L. (2019). KCNMA1-linked channelopathy. J. Gen. Physiol..

[122] Bonardi C.M., Heyne H.O., Fiannacca M., Fitzgerald M.P., Gardella E., Gunning B. (2021). KCNT1-related epilepsies and epileptic encephalopathies: phenotypic and mutational spectrum. Brain.

[123] Parihar R., Ganesh S. (2013). The SCN1A gene variants and epileptic encephalopathies. J. Hum. Genet..

[124] Puskarjov M., Seja P., Heron S.E., Williams T.C., Ahmad F., Iona X. (2014). A variant of KCC2 from patients with febrile seizures impairs neuronal Cl- extrusion and dendritic spine formation. EMBO Rep..

[125] Basel-Vanagaite L., Hershkovitz T., Heyman E., Raspall-Chaure M., Kakar N., Smirin-Yosef P. (2013). Biallelic SZT2 mutations cause infantile encephalopathy with epilepsy and dysmorphic corpus callosum. Am. J. Hum. Genet..

[126] Balestrini S., Milh M., Castiglioni C., Luthy K., Finelli M.J., Verstreken P. (2016). TBC1D24 genotype-phenotype correlation: epilepsies and other neurologic features. Neurology.

[127] Wang J., Lin Z.J., Liu L., Xu H.Q., Shi Y.W., Yi Y.H. (2017). Epilepsy-associated genes. Seizure.

[128] Calhoun J.D., Huffman A.M., Bellinski I., Kinsley L., Bachman E., Gerard E. (2020). CACNA1H variants are not a cause of monogenic epilepsy. Hum. Mutat..

