# Supplementary material for: Expression of the primate-specific LINC00473 RNA in mouse neurons promotes excitability and CREB-regulated transcription
Source: J Biol Chem. 2023 Apr 3;299(5):104671. doi: 10.1016/j.jbc.2023.104671 (PMC10235438; doi:10.1016/j.jbc.2023.104671)
Supplement: Supplemental Tables S1–S14 and Figures S1–S4 [file mmc1.pdf]

Supporting information

Expression of the primate-specific *LINC00473* RNA in mouse neurons promotes excitability and CREB-regulated transcription

Priit Pruunsild, C. Peter Bengtson, Isabel Loss, Benjamin Lohrer and Hilmar Bading

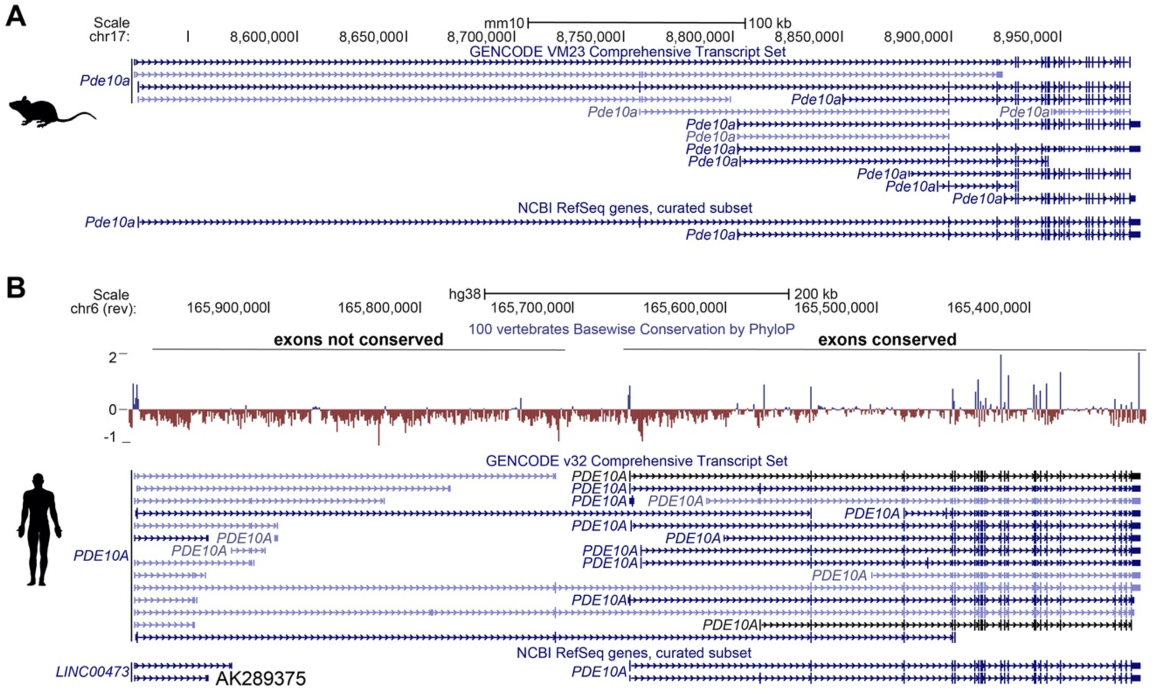

Figure S1. Comparison of the mouse *Pde10a* and human *PDE10A* gene loci. Mouse (*A*) or human (*B*) transcripts from GENCODE and NCBI RNA reference sequences collection (RefSeq) databases illustrate the exon-intron structures of the gene orthologs visualized with the UCSC genome browser. The comprehensive GENCODE transcript set tracks visualize data labelled with gene symbols as follows: black, feature has a corresponding entry in the Protein Data Bank (PDB); dark blue, transcript has been reviewed or validated by either the RefSeq or SwissProt staff; medium blue, other RefSeq transcripts; light blue, non-RefSeq transcripts. The NCBI curated RefSeq genes subtrack visualizes data labelled with gene symbols as follows: dark blue, reviewed - the RefSeq record has been reviewed by NCBI staff or by a collaborator; the NCBI review process includes assessing available sequence data and the literature. *B*, Vertebrate basewise conservation track is based on multiple alignments generated using multiz and other tools in the UCSC genomics alignment pipeline. The PhyloP program produces separate scores at each base, considering all branches of the phylogeny. Conserved sites are assigned positive scores (blue), fast-evolving sites are assigned negative scores (reddish brown). Note that in the NCBI RefSeq database *LINC00473* (*Lnc473*) occurs as a separate gene. AK289375 is the GenBank accession number of the transcript variant that contains the exons found in this study to be used in the spliced synaptic activity-regulated *Lnc473* RNA. The human sequence has been inverted.

Table S1. Human *PDE10A* exon usage after 1 h action potential firing.

TSS	3'	Gene:exon	log_2FC	BaseMean	*p* value	p_{Bonf}	Chr	Start	End
**+**	**-**	**ENSG00000112541:E1**	**1.70**	**10.71**	**2.07E-02**	**1**	**6**	**165987529**	**165988078**
+	-	ENSG00000112541:E2	0.62	2.88	0.31	1	6	165986108	165986603
-	-	ENSG00000112541:E3	30.39	0.37	0.13	1	6	165949620	165949914
-	-	ENSG00000112541:E4	2.47	0.90	0.06	1	6	165949255	165949619
-	-	ENSG00000112541:E5	2.35	1.66	1.51E-02	0.74	6	165948609	165949254
-	(+)	ENSG00000112541:E6	-0.10	0.50	0.99	1	6	165948464	165948608
-	-	ENSG00000112541:E7	0.69	1.03	0.75	1	6	165948223	165948463
-	+	ENSG00000112541:E8	1.47	0.97	0.19	1	6	165946772	165947220
**-**	**+**	**ENSG00000112541:E9**	**2.81**	**27.56**	**7.17E-13**	**3.51E-11**	**6**	**165939456**	**165940766**
-	-	ENSG00000112541:E10	1.09	3.98	4.36E-02	1	6	165924048	165924592
-	-	ENSG00000112541:E11	30.39	0.34	0.13	1	6	165910808	165910932
-	+	ENSG00000112541:E12	2.21	0.76	0.12	1	6	165908802	165909080
-	+	ENSG00000112541:E13	0.27	1.61	0.83	1	6	165901820	165902864
-	+	ENSG00000112541:E14	0.15	5.24	0.71	1	6	165780269	165781923
+	-	ENSG00000112541:E15	-0.27	17.63	0.62	1	6	165662555	165663245
+	-	ENSG00000112541:E16	-0.32	20.14	0.59	1	6	165662101	165662554
+	-	ENSG00000112541:E17	-0.28	12.61	0.77	1	6	165661947	165662100
+	-	ENSG00000112541:E18	0.48	0.63	0.72	1	6	165661074	165661578
+	-	ENSG00000112541:E19	0.46	0.63	0.73	1	6	165660955	165661073
+	-	ENSG00000112541:E20	-0.10	2.19	0.40	1	6	165660069	165660954
+	-	ENSG00000112541:E21	-0.62	3.54	0.29	1	6	165654813	165655336
+	-	ENSG00000112541:E22	-1.11	0.33	0.61	1	6	165611354	165611496
+	-	ENSG00000112541:E23	-30.71	0.33	0.10	1	6	165600166	165600778
(+)	-	ENSG00000112541:E24	-0.09	0.96	0.91	1	6	165576412	165576539
-	(+)	ENSG00000112541:E25	0.36	15.40	0.23	1	6	165543440	165543568
+	-	ENSG00000112541:E26	-0.12	0.30	0.96	1	6	165503005	165503356
(+)	-	ENSG00000112541:E27	0.39	7.05	0.39	1	6	165482315	165482343
-	-	ENSG00000112541:E28	29.81	0.25	0.28	1	6	165466420	165466532
-	-	ENSG00000112541:E29	-29.13	0.11	0.53	1	6	165454578	165454695
-	-	ENSG00000112541:E30	0.23	24.12	0.50	1	6	165450242	165450362
-	-	ENSG00000112541:E31	-0.05	14.40	1	1	6	165448928	165448977
-	-	ENSG00000112541:E32	-0.34	29.59	0.52	1	6	165435237	165435377
-	-	ENSG00000112541:E33	-0.46	29.05	0.29	1	6	165432974	165433129
-	-	ENSG00000112541:E34	-0.69	10.81	0.22	1	6	165431422	165431472
-	-	ENSG00000112541:E35	-0.25	10.76	0.77	1	6	165430287	165430345
-	-	ENSG00000112541:E36	0.01	6.49	0.89	1	6	165428658	165428709
-	-	ENSG00000112541:E37	-0.04	16.30	0.95	1	6	165418635	165418777
-	-	ENSG00000112541:E38	-0.40	22.53	0.21	1	6	165416189	165416281
-	-	ENSG00000112541:E39	-0.38	39.17	0.32	1	6	165413501	165413687
-	-	ENSG00000112541:E40	-0.27	29.67	0.39	1	6	165396317	165396459
-	-	ENSG00000112541:E41	-0.34	22.80	0.37	1	6	165395181	165395264
-	-	ENSG00000112541:E42	-0.13	31.50	0.71	1	6	165392646	165392796
-	-	ENSG00000112541:E43	-0.31	29.21	0.54	1	6	165388298	165388453
-	-	ENSG00000112541:E44	-0.42	34.63	0.24	1	6	165379194	165379366
-	-	ENSG00000112541:E45	0.15	21.25	0.59	1	6	165343391	165343502
-	-	ENSG00000112541:E46	-0.22	19.33	0.63	1	6	165339278	165339358
-	-	ENSG00000112541:E47	-0.42	27.36	0.32	1	6	165336123	165336211
-	+	ENSG00000112541:E48	-0.06	288.53	0.79	1	6	165331050	165333127
-	+	ENSG00000112541:E49	0.00	775.77	0.96	1	6	165327287	165331049

Genome-wide differential exon usage in response to action potential (AP) firing in co-cultures of human iPSC-derived neurons and mouse primary hippocampal neurons (1) was determined with the DEXSeq package (103). Relative usage levels of the individual *PDE10A* (ENSG00000112541) exons after 1 h of AP firing (indicated by log_2 fold change relative to control condition, log_2FC) were calculated. TSS, transcriptional start site; + indicates that the exon is exclusively used as a 5' exon; (+) specifies a possible transcript 5' end. 3', + indicates that the exon is a 3' exon; (+) specifies possible transcription termination. p_{Bonf} shows Bonferroni-corrected *p* values for the *PDE10A* exons. Genomic locations refer to hg38. n = 4 for both control condition and 1 h AP firing.

Table S2. Human *PDE10A* exon usage after 4 h action potential firing.

TSS	3’	Gene:exon	log_2FC	BaseMean	*p* value	p_{Bonf}	Chr	Start	End
+	**-**	**ENSG00000112541:E1**	**3.60**	**47.37**	**4.70E-16**	**2.30E-14**	**6**	**165987529**	**165988078**
+	-	ENSG00000112541:E2	0.35	3.45	0.61	1	6	165986108	165986603
-	-	ENSG00000112541:E3	28.97	0.63	0.05	1	6	165949620	165949914
-	-	ENSG00000112541:E4	3.36	2.08	1.06E-03	0.05	6	165949255	165949619
-	-	ENSG00000112541:E5	3.20	3.83	4.56E-03	0.22	6	165948609	165949254
-	(+)	ENSG00000112541:E6	0.37	0.77	0.72	1	6	165948464	165948608
-	-	ENSG00000112541:E7	-0.62	0.84	0.73	1	6	165948223	165948463
-	+	ENSG00000112541:E8	1.14	1.07	0.39	1	6	165946772	165947220
-	**+**	**ENSG00000112541:E9**	**3.80**	**72.18**	**3.95E-12**	**1.94E-10**	**6**	**165939456**	**165940766**
-	-	ENSG00000112541:E10	3.50	23.22	1.10E-22	5.39E-21	6	165924048	165924592
-	-	ENSG00000112541:E11	29.24	0.78	2.96E-02	1	6	165910808	165910932
-	+	ENSG00000112541:E12	3.82	2.86	7.36E-05	3.61E-03	6	165908802	165909080
-	+	ENSG00000112541:E13	2.07	5.77	6.37E-05	3.12E-03	6	165901820	165902864
-	+	ENSG00000112541:E14	2.80	28.97	1.60E-26	7.84E-25	6	165780269	165781923
+	-	ENSG00000112541:E15	-1.31	17.50	0.09	1	6	165662555	165663245
+	-	ENSG00000112541:E16	-0.90	22.65	0.15	1	6	165662101	165662554
+	-	ENSG00000112541:E17	-0.47	15.81	0.79	1	6	165661947	165662100
+	-	ENSG00000112541:E18	-0.63	0.55	0.76	1	6	165661074	165661578
+	-	ENSG00000112541:E19	-1.64	0.49	0.41	1	6	165660955	165661073
+	-	ENSG00000112541:E20	0.00	3.10	0.93	1	6	165660069	165660954
+	-	ENSG00000112541:E21	-0.92	4.33	0.17	1	6	165654813	165655336
+	-	ENSG00000112541:E22	-0.06	0.62	1	1	6	165611354	165611496
+	-	ENSG00000112541:E23	-1.22	0.65	0.45	1	6	165600166	165600778
(+)	-	ENSG00000112541:E24	1.06	2.11	0.13	1	6	165576412	165576539
-	(+)	ENSG00000112541:E25	0.31	20.32	0.14	1	6	165543440	165543568
+	-	ENSG00000112541:E26	2.16	1.02	0.08	1	6	165503005	165503356
(+)	-	ENSG00000112541:E27	-0.16	7.79	0.88	1	6	165482315	165482343
-	-	ENSG00000112541:E28	26.63	0.11	0.87	1	6	165466420	165466532
-	-	ENSG00000112541:E29	-27.98	0.14	0.35	1	6	165454578	165454695
-	-	ENSG00000112541:E30	-0.39	25.94	0.79	1	6	165450242	165450362
-	-	ENSG00000112541:E31	-0.55	16.33	0.43	1	6	165448928	165448977
-	-	ENSG00000112541:E32	-0.80	34.39	0.17	1	6	165435237	165435377
-	-	ENSG00000112541:E33	-0.78	35.36	0.21	1	6	165432974	165433129
-	-	ENSG00000112541:E34	-0.47	15.40	0.65	1	6	165431422	165431472
-	-	ENSG00000112541:E35	-0.12	15.17	0.79	1	6	165430287	165430345
-	-	ENSG00000112541:E36	-0.04	8.55	0.71	1	6	165428658	165428709
-	-	ENSG00000112541:E37	-0.13	21.09	0.80	1	6	165418635	165418777
-	-	ENSG00000112541:E38	-0.72	27.41	0.08	1	6	165416189	165416281
-	-	ENSG00000112541:E39	-0.91	44.77	0.10	1	6	165413501	165413687
-	-	ENSG00000112541:E40	-0.69	34.83	0.05	1	6	165396317	165396459
-	-	ENSG00000112541:E41	-0.52	28.88	0.22	1	6	165395181	165395264
-	-	ENSG00000112541:E42	-0.74	34.61	0.11	1	6	165392646	165392796
-	-	ENSG00000112541:E43	-0.65	35.01	0.44	1	6	165388298	165388453
-	-	ENSG00000112541:E44	-0.61	43.79	0.21	1	6	165379194	165379366
-	-	ENSG00000112541:E45	-0.31	24.04	0.58	1	6	165343391	165343502
-	-	ENSG00000112541:E46	-0.30	25.26	0.82	1	6	165339278	165339358
-	-	ENSG00000112541:E47	-0.63	34.33	0.16	1	6	165336123	165336211
-	+	ENSG00000112541:E48	-0.40	344.71	0.14	1	6	165331050	165333127
-	+	ENSG00000112541:E49	-0.35	924.04	0.49	1	6	165327287	165331049

Same as Table S1, except that relative usage levels of the individual *PDE10A* (ENSG00000112541) exons after 4 h of AP firing were calculated. TSS, transcriptional start site; + indicates that the exon is exclusively used as a 5’ exon; (+) specifies a possible transcript 5’ end. 3’, + indicates that the exon is a 3’ exon; (+) specifies possible transcription termination. p_{Bonf} shows Bonferroni-corrected *p* values for the *PDE10A* exons. n = 4 for control condition, n = 3 for 4 h AP firing.

Table S3. Mouse *Pde10a* exon usage after 1 h action potential firing.

TSS	3’	Gene:exon	log_2FC	BaseMean	*p* value	p_{Bonf}	Chr	Start	End
+	-	ENSMUSG00000023868:E001	-0.65	1.03	0.56	1	17	8525372	8525449
+	-	ENSMUSG00000023868:E002	0.71	4.73	0.16	1	17	8525450	8525815
+	-	ENSMUSG00000023868:E003	1.41	1.25	0.33	1	17	8526801	8527025
+	-	ENSMUSG00000023868:E004	-0.26	0.89	0.78	1	17	8527026	8527115
(+)	-	ENSMUSG00000023868:E005	-0.01	2.60	0.95	1	17	8756962	8757017
-	-	ENSMUSG00000023868:E006	-0.28	3.51	0.96	1	17	8757018	8757093
-	(+)	ENSMUSG00000023868:E007	-0.28	0.97	0.76	1	17	8758145	8758267
(+)	-	ENSMUSG00000023868:E008	NA	0.00	NA	NA	17	8798579	8798731
(+)	-	ENSMUSG00000023868:E009	0.52	2.59	0.56	1	17	8801693	8801778
-	-	ENSMUSG00000023868:E010	0.80	14.01	0.07	1	17	8801779	8802145
(+)	-	ENSMUSG00000023868:E011	1.92	0.81	0.18	1	17	8803028	8803122
(+)	-	ENSMUSG00000023868:E012	NA	0.00	NA	NA	17	8849985	8850244
(+)	-	ENSMUSG00000023868:E013	-10.97	0.12	0.63	1	17	8880426	8880479
(+)	-	ENSMUSG00000023868:E014	-10.97	0.12	0.63	1	17	8893291	8893449
(+)	-	ENSMUSG00000023868:E015	-0.08	20.96	0.71	1	17	8898861	8898989
(+)	-	ENSMUSG00000023868:E016	1.39	0.90	0.25	1	17	8898990	8899189
-	-	ENSMUSG00000023868:E017	0.05	0.46	1	1	17	8899190	8899193
-	-	ENSMUSG00000023868:E018	-0.06	13.82	0.79	1	17	8920504	8920532
-	(+)	ENSMUSG00000023868:E019	-1.77	1.51	0.25	1	17	8920533	8923426
(+)	-	ENSMUSG00000023868:E020	10.43	0.19	0.50	1	17	8924143	8924241
-	-	ENSMUSG00000023868:E021	-0.21	22.76	0.28	1	17	8929092	8929212
-	-	ENSMUSG00000023868:E022	-0.20	13.31	0.50	1	17	8930510	8930530
-	(+)	ENSMUSG00000023868:E023	-0.25	11.29	0.51	1	17	8930531	8930559
-	-	ENSMUSG00000023868:E024	-0.11	20.79	0.67	1	17	8941141	8941281
-	-	ENSMUSG00000023868:E025	-0.03	21.26	0.81	1	17	8942872	8943027
-	-	ENSMUSG00000023868:E026	-0.12	10.74	0.63	1	17	8944370	8944396
-	-	ENSMUSG00000023868:E027	-0.16	9.47	0.55	1	17	8944397	8944420
-	(+)	ENSMUSG00000023868:E028	-0.09	12.93	0.70	1	17	8944936	8944994
(+)	-	ENSMUSG00000023868:E029	10.37	0.13	0.51	1	17	8945569	8945716
-	-	ENSMUSG00000023868:E030	-0.24	10.80	0.45	1	17	8946796	8946847
-	-	ENSMUSG00000023868:E031	-0.17	26.03	0.44	1	17	8949350	8949492
-	-	ENSMUSG00000023868:E032	0.02	28.35	0.99	1	17	8951497	8951589
-	-	ENSMUSG00000023868:E033	-0.11	32.60	0.47	1	17	8953737	8953923
-	-	ENSMUSG00000023868:E034	-0.22	22.68	0.25	1	17	8961860	8962002
-	-	ENSMUSG00000023868:E035	-0.24	12.43	0.41	1	17	8962888	8962971
-	-	ENSMUSG00000023868:E036	-0.24	13.30	0.39	1	17	8964565	8964715
-	-	ENSMUSG00000023868:E037	-0.09	12.46	0.72	1	17	8967419	8967574
-	-	ENSMUSG00000023868:E038	0.13	18.02	0.69	1	17	8969528	8969700
-	-	ENSMUSG00000023868:E039	0.02	14.07	0.97	1	17	8974723	8974834
-	-	ENSMUSG00000023868:E040	0.16	17.99	0.67	1	17	8977136	8977216
-	-	ENSMUSG00000023868:E041	0.55	21.81	0.05	1	17	8978905	8978993
-	-	ENSMUSG00000023868:E042	0.19	29.70	0.39	1	17	8981533	8981671
-	-	ENSMUSG00000023868:E043	0.16	77.90	0.30	1	17	8981672	8982367
-	-	ENSMUSG00000023868:E044	0.42	5.55	0.49	1	17	8982368	8982391
-	-	ENSMUSG00000023868:E045	-0.05	5.58	0.87	1	17	8982392	8982400
-	-	ENSMUSG00000023868:E046	0.09	13.91	0.78	1	17	8982401	8982496
-	+	ENSMUSG00000023868:E047	0.06	158.83	0.54	1	17	8982497	8984288
-	+	ENSMUSG00000023868:E048	-0.05	166.22	0.34	1	17	8984289	8986645
-	+	ENSMUSG00000023868:E049	NA	0.00	NA	NA	17	8986646	8986648

Genome-wide differential exon usage in response to action potential (AP) firing in co-cultures of human iPSC-derived neurons and mouse primary hippocampal neurons (1) was determined with the DEXSeq package (103). Relative usage levels of the individual *Pde10a* (ENSMUSG00000023868) exons after 1 h of AP firing (indicated by $\log_2$ fold change relative to control condition, $\log_2$FC) were calculated. TSS, transcriptional start site; + indicates that the exon is exclusively used as a 5’ exon; (+) specifies a possible transcript 5’ end. 3’, + indicates that the exon is a 3’ exon; (+) specifies possible transcription termination. p_{Bonf} shows Bonferroni-corrected *p* values for the *Pde10a* exons. Genomic locations refer to mm10. n = 4 for both control condition and 1 h AP firing.

Table S4. Mouse *Pde10a* exon usage after 4 h action potential firing.

TSS	3’	Gene:exon	log_2FC	BaseMean	*p* value	p_{Bonf}	Chr	Start	End
+	-	ENSMUSG00000023868:E001	2.99	2.72	1.78E-04	7.81E-03	17	8525372	8525449
+	-	ENSMUSG00000023868:E002	3.37	15.72	1.46E-06	6.41E-05	17	8525450	8525815
+	-	ENSMUSG00000023868:E003	0.77	0.84	0.68	1	17	8526801	8527025
+	-	ENSMUSG00000023868:E004	0.65	2.14	0.34	1	17	8527026	8527115
(+)	-	ENSMUSG00000023868:E005	1.92	6.46	1.74E-05	7.65E-04	17	8756962	8757017
-	-	ENSMUSG00000023868:E006	1.56	7.86	7.37E-05	3.24E-03	17	8757018	8757093
-	(+)	ENSMUSG00000023868:E007	0.46	1.21	0.64	1	17	8758145	8758267
(+)	-	ENSMUSG00000023868:E008	NA	0.00	NA	NA	17	8798579	8798731
(+)	-	ENSMUSG00000023868:E009	-0.63	3.69	0.35	1	17	8801693	8801778
-	-	ENSMUSG00000023868:E010	-0.84	12.26	1.41E-02	0.62	17	8801779	8802145
(+)	-	ENSMUSG00000023868:E011	11.85	0.28	0.18	1	17	8803028	8803122
(+)	-	ENSMUSG00000023868:E012	-10.61	0.16	0.60	1	17	8849985	8850244
(+)	-	ENSMUSG00000023868:E013	NA	0.00	NA	NA	17	8880426	8880479
(+)	-	ENSMUSG00000023868:E014	-11.60	0.13	0.38	1	17	8893291	8893449
(+)	-	ENSMUSG00000023868:E015	0.22	20.70	0.61	1	17	8898861	8898989
(+)	-	ENSMUSG00000023868:E016	10.51	0.11	0.49	1	17	8898990	8899189
-	-	ENSMUSG00000023868:E017	NA	0.00	NA	NA	17	8899190	8899193
-	-	ENSMUSG00000023868:E018	-0.11	15.76	0.90	1	17	8920504	8920532
-	(+)	ENSMUSG00000023868:E019	1.87	0.50	0.44	1	17	8920533	8923426
(+)	-	ENSMUSG00000023868:E020	NA	0.00	NA	NA	17	8924143	8924241
-	-	ENSMUSG00000023868:E021	-0.06	24.58	0.84	1	17	8929092	8929212
-	-	ENSMUSG00000023868:E022	0.02	17.77	0.80	1	17	8930510	8930530
-	(+)	ENSMUSG00000023868:E023	-0.01	16.35	0.87	1	17	8930531	8930559
-	-	ENSMUSG00000023868:E024	-0.10	26.48	0.95	1	17	8941141	8941281
-	-	ENSMUSG00000023868:E025	0.16	26.54	0.37	1	17	8942872	8943027
-	-	ENSMUSG00000023868:E026	0.16	14.36	0.49	1	17	8944370	8944396
-	-	ENSMUSG00000023868:E027	-0.09	11.11	0.92	1	17	8944397	8944420
-	(+)	ENSMUSG00000023868:E028	-0.02	14.63	0.85	1	17	8944936	8944994
(+)	-	ENSMUSG00000023868:E029	-10.62	0.16	0.60	1	17	8945569	8945716
-	-	ENSMUSG00000023868:E030	0.28	15.45	0.24	1	17	8946796	8946847
-	-	ENSMUSG00000023868:E031	0.14	32.55	0.41	1	17	8949350	8949492
-	-	ENSMUSG00000023868:E032	0.04	34.25	0.75	1	17	8951497	8951589
-	-	ENSMUSG00000023868:E033	-0.06	42.77	0.90	1	17	8953737	8953923
-	-	ENSMUSG00000023868:E034	-0.41	30.63	0.19	1	17	8961860	8962002
-	-	ENSMUSG00000023868:E035	-0.61	19.30	0.10	1	17	8962888	8962971
-	-	ENSMUSG00000023868:E036	-0.11	16.16	0.80	1	17	8964565	8964715
-	-	ENSMUSG00000023868:E037	-0.32	13.76	0.37	1	17	8967419	8967574
-	-	ENSMUSG00000023868:E038	-0.07	22.81	0.88	1	17	8969528	8969700
-	-	ENSMUSG00000023868:E039	-0.05	21.60	0.85	1	17	8974723	8974834
-	-	ENSMUSG00000023868:E040	-0.33	24.71	0.19	1	17	8977136	8977216
-	-	ENSMUSG00000023868:E041	-0.16	28.65	0.58	1	17	8978905	8978993
-	-	ENSMUSG00000023868:E042	-0.04	44.45	0.92	1	17	8981533	8981671
-	-	ENSMUSG00000023868:E043	-0.04	85.44	0.97	1	17	8981672	8982367
-	-	ENSMUSG00000023868:E044	0.10	13.47	0.79	1	17	8982368	8982391
-	-	ENSMUSG00000023868:E045	-0.24	12.39	0.65	1	17	8982392	8982400
-	-	ENSMUSG00000023868:E046	0.28	22.00	0.51	1	17	8982401	8982496
-	+	ENSMUSG00000023868:E047	-0.01	204.14	0.90	1	17	8982497	8984288
-	+	ENSMUSG00000023868:E048	-0.18	270.25	0.39	1	17	8984289	8986645
-	+	ENSMUSG00000023868:E049	NA	0.00	NA	NA	17	8986646	8986648

Same as Table S3, except that relative usage levels of the individual *Pde10a* (ENSMUSG00000023868) exons after 4 h of AP firing were calculated. TSS, transcriptional start site; + indicates that the exon is exclusively used as a 5’ exon; (+) specifies a possible transcript 5’ end. 3’, + indicates that the exon is a 3’ exon; (+) specifies possible transcription termination. p_{Bonf} shows Bonferroni-corrected *p* values for the *Pde10a* exons. n = 4 for control condition, n = 3 for 4 h AP firing.

Table S5. Human and mouse *PDE10A*/*Pde10a* expression in response to action potential firing.

			$\log_2$FC	*p* value	p_{BH}
Human	1 h AP	*PDE10A*	-0.0200196	0.79926536	0.99997547
	4 h AP	*PDE10A*	0.18703277	0.08496301	0.54393695
Mouse	1 h AP	*Pde10a*	-0.0184323	0.79088655	0.99988168
	**4 h AP**	***Pde10a***	**0.43227841**	**0.00030663**	**0.00281088**

Genome-wide differential gene expression in response to action potential (AP) firing in co-cultures of human iPSC-derived neurons and mouse primary hippocampal neurons (1) was determined with the DESeq2 package (106). Shown are expression $\log_2$ fold change values relative to control condition ($\log_2$FC). p_{BH} shows Benjamini-Hochberg-corrected *p* values. n = 4 for both control condition and 1 h AP firing, n = 3 for 4 h AP firing. Mouse *Pde10a* upregulation at the four-hour time point is highlighted with bold text. Note that here the human genome assembly hg19 was used and the *Lnc473* gene was analyzed separately from *PDE10A* (1).

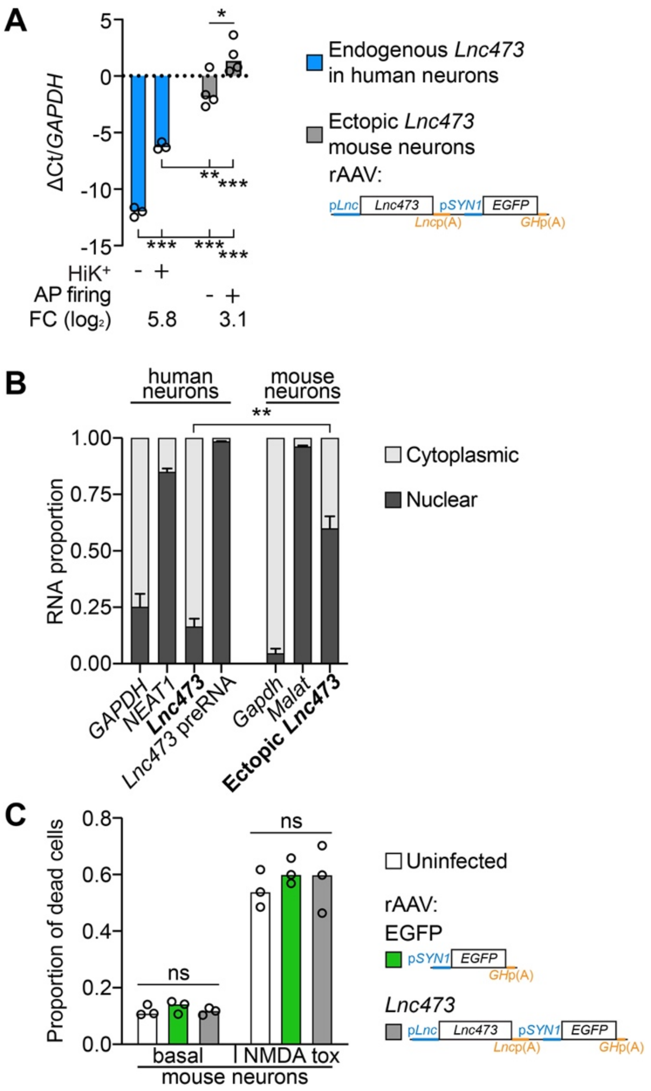

Figure S2. Characterization of ectopic *Lnc473* expression in mouse primary neurons.
Ectopic *Lnc473* was delivered into mouse primary hippocampal neurons (mouse neurons) by infection with an rAAV encoding the full-length human *Lnc473*.
A, RT-qPCR analysis of the expression levels of ectopic *Lnc473* in comparison to the endogenous *Lnc473* RNA levels in human iPSC-derived neurons (human neurons). *GAPDH* was used as a reference. Differences in qPCR Ct values of *Lnc473* and *GAPDH* (ΔCt/GAPDH) are shown. A high potassium concentration-containing solution (HiK$^+$, 50 mM, 4 h) or Bic (50 μM, 4 h) was used to induce *Lnc473* expression by depolarization or AP firing, respectively. FC, fold change.
B, Subcellular distribution of endogenous human *Lnc473* RNA and ectopic *Lnc473* in neurons. Separation of nuclear and cytoplasmic fractions followed by RNA extraction and RT-qPCR for detecting relative levels of the indicated targets was performed. *GAPDH* (or *Gapdh*) serves as a cytoplasmic mRNA marker. *NEAT1* and *Malat* are nuclear RNAs. *Lnc473* preRNA is unspliced *Lnc473*.
C, Quantification of cell death in mouse primary neuron cultures left uninfected, or infected with an rAAV encoding EGFP, or *Lnc473* and EGFP. For NMDA toxicity (NMDA tox) analysis, cells were treated with 30 μM NMDA for one hour and dead cells were counted 24 hours later.
Bars in *A* and *C* represent median. In *B* means ± SD are shown. n = 3, human neurons, and n = 4, mouse neurons (*A*,*B*); n = 3 (*C*). ns = not significant, *$p \le 0.05$, **$p \le 0.01$, ***$p \le 0.001$. *A* and *C*, One-way ANOVAs ($F_{3, 10}$ = 88.27, *A*; $F_{2, 6}$ = 0.67, *C* basal; $F_{2, 6}$ = 0.43, *C* NMDA tox) with Tukey´s tests. *B*, t_5 = 6.03, two-tailed *t*-test.

Table S6. Significantly (p_{adj} ≤ 0.1) upregulated genes in *Lnc473*-expressing mouse primary hippocampal neurons (DESeq2 results).

	Gene	BaseMean	log_2FC	*p* value	$p_{adj(BH)}$
	Fibin	1075.7	0.21	3.56E-08	2.41E-05
	Phgdh	2219.4	0.20	5.35E-09	8.46E-06
*	***Ddit3***	5317.8	0.19	2.20E-10	6.94E-07
	Snhg5	761.6	0.19	8.61E-07	0.00029
*	***Atf3***	759.1	0.18	6.58E-07	0.00023
*	***Ppp1r15a***	2096.7	0.18	1.89E-07	8.55E-05
	P2rx3	1644.8	0.18	1.11E-06	0.00034
*	***Fam150b***	1742.1	0.17	5.38E-08	3.25E-05
	Cyb5r1	7882.7	0.17	1.32E-08	1.40E-05
	2410006H16Rik	2236.3	0.16	1.82E-06	0.00044
*	***Nfil3***	1268.3	0.16	3.29E-06	0.00074
	Pnrc2	2615.7	0.16	1.26E-07	7.05E-05
	Chchd1	916.2	0.15	2.68E-05	0.00303
	Snhg1	721.3	0.14	0.00012	0.0082
*	***Bdnf***	5197.8	0.14	5.48E-08	3.25E-05
	Kctd15	2407.8	0.14	1.95E-05	0.00253
	Tigd2	1182.0	0.14	2.62E-05	0.00301
	Lhfpl2	2166.9	0.14	4.45E-05	0.00405
	Sft2d1	772.7	0.14	0.00014	0.0091
	Zcchc12	11407.5	0.14	1.49E-08	1.42E-05
	LOC106740	2612.1	0.14	2.42E-06	0.00056
	Cort	1086.2	0.13	0.00016	0.00977
	Pycr1	838.1	0.13	0.00038	0.01732
*	***Rheb***	11019.2	0.13	2.62E-11	1.24E-07
	Krtcap2	2097.4	0.13	1.51E-05	0.00221
	Snhg12	1114.5	0.13	0.00044	0.01886
	Dhrs7	1799.3	0.13	8.25E-05	0.00632
	Zxdb	853.7	0.13	0.00049	0.02007
	Atp6v1g1	3801.8	0.13	1.87E-06	0.00044
*	***Dusp4***	2263.2	0.12	6.40E-05	0.00519
*	***Psph***	2228.3	0.12	6.65E-05	0.00535
	Leprotl1	7966.1	0.12	5.57E-07	0.00021
	Ankrd44	951.8	0.12	0.00082	0.02788
	4921524J17Rik	929.7	0.12	0.00062	0.02332
	Angptl6	1285.1	0.12	0.00037	0.01718
	Elovl7	541.6	0.12	0.00168	0.04063
*	***Vgf***	37967.4	0.12	0.00128	0.03517
*	***Wbp5***	3420.6	0.12	2.20E-05	0.0027
	Foxn2	760.5	0.12	0.00139	0.03722
	Wwtr1	615.4	0.12	0.00171	0.04118
	Slc7a11	926.3	0.12	0.00158	0.03982
	Nmd3	1429.9	0.12	0.00045	0.01891
	Nfkbil1	904.2	0.12	0.00141	0.03736
	Serpinf1	733.7	0.12	0.00191	0.04358
	Hnrnph3	2749.6	0.12	3.21E-05	0.00332
*	***Nr4a1***	1698.2	0.12	0.0012	0.03412
	Gja1	1854.3	0.12	0.0006	0.02297
	Tceal8	1905.0	0.11	0.0002	0.01158
	Mrpl24	1526.6	0.11	0.00097	0.03039
*	***Spry2***	3064.1	0.11	0.00014	0.00919
*	***Cth***	569.0	0.11	0.0031	0.05939
*	***Hspa5***	20479.8	0.11	9.54E-09	1.13E-05
	Sema3a	723.8	0.11	0.00281	0.05606
	Slc7a3	4977.3	0.11	6.09E-06	0.00119
	Nrip2	792.1	0.11	0.00311	0.05939
	Enox2	1662.0	0.11	0.00065	0.02385
	Metrn	913.2	0.11	0.00228	0.04897
*	***Cbfb***	1741.2	0.11	0.00099	0.03067
	Eif2s2	7205.2	0.11	4.95E-07	0.0002
*	***Nxn***	553.3	0.11	0.00522	0.08282
	Atp1a2	4953.4	0.11	0.00025	0.01365
	Bcat1	12572.2	0.11	9.67E-07	0.00031
	Apoe	31600.5	0.11	0.00014	0.0091
	Lonp1	8129.3	0.10	1.50E-05	0.00221
	Cnppd1	1788.3	0.10	0.00096	0.03033
	Rars	4085.5	0.10	6.35E-05	0.00519
	6330416G13Rik	1073.8	0.10	0.00241	0.05094
	Dnaja3	5927.8	0.10	1.56E-05	0.00223
	Rps27a	4900.9	0.10	4.36E-05	0.00405
	Them4	1830.2	0.10	0.00116	0.03358
	Lars	7302.1	0.10	1.67E-05	0.00233
	Ccdc59	1001.6	0.10	0.00378	0.06704
	Aqp4	2227.0	0.10	0.00385	0.06762
	Hat1	866.4	0.10	0.00483	0.07897
	Hsd17b12	7835.6	0.10	5.22E-05	0.00443
	Zc2hc1a	7221.9	0.10	3.71E-06	0.0008
	Mrto4	973.3	0.10	0.00702	0.09755
	Nutf2	990.4	0.10	0.00615	0.09117
*	***Clic4***	3591.5	0.10	0.00128	0.03517
*	***Slc1a3***	7760.5	0.10	0.00025	0.01371
	Arhgap12	5505.2	0.10	0.00016	0.01008
	Tmed5	2030.1	0.10	0.00118	0.03375
	Exosc2	896.4	0.10	0.00584	0.08924
	Pfdn4	1208.2	0.10	0.00352	0.06408
	Lims1	2790.2	0.10	0.00058	0.02266
*	***Odc1***	3222.7	0.10	0.00042	0.01817
*	***Cebpb***	1294.0	0.10	0.00488	0.07944
	Rps14	12381.5	0.10	0.00014	0.00919
	Fabp7	2598.0	0.10	0.00111	0.03278
	Vmp1	6201.7	0.10	2.17E-05	0.0027
*	***Atf4***	19864.0	0.10	1.08E-05	0.00179
	Rnf138	1192.1	0.10	0.00467	0.07795
	Hax1	1566.0	0.10	0.0044	0.0748
*	***Shmt2***	4211.5	0.10	0.0007	0.02497
	Lix1	2849.0	0.10	0.00062	0.02332
	Gkap1	985.8	0.10	0.00617	0.09117
	Esf1	2157.5	0.09	0.00121	0.03415
*	***Gla***	1632.0	0.09	0.0027	0.05462
	Rhoq	2999.1	0.09	0.00052	0.02081
	Aldoc	1995.4	0.09	0.00685	0.09671
*	***Cnbp***	14342.1	0.09	5.00E-05	0.00432
	Mknk1	1666.4	0.09	0.00286	0.05684
	Camk1g	2418.0	0.09	0.00095	0.03032
	Eif3e	5074.9	0.09	6.04E-05	0.00507
	Tspan6	1841.5	0.09	0.00244	0.05117
	Tcea1	4212.2	0.09	0.00048	0.01982
	Gsto1	2441.5	0.09	0.00119	0.03397
	Pdcd7	1830.1	0.09	0.0032	0.06025
	Nop58	2521.1	0.09	0.00144	0.03787
	Mrps18a	2363.3	0.09	0.00173	0.04143
	Bcas2	3230.9	0.09	0.00099	0.03068
	Hypk	2117.3	0.09	0.00341	0.06263
	Adh5	4078.4	0.09	0.00077	0.02695
	Zfp622	2893.9	0.09	0.00109	0.03245
	Bcan	1649.5	0.09	0.00716	0.09844
	Tra2b	2443.7	0.09	0.00413	0.07157
*	***Eprs***	13225.5	0.09	2.13E-05	0.0027
	Tbca	3717.3	0.09	0.00054	0.02147
	Cetn3	1918.2	0.09	0.00315	0.05989
	Rbbp4	6759.0	0.09	0.00027	0.0141
*	***Hbs1l***	4696.6	0.09	0.00045	0.01891
	Ube2w	6643.7	0.09	7.55E-05	0.0059
*	***Ncbp2***	3020.9	0.09	0.0009	0.02954
	Chac1	3854.5	0.09	0.00436	0.0745
	Smim15	2799.0	0.09	0.00111	0.03269
	Iqcb1	1368.6	0.09	0.00725	0.09881
	Rps24	7922.5	0.09	0.00128	0.03517
	Fam219b	1518.8	0.09	0.00636	0.09363
	Mphosph10	1587.7	0.09	0.00491	0.0797
*	***Junb***	2329.1	0.09	0.00302	0.0586
	Pop4	1771.5	0.09	0.00521	0.08282
	Rpl7	8456.3	0.09	0.00013	0.00868
	Eif3g	3924.7	0.09	0.00105	0.03152
	Rpl5	9128.9	0.09	0.00012	0.00836
	Fam45a	1842.8	0.09	0.00727	0.09881
	Igip	3363.3	0.09	0.00153	0.03912
	Vbp1	4342.9	0.09	0.00047	0.01958
	Sypl	2105.7	0.09	0.00372	0.0664
	Creb3	3135.2	0.09	0.00121	0.03415
	Cabp1	2769.4	0.09	0.00328	0.06113
	Mpdu1	2039.1	0.09	0.00512	0.08202
	Trim66	4893.3	0.09	0.0051	0.08202
	Asnsd1	2017.8	0.09	0.00512	0.08202
*	***Crhbp***	3885.4	0.09	0.00118	0.03375
	Cst3	9220.7	0.09	0.00105	0.03152
	Nrip3	14986.9	0.09	0.0002	0.01183
	Arpc5l	5084.0	0.09	0.00067	0.0244
	Arxes2	1890.9	0.09	0.00611	0.09117

	Gene	BaseMean	log_2FC	*p* value	$p_{adj(BH)}$
	Cebpg	3015.4	0.09	0.00133	0.03603
	Rpl35a	2832.9	0.09	0.00355	0.06435
	Ppp1cb	19735.0	0.09	1.66E-06	0.00041
	Hectd2	2835.0	0.08	0.00217	0.04731
	Hspa9	29298.8	0.08	1.26E-05	0.00199
	Asns	18719.2	0.08	1.00E-05	0.0017
	Eif5	14202.0	0.08	6.16E-06	0.00119
	Gars	20320.3	0.08	1.22E-05	0.00197
	Farsb	5369.8	0.08	0.00091	0.02963
	Pdcd6	2395.8	0.08	0.0034	0.06263
	Arl14ep	4778.7	0.08	0.00051	0.02053
	Cript	2121.8	0.08	0.00499	0.08076
*	***Egr1***	5185.9	0.08	0.00645	0.09444
*	***Slc3a2***	18947.1	0.08	0.0004	0.01762
	D19Bwg1357e	2960.2	0.08	0.00238	0.05051
	Rpl32	7032.6	0.08	0.00139	0.03722
	Aarsd1	2395.5	0.08	0.00585	0.08924
	Anp32e	7946.9	0.08	0.00066	0.0241
*	***Stxbp6***	2977.6	0.08	0.00543	0.0846
	Rps23	4750.3	0.08	0.00081	0.02758
	Eif1	10941.7	0.08	8.40E-05	0.00638
	Mthfd2	3570.2	0.08	0.00413	0.07157
	Gtpbp4	2567.9	0.08	0.00312	0.05953
	Mdp1	2755.4	0.08	0.00331	0.06153
*	***3110043O21Rik***	2645.9	0.08	0.00583	0.08924
	Rpl6	6801.1	0.08	0.00031	0.01569
	Gstm1	6331.1	0.08	0.00223	0.04828
	Cept1	2558.2	0.08	0.00598	0.09046
	Ubxn1	3291.3	0.08	0.00586	0.08925
	C1d	2816.3	0.08	0.00678	0.09659
	Rps18	5643.2	0.08	0.00179	0.0423
	Plcl2	4313.5	0.08	0.00271	0.05473
	Rps8	7339.1	0.08	0.00094	0.03022
	Eps15	31237.7	0.08	0.00032	0.01608
	Sla	3474.2	0.08	0.0023	0.04945
	Rhoa	6747.0	0.08	0.00028	0.01431
	Ssr3	7181.9	0.08	0.00026	0.01399
	Rps3	15423.3	0.08	0.00251	0.05163
	Nars	28742.9	0.08	0.00016	0.01007
	Ccdc47	8965.6	0.08	0.00017	0.01045
	Rpl13a	12904.4	0.08	0.00283	0.05643
	Hsp90b1	16821.1	0.08	0.0001	0.00724
	Yars	14011.3	0.08	0.00126	0.03508
	Gtpbp6	2605.5	0.08	0.00608	0.09093
	Arl2	2625.0	0.08	0.00707	0.09784
	Bag5	2516.7	0.08	0.00647	0.0945
	C1qbp	3426.7	0.08	0.00408	0.07113
	Cpe	103432.6	0.08	0.00035	0.01695
	Rps12	7599.5	0.08	0.00206	0.04568
	Tlk2	3796.9	0.08	0.00352	0.06407
	Sdhb	7610.5	0.08	0.00066	0.02398
*	***Cdkn1b***	4248.1	0.08	0.00245	0.05117
	Usmg5	5182.0	0.08	0.00448	0.07585
*	***Scg2***	11504.4	0.08	0.00043	0.01886
	Stard3nl	3160.9	0.08	0.00545	0.08479
	Ptges3	4964.9	0.08	0.00156	0.03982
*	***Syt4***	18831.4	0.08	8.74E-05	0.00654
*	***Psmc6***	5857.6	0.08	0.00094	0.03022
	Rundc3b	3128.8	0.08	0.00477	0.07853
	Thoc7	2426.9	0.08	0.00717	0.09844
	Psmb4	7736.3	0.08	0.00175	0.04169
	Ormdl3	3963.7	0.08	0.003	0.05837
	Sept6	5285.0	0.07	0.00162	0.03982
	Vamp4	3245.1	0.07	0.00482	0.07897
	Eif2s1	3189.8	0.07	0.00552	0.08567
	Eif3d	4685.9	0.07	0.00188	0.04297
*	***Rabggtb***	3126.4	0.07	0.00487	0.07944
	Mettl9	3657.5	0.07	0.00483	0.07897
	Rps15a	6993.7	0.07	0.00104	0.03152
	Fam174a	3284.5	0.07	0.00605	0.09093
	Rpl22	7320.1	0.07	0.00277	0.05557
	Rtca	2967.0	0.07	0.00641	0.09411
	Abhd17b	3406.3	0.07	0.00542	0.0846
*	***Wars***	9057.5	0.07	0.00689	0.09681
	Serpine2	5638.1	0.07	0.00158	0.03982
	Tars	6658.4	0.07	0.00138	0.03714
*	***Tpt1***	13622.6	0.07	0.00075	0.02644
	Kcnk2	3804.9	0.07	0.00439	0.07475
	Psmd14	4698.1	0.07	0.00594	0.0902
	Ubr7	3125.5	0.07	0.00676	0.09659
	Srsf3	4928.6	0.07	0.00372	0.0664
	Wbp4	4852.7	0.07	0.00308	0.05936
	Fbxo9	5162.2	0.07	0.00348	0.06352
*	***Hspe1***	3443.9	0.07	0.00692	0.09685
	Hspd1	13645.3	0.07	0.00062	0.02332
	Eif3a	16525.3	0.07	0.00037	0.01732
	Atp6ap2	24837.2	0.07	0.0002	0.01169
	Map7d2	14887.3	0.07	0.00035	0.01695
	Dnaja2	12632.0	0.07	0.00022	0.01296
	Ddit4	7519.8	0.07	0.00417	0.07191
	Rps3a1	8496.6	0.07	0.0016	0.03982
*	***Cxadr***	11545.2	0.07	0.0022	0.04779
	Atp5h	6618.0	0.07	0.00558	0.08626
	Psmb7	7774.6	0.07	0.00251	0.05163
	Eif3c	19943.7	0.07	0.00058	0.02266
	Atp1b3	3513.3	0.07	0.00736	0.09934
*	***Gng4***	11872.0	0.07	0.00337	0.0623
	Ptp4a2	9816.2	0.07	0.00377	0.06704
*	***Eif4e***	5502.9	0.07	0.00471	0.0783
	Bzw2	5397.2	0.07	0.00667	0.096
	Sar1a	8158.8	0.07	0.00214	0.0467
	Psmd13	5149.4	0.07	0.00727	0.09881
	Arpp19	7242.2	0.07	0.0026	0.05299
	Chmp2b	5460.6	0.07	0.00483	0.07897
	Atg12	7321.0	0.07	0.00242	0.05107
	Tmem47	8322.5	0.07	0.00146	0.03809
*	***Rpl10***	5425.5	0.07	0.00689	0.09681
	Chtop	9192.7	0.07	0.0012	0.03413
	Zmat2	8052.9	0.06	0.00608	0.09093
	Tmem59	7475.9	0.06	0.00368	0.06593
	Tagln3	6270.1	0.06	0.00691	0.09685
*	***Hars***	7422.9	0.06	0.00299	0.05822
	Mff	10680.0	0.06	0.00144	0.03787
	Rpsa	17701.5	0.06	0.00623	0.09178
	Fez1	15171.9	0.06	0.00647	0.0945
	Serbp1	13950.2	0.06	0.00125	0.03493
	Cdk5r1	25096.3	0.06	0.00477	0.07853
	Sec62	13462.4	0.06	0.00109	0.03245
*	***Cct4***	8065.5	0.06	0.00421	0.07245
	Ghitm	30679.7	0.06	0.00113	0.03313
	Gpm6b	56213.4	0.06	0.00039	0.01762
	Npm1	9023.2	0.06	0.00411	0.07147
	Pkia	18008.7	0.06	0.00256	0.0526
	Rpl9	10532.8	0.06	0.00567	0.08715
	Cct8	14107.4	0.06	0.00146	0.03809
	Rpl15	7501.6	0.06	0.00419	0.07221
	Rap1b	6288.5	0.06	0.00704	0.09775
	Rpl4	24697.5	0.06	0.00187	0.04297
	Rpl3	14615.4	0.06	0.00324	0.0605
*	***Etf1***	8456.0	0.06	0.00459	0.07704
	Tmem66	20162.5	0.06	0.00295	0.05792
	Ube2n	8110.0	0.06	0.00617	0.09117
	Atp6v1h	12829.4	0.06	0.00266	0.05391
	Eef1a1	99025.0	0.06	0.00293	0.05792
	Tcp1	12226.0	0.06	0.0053	0.08333
	Tmed10	8871.3	0.06	0.00674	0.09659
	Ncl	17516.8	0.06	0.00346	0.06324
	Cct7	13166.1	0.05	0.00512	0.08202
	Rbbp7	10949.5	0.05	0.00618	0.09117
	Atp6v1c1	15459.0	0.05	0.00507	0.08164
	Pgrmc1	32730.6	0.05	0.00236	0.05027
	Rab14	16303.1	0.05	0.00514	0.08208

Marked with asterisks and in bold are 49 CREB target genes. Total of 1247 CREB targets with experimental evidence (and present in the RNA-seq analysis performed here) were acquired and combined from the following publications:

- Impey *et al*., Cell 2004 (116)
- Zhang *et al*., PNAS 2005 (117)
- Xu *et al*., EMBO J 2007 (118)
- Benito *et al*., J Neurosci. 2011 (63)
- Pardo *et al*., Scientific Rep. 2017 (119)

Table S7. Significantly (p_{adj} ≤ 0.1) downregulated genes in *Lnc473*-expressing mouse primary hippocampal neurons (DESeq2 results).

Gene	BaseMean	log_2FC	*p* value	$p_{adj(BH)}$
Nos1	994.6	-0.19	1.72E-07	8.19E-05
Flna	7830.7	-0.17	4.34E-10	1.03E-06
Col6a1	929.1	-0.16	1.80E-05	0.0024
Kif26b	574.0	-0.16	1.78E-05	0.0024
Col5a1	726.6	-0.16	3.67E-05	0.00359
Lnpep	1623.3	-0.16	1.11E-05	0.00181
Kcnt1	1457.1	-0.16	6.63E-06	0.00126
Hist1h1c	1200.8	-0.16	7.83E-06	0.0014
Pidd1	567.6	-0.15	6.33E-05	0.00519
Htr1a	967.2	-0.15	2.37E-05	0.00281
Zbed6	811.5	-0.15	7.64E-05	0.0059
Cemip	5802.6	-0.15	1.93E-08	1.52E-05
Bzrap1	7823.8	-0.15	7.73E-09	1.05E-05
Ldlr	1950.7	-0.15	3.24E-05	0.00332
Map4k2	1429.7	-0.15	2.04E-05	0.00261
Syngap1	16999.7	-0.14	6.55E-12	6.22E-08
Prox1	1276.7	-0.14	4.42E-05	0.00405
Ryr3	3004.0	-0.14	1.21E-06	0.00034
Flnb	1619.2	-0.14	4.88E-05	0.00429
Ssh3	743.2	-0.14	0.0001	0.00919
Cacna1h	4854.9	-0.14	1.49E-07	7.45E-05
Mdga1	2878.0	-0.14	9.80E-07	0.00031
Pxdn	2822.2	-0.14	5.25E-06	0.00108
Tenm1	2177.1	-0.14	2.81E-05	0.00314
Cit	3078.6	-0.14	1.21E-06	0.00034
Nefh	3733.3	-0.14	1.61E-06	0.00041
Col12a1	708.3	-0.13	0.0003	0.01517
Igsf9b	1798.2	-0.13	0.0002	0.01002
Abca7	1656.7	-0.13	4.17E-05	0.00399
D130043K22Rik	2435.7	-0.13	6.81E-06	0.00127
Tmem245	1430.5	-0.13	0.0001	0.00739
Med12	933.9	-0.13	0.0003	0.01485
Kcnc1	9853.4	-0.13	3.79E-09	7.19E-06
Lynx1	7822.5	-0.13	4.67E-07	0.00019
Celsr3	5375.4	-0.13	3.38E-06	0.00075
Serinc5	1480.8	-0.13	0.0001	0.00739
Lrrc45	1383.4	-0.13	0.0004	0.01697
Kcnc3	5550.3	-0.13	1.75E-08	1.51E-05
Cabp7	689.0	-0.13	0.0007	0.02553
Firre	1867.5	-0.13	0.0001	0.00895
Ksr2	1670.1	-0.13	0.0005	0.01942
Vps13c	2563.0	-0.13	0.0003	0.01431
Trpm3	1047.7	-0.13	0.0005	0.02129
Col4a2	2461.1	-0.12	7.43E-05	0.0059
Lss	1564.5	-0.12	0.0002	0.01363
Grin2a	1082.1	-0.12	0.0009	0.02855
Tnr	2634.2	-0.12	0.0004	0.01732
Nhsl2	1997.7	-0.12	0.0002	0.01093
Vip	1020.1	-0.12	0.001	0.03152
Plec	5159.2	-0.12	2.34E-05	0.00281
Tbc1d8	961.7	-0.12	0.0008	0.02788
Traf3	11050.4	-0.12	2.19E-08	1.60E-05
Abcc8	1068.1	-0.12	0.0009	0.03003
Col4a1	1968.8	-0.12	0.0004	0.01757
Fryl	2420.4	-0.12	0.0004	0.01762
Kndc1	3872.3	-0.12	1.51E-05	0.00221
Smpd4	1558.3	-0.12	0.0004	0.01695
Fndc1	651.8	-0.12	0.0025	0.05131
Ryr2	5240.2	-0.12	7.62E-05	0.0059
Slc9a7	1867.1	-0.12	0.0005	0.0216
Plxdc2	2681.1	-0.11	0.0001	0.0091
Trerf1	632.7	-0.11	0.0023	0.04869
Usp54	771.3	-0.11	0.002	0.045
Myo5b	5995.5	-0.11	1.26E-06	0.00034
Dgki	1400.4	-0.11	0.001	0.03068
Cyth1	4244.5	-0.11	2.56E-05	0.003
Adcy6	2921.5	-0.11	4.52E-05	0.00405
Fat4	2170.7	-0.11	0.0003	0.01695
Kmt2d	5635.2	-0.11	0.0001	0.00762
Adam11	7664.2	-0.11	5.01E-05	0.00432
Kcnab3	630.0	-0.11	0.0029	0.05755
Rps6kb2	647.9	-0.11	0.0031	0.05939
Myo9b	1746.7	-0.11	0.0017	0.04062
Cdk14	8043.0	-0.11	6.21E-07	0.00023
Ano3	591.1	-0.11	0.0034	0.06263
Nptx1	13245.3	-0.11	9.67E-06	0.00167
Scn1a	5545.2	-0.11	3.81E-05	0.00369
Klf12	1567.3	-0.11	0.001	0.03113
Dscaml1	3793.9	-0.11	2.98E-05	0.00325
Uhmk1	4517.4	-0.11	0.0004	0.01732
1700003E16Rik	1120.5	-0.11	0.0013	0.03552
Ncoa1	9026.9	-0.11	4.04E-07	0.00017
Trpc5	751.4	-0.11	0.0045	0.07562
Zkscan16	1658.2	-0.11	0.0009	0.02906
Hsd17b7	2558.5	-0.11	0.0003	0.0141
Gpr26	2659.5	-0.11	0.0006	0.02308
Plekha6	3690.7	-0.11	2.63E-05	0.00301
Fasn	35983.5	-0.11	1.49E-05	0.00221
Mirg	665.4	-0.11	0.0045	0.07612
Lmbrd2	3244.7	-0.11	0.0004	0.01886
Dgkq	2381.1	-0.11	0.0003	0.01371
Adamts10	655.9	-0.11	0.0046	0.07704
Gse1	2967.6	-0.11	0.0001	0.00821
Hrh3	1004.1	-0.11	0.0034	0.06263
Tmem181b-ps	6795.7	-0.11	0.0002	0.01298
Abca2	13873.9	-0.11	1.17E-06	0.00034
Xkr4	3393.3	-0.11	0.0057	0.08714
Ltbp4	1832.8	-0.11	0.0016	0.03982
Dennd6b	2329.5	-0.11	0.0004	0.01721
Parm1	1329.0	-0.11	0.0017	0.04122
Akap13	1780.2	-0.11	0.003	0.05883
Cux2	2078.2	-0.11	0.0006	0.02288
Zfp692	1011.2	-0.11	0.0033	0.06186
Zkscan2	702.4	-0.11	0.0052	0.08229
Ccdc39	738.6	-0.10	0.0047	0.0783
Zfp536	1508.7	-0.10	0.0022	0.04814
Lime1	1624.6	-0.10	0.0024	0.05051
Dgkh	708.6	-0.10	0.0067	0.09584
Syt17	2410.7	-0.10	0.0005	0.02154
Ccdc88c	3028.1	-0.10	0.0003	0.01431
Sema4g	3932.9	-0.10	4.65E-05	0.00413
Ptchd2	1399.3	-0.10	0.002	0.0451
Snapc4	1904.0	-0.10	0.0009	0.02964
Sptbn4	11084.4	-0.10	4.50E-05	0.00405
Csmd2	3410.9	-0.10	0.0004	0.01695
Adam19	1807.2	-0.10	0.0015	0.0383
Tshz3	1371.3	-0.10	0.003	0.05822
Stxbp2	1140.9	-0.10	0.006	0.09046
Sidt1	1256.2	-0.10	0.0029	0.05792
Plxna3	3821.9	-0.10	0.0002	0.01296
Atp13a2	15080.4	-0.10	1.37E-07	7.24E-05
Ldlrad4	755.4	-0.10	0.0066	0.09521
Fat1	2517.4	-0.10	0.0023	0.04954
Myo1e	722.8	-0.10	0.0068	0.09659
Rapgefl1	8194.1	-0.10	1.50E-06	0.0004
Vwa5b2	718.2	-0.10	0.0072	0.09865
Zfp335	1341.1	-0.10	0.0025	0.05163
Soga1	7003.0	-0.10	3.21E-05	0.00332
Acly	20256.1	-0.10	4.00E-06	0.00084
Epha10	2166.0	-0.10	0.0035	0.06434
Slc6a6	4740.7	-0.10	6.22E-05	0.00518
Vps13a	2794.9	-0.10	0.002	0.04536
Pag1	2309.5	-0.10	0.0007	0.0247
Gabbr2	12694.8	-0.10	1.88E-05	0.00248
Hist1h2bc	1453.3	-0.10	0.0033	0.06208
Ophn1	1774.5	-0.10	0.0016	0.03982
Mbd6	1286.1	-0.10	0.0065	0.09486
Celf3	7611.9	-0.10	2.22E-05	0.0027
Megf11	1085.4	-0.10	0.0055	0.08578
Bahcc1	1822.9	-0.10	0.0016	0.03982
Ncoa3	2331.4	-0.10	0.0013	0.03552
Rasgrf2	2422.7	-0.10	0.0006	0.02385
Ints1	6938.1	-0.10	4.25E-05	0.00399
Fcho1	3900.5	-0.10	0.0008	0.02824
Etv1	1692.7	-0.10	0.0021	0.04661
Kcnq3	2996.3	-0.10	0.0032	0.06006
Sipa1l3	5207.8	-0.10	0.0003	0.01655
Ncam2	4214.7	-0.10	0.0006	0.02332

Gene	BaseMean	log_2FC	*p* value	$p_{adj(BH)}$
Nsmaf	1047.1	-0.10	0.0056	0.08674
Epha6	1895.2	-0.10	0.0042	0.07191
Abcc5	5432.2	-0.10	0.0003	0.01371
Csf2ra	923.6	-0.10	0.0071	0.09841
Mef2d	5091.6	-0.10	3.15E-05	0.00332
Dhcr7	5446.8	-0.10	0.0005	0.02007
Megf9	4239.9	-0.10	9.75E-05	0.00712
Peak1	1937.4	-0.10	0.0024	0.05117
Kcnn3	1220.4	-0.10	0.0072	0.09865
Cd24a	4529.4	-0.10	0.0006	0.02378
Crebbp	2258.3	-0.10	0.0032	0.06037
Szt2	1809.9	-0.10	0.0066	0.09521
Nacc2	5866.9	-0.10	0.0003	0.01405
Pitpnm2	10496.2	-0.10	5.62E-06	0.00114
BC068157	2287.3	-0.09	0.001	0.03068
Peg3	11281.0	-0.09	0.0001	0.00897
Kit	3834.4	-0.09	0.0004	0.01802
Tmtc1	2631.4	-0.09	0.001	0.03073
Fn1	1736.9	-0.09	0.0047	0.07795
Nin	2341.8	-0.09	0.0018	0.04198
Pkd1	5677.1	-0.09	0.0008	0.02725
Adcy9	3815.1	-0.09	0.0004	0.01757
Clmn	3073.9	-0.09	0.0009	0.02954
Pisd-ps1	4965.3	-0.09	0.0011	0.03196
Dyrk2	2421.2	-0.09	0.0015	0.03856
Aldh4a1	1803.4	-0.09	0.0025	0.05163
Rps6ka5	1608.1	-0.09	0.003	0.058
Miat	8139.7	-0.09	0.0073	0.09897
Kmt2a	6467.3	-0.09	0.0002	0.01298
Dnajb14	1182.7	-0.09	0.0069	0.09681
Phldb1	1522.9	-0.09	0.0037	0.0664
Lpin1	1666.0	-0.09	0.0032	0.06037
Cacna1c	6536.1	-0.09	8.75E-05	0.00654
Prrt1	2776.6	-0.09	0.0009	0.02942
Ncor2	7621.8	-0.09	0.0003	0.01399
Kcnma1	5830.2	-0.09	0.0016	0.03982
Mical3	3703.2	-0.09	0.0015	0.03836
Eml1	2528.6	-0.09	0.0018	0.04273
Hip1	5004.5	-0.09	0.0002	0.01339
Sik2	1263.8	-0.09	0.007	0.09716
Elfn2	6023.3	-0.09	0.0004	0.01703
Atg2a	3326.0	-0.09	0.0015	0.03808
Setd1b	2776.7	-0.09	0.0018	0.04191
Ptprs	36215.7	-0.09	9.10E-06	0.0016
Ptpn23	4688.3	-0.09	0.0008	0.02725
Gpr123	5211.3	-0.09	0.0012	0.03365
Spns2	3412.2	-0.09	0.001	0.03033
Zbtb16	1794.6	-0.09	0.0056	0.08674
Caln1	4648.6	-0.09	0.0007	0.02398
Rimbp2	5404.6	-0.09	9.98E-05	0.00723
Safb2	2125.9	-0.09	0.0036	0.06548
Iqsec1	13124.7	-0.09	3.29E-05	0.00332
Homer2	4258.2	-0.09	0.0016	0.03982
Ece1	1319.7	-0.09	0.0071	0.09784
Flrt2	2712.6	-0.09	0.0026	0.05373
Srrt	4180.9	-0.09	0.0009	0.029
Slc36a1	3115.9	-0.09	0.0008	0.02725
Plxna4	10039.1	-0.09	0.0014	0.03722
Fbf1	4077.4	-0.09	0.0007	0.02562
Kcnj11	1371.9	-0.09	0.0073	0.09934
Fam212b	2615.8	-0.09	0.0018	0.04234
Dip2a	4167.4	-0.09	0.0006	0.02247
Pcdh10	6881.2	-0.09	0.0004	0.01891
Hspa12a	24376.9	-0.09	1.45E-05	0.00221
Kifc2	13056.5	-0.09	0.0013	0.03502
Ep300	3931.8	-0.09	0.0029	0.05792
Rprm	2334.9	-0.09	0.0026	0.0526
Cpt1c	7938.2	-0.09	7.60E-05	0.0059
Nxf1	2729.5	-0.09	0.002	0.04528
Dhcr24	11348.8	-0.09	0.0007	0.02512
Ociad2	2695.2	-0.09	0.002	0.04536
Slc41a1	3983.1	-0.09	0.0008	0.02725
Iqsec2	9505.8	-0.09	4.24E-05	0.00399
Tnrc18	15919.0	-0.09	3.67E-05	0.00359
Spock1	13253.4	-0.09	7.47E-06	0.00136
Paqr9	2494.6	-0.09	0.0031	0.05939
Med13l	5252.9	-0.09	0.001	0.03113
Abhd2	2885.0	-0.09	0.0038	0.06731
Fras1	2070.8	-0.08	0.0068	0.09659
Unc13a	24195.7	-0.08	1.57E-05	0.00223
Dock4	8616.6	-0.08	0.0006	0.02378
Pcdh19	5738.2	-0.08	0.0003	0.01695
Slc4a3	8964.1	-0.08	5.18E-05	0.00443
Mast3	8826.1	-0.08	0.0001	0.00836
Dnm3	7431.1	-0.08	0.001	0.03033
Cluh	6202.1	-0.08	0.0007	0.02458
Zzef1	3736.6	-0.08	0.0018	0.04234
Cic	13847.9	-0.08	3.25E-05	0.00332
Galntl6	2294.5	-0.08	0.0046	0.07666
Btbd11	3717.8	-0.08	0.0017	0.04045
Slc7a4	2848.1	-0.08	0.0039	0.06773
Nacad	3084.0	-0.08	0.0021	0.04654
D430041D05Rik	7689.4	-0.08	0.0006	0.02308
Gas6	5544.1	-0.08	0.0014	0.03787
Kmt2b	3583.5	-0.08	0.0026	0.05299
Adam23	9466.9	-0.08	0.0003	0.01371
Stxbp5l	6362.1	-0.08	0.003	0.05866
Apc	48698.4	-0.08	0.0005	0.02051
Kcnb1	8632.6	-0.08	0.0019	0.04273
Itpr1	5796.6	-0.08	0.0032	0.06006
Speg	6065.5	-0.08	0.0006	0.02266
Csmd1	4507.3	-0.08	0.0047	0.07795
Abl1	2509.4	-0.08	0.0054	0.0846
Ndst1	9962.1	-0.08	0.0004	0.01762
Sc5d	4314.7	-0.08	0.0016	0.03982
Cacna1a	8379.2	-0.08	0.0012	0.0337
Coro2a	2448.9	-0.08	0.0053	0.08333
Gm996	5864.0	-0.08	0.0017	0.04161
Lrrc8b	3965.0	-0.08	0.005	0.08077
Pnpla6	2668.7	-0.08	0.0043	0.07394
Mta3	2263.2	-0.08	0.0066	0.09584
Celf5	16365.5	-0.08	1.79E-05	0.0024
Plbd2	2618.5	-0.08	0.006	0.0908
Herc1	12713.4	-0.08	0.0015	0.0383
Eml6	3148.9	-0.08	0.0041	0.07084
Fam117b	5412.1	-0.08	0.0008	0.02801
Zcchc14	5776.7	-0.08	0.0005	0.01958
Shisa6	9089.9	-0.08	0.0017	0.04049
Strn	2944.7	-0.08	0.0054	0.08456
Ppm1f	2820.6	-0.08	0.0062	0.09117
Adrbk2	6909.8	-0.08	0.0011	0.03245
Ptk2b	13604.8	-0.08	0.0011	0.03321
Cacna1i	5473.9	-0.08	0.0016	0.03982
Nfasc	10594.7	-0.08	0.0002	0.01082
Kcnq2	8867.7	-0.08	0.0004	0.01802
Ube2o	13535.0	-0.08	3.53E-05	0.00353
Rapgef5	3831.5	-0.08	0.0068	0.09659
Exoc4	6641.2	-0.08	0.002	0.04538
Rap1gap	8719.4	-0.08	0.0002	0.01057
Tubb4b	9484.4	-0.08	0.0003	0.01371
Syt3	3353.1	-0.08	0.003	0.05822
Dlg5	4305.9	-0.08	0.0019	0.04273
Slc12a5	25612.0	-0.08	3.12E-05	0.00332
Atp2b2	21852.7	-0.08	0.001	0.03067
6330403A02Rik	16757.1	-0.08	9.29E-05	0.00684
Snph	16604.6	-0.08	0.0001	0.00942
Shank3	15965.9	-0.08	8.84E-05	0.00656
Shroom2	6955.1	-0.08	0.0013	0.03517
Srrm2	18686.3	-0.08	0.0018	0.04234
Nktr	4385.4	-0.08	0.0068	0.09659
Nr3c2	3951.6	-0.08	0.0036	0.06502
Kdm4b	5878.0	-0.08	0.0008	0.02725
Cdkn1a	5878.3	-0.08	0.0016	0.03982
Mllt6	4463.2	-0.07	0.0023	0.05006
Wipf3	6851.1	-0.07	0.0031	0.05926
Acaca	6057.2	-0.07	0.002	0.04538
Lmtk3	18169.4	-0.07	0.0002	0.0134
Ogt	11393.7	-0.07	0.0019	0.04273

Gene	BaseMean	log_2FC	*p* value	$p_{adj(BH)}$
Prrt3	3377.7	-0.07	0.0044	0.07509
Ralgps2	4788.6	-0.07	0.0025	0.05163
Robo1	3884.6	-0.07	0.0069	0.09685
Slc2a13	4891.2	-0.07	0.006	0.09046
Dapk1	6067.0	-0.07	0.0014	0.037
Shank1	19249.8	-0.07	0.0012	0.03434
Srgap3	25502.6	-0.07	0.0003	0.01399
Lgi2	5702.1	-0.07	0.0068	0.09659
Aacs	9043.2	-0.07	0.0041	0.07147
Sez6l	20628.9	-0.07	0.0001	0.00827
Ccng1	10297.6	-0.07	0.0034	0.06264
Lhx6	4832.4	-0.07	0.0038	0.06719
Map1a	62178.0	-0.07	0.0009	0.02893
Plxna2	13504.9	-0.07	0.0027	0.0549
Mau2	5749.3	-0.07	0.0019	0.04363
Sun1	3312.1	-0.07	0.0052	0.08286
Kif1a	108509.6	-0.07	0.0004	0.01697
Srebf2	18204.3	-0.07	0.0014	0.03787
Ablim2	3196.6	-0.07	0.0072	0.09844
Exd2	3484.4	-0.07	0.0066	0.09549
Pygb	11318.8	-0.07	0.0015	0.03837
Rapgef2	9696.0	-0.07	0.0005	0.0193
Apc2	16220.6	-0.07	0.0019	0.04367
Itsn1	13046.3	-0.07	0.0002	0.0134
Arhgap21	10321.0	-0.07	0.0022	0.04828
Herc2	20330.8	-0.07	0.0061	0.09093
Lzts3	4890.0	-0.07	0.0062	0.09117
Scrt1	7701.9	-0.07	0.0008	0.02725
Zfp385a	3354.6	-0.07	0.0071	0.09784
Ewsr1	6660.5	-0.07	0.002	0.04469
Timp2	35473.3	-0.07	2.96E-05	0.00325
Brinp2	6926.3	-0.07	0.0055	0.08548
Zswim8	8431.5	-0.07	0.0014	0.03748
Cep170b	18322.6	-0.07	0.0017	0.04131
Rab11fip3	5503.1	-0.07	0.0028	0.05557
Cttnbp2	6278.3	-0.07	0.0038	0.06719
Tmod2	37417.3	-0.07	0.0016	0.03982
Wnt7b	5165.5	-0.07	0.0023	0.05006
Tub	13112.2	-0.07	0.0009	0.02964
Srrm4	4001.9	-0.07	0.0068	0.09659
Spen	4709.6	-0.07	0.0073	0.09934
Fbxo41	13214.1	-0.07	0.0005	0.01917
Zmym3	7905.2	-0.07	0.0032	0.06007
Edc4	4028.5	-0.07	0.0067	0.09631
Kctd12	9139.8	-0.07	0.0011	0.03333
Kif5a	87030.2	-0.07	0.0003	0.01405
Dopey2	5700.4	-0.07	0.0026	0.0526
Asb1	4651.1	-0.07	0.0036	0.06475
Ctif	5201.4	-0.07	0.0053	0.08333
Chd3	40836.9	-0.07	0.0002	0.01007
Tef	11888.7	-0.07	0.0011	0.03321
Rgl1	4550.3	-0.07	0.0047	0.0784
Sh3glb2	5210.8	-0.07	0.0047	0.0784
Tbc1d24	10937.8	-0.07	0.0047	0.0783
Plxna1	21530.3	-0.07	0.001	0.03068
Gabrb2	19264.0	-0.07	0.0032	0.06006
Ablim1	4356.8	-0.07	0.0072	0.09865
Shisa7	5790.1	-0.07	0.0047	0.0783
Cdh2	7722.2	-0.07	0.0021	0.04654
Ppip5k1	5281.8	-0.07	0.0049	0.0796
Cntnap1	12679.1	-0.07	0.0007	0.0246
Cacna1b	9989.3	-0.07	0.0027	0.0549
Agrn	9423.1	-0.07	0.007	0.09755
Sptb	8010.2	-0.07	0.0059	0.08959
Mroh1	5134.6	-0.07	0.0067	0.09584
Abat	16249.4	-0.07	0.0018	0.04255
Cacnb3	8453.8	-0.07	0.0025	0.05117
Sphkap	9225.5	-0.07	0.0045	0.0759
Wnk2	8888.2	-0.07	0.0042	0.07268
Nsmf	30989.1	-0.07	0.0009	0.03022
Slc17a7	76971.9	-0.06	0.0004	0.01891
Ddx17	23724.7	-0.06	0.0024	0.05117
Camk2a	72791.5	-0.06	0.0016	0.03982
Fam120a	8162.0	-0.06	0.0072	0.09881
L1cam	21587.5	-0.06	0.001	0.03079
Cpeb4	8692.8	-0.06	0.006	0.09093
Atp2b3	9571.9	-0.06	0.0036	0.06484
Dnm1	42164.2	-0.06	0.0006	0.02297
Megf8	12264.1	-0.06	0.0021	0.04654
Islr2	12594.7	-0.06	0.0013	0.03552
Nisch	28328.9	-0.06	0.0004	0.01732
Lrrn2	10904.9	-0.06	0.0017	0.04063
Sptbn1	68342.7	-0.06	0.0021	0.04568
Syt7	11032.7	-0.06	0.0021	0.04567
Jph4	24869.2	-0.06	0.0004	0.01886
Kcnq5	6856.5	-0.06	0.0061	0.09093
Tmcc2	14634.3	-0.06	0.0006	0.02378
Tsc2	8105.6	-0.06	0.0052	0.08286
Syn2	51719.2	-0.06	0.0038	0.06731
Tiam1	7287.4	-0.06	0.0066	0.09521
Aatk	13094.9	-0.06	0.0039	0.06781
Ndrg4	95027.5	-0.06	0.0008	0.02789
Cplx1	36720.2	-0.06	0.0029	0.05792
Slc25a23	17471.7	-0.06	0.0013	0.03493
Camk2b	49942.7	-0.06	0.0003	0.01655
Tro	9913.8	-0.06	0.0061	0.09117
Olfm1	43278.4	-0.06	0.0018	0.04198
Sez6	19131.0	-0.06	0.0019	0.04297
Dlgap3	19355.7	-0.06	0.0011	0.03321
Nfix	24987.1	-0.06	0.0044	0.07475
Mcf2l	7462.3	-0.06	0.0074	0.09934
Tubb4a	25399.2	-0.06	0.0069	0.09681
Spag9	15932.3	-0.06	0.0074	0.09951
Rap1gap2	12176.6	-0.06	0.0053	0.08333
Gria1	44975.3	-0.05	0.0018	0.04273
Tmem63b	11153.7	-0.05	0.007	0.09755
Arpp21	14606.7	-0.05	0.0032	0.06035
Pde2a	25710.7	-0.05	0.0016	0.03982
Sptbn2	73531.2	-0.05	0.0013	0.03544
Chd5	21000.4	-0.05	0.0051	0.08208
Sez6l2	19450.0	-0.05	0.0037	0.06593
Fam115a	13213.3	-0.05	0.0065	0.0948
Mapk8ip2	16233.3	-0.05	0.0057	0.0873
Hpcal4	28066.8	-0.05	0.0054	0.0846
Kidins220	25230.3	-0.05	0.005	0.08077
Clstn1	68392.2	-0.05	0.0018	0.04234
Nsf	60003.9	-0.05	0.0064	0.09411
Cadm1	26061.8	-0.04	0.0068	0.09659

Table S8. Overrepresented (p_{adj} ≤ 0.05) Gene Ontology categories among upregulated genes in *Lnc473*-expressing mouse neurons (goseq results).

GO cat.	NumDEInCat	NumInCat	%DEinCat	Term	Ontology	p_{Holm}
GO:0006518	66	719	9.18	peptide metabolic process	BP	3.59E-27
GO:0006412	57	554	10.29	translation	BP	2.12E-25
GO:0043043	58	576	10.07	peptide biosynthetic process	BP	2.19E-25
GO:0043603	69	934	7.39	cellular amide metabolic process	BP	1.06E-22
GO:0043604	59	683	8.64	amide biosynthetic process	BP	3.32E-22
GO:0022626	23	94	24.47	cytosolic ribosome	CC	6.01E-18
GO:1901566	75	1354	5.54	organonitrogen compound biosynthetic process	BP	3.51E-17
GO:0003735	25	145	17.24	structural constituent of ribosome	MF	9.50E-16
GO:0044391	26	173	15.03	ribosomal subunit	CC	8.16E-15
GO:0005840	27	204	13.24	ribosome	CC	7.46E-14
GO:1990904	46	683	6.73	ribonucleoprotein complex	CC	4.65E-13
GO:0003723	54	1049	5.15	RNA binding	MF	1.39E-09
GO:0022625	13	50	26.00	cytosolic large ribosomal subunit	CC	3.15E-09
GO:0045182	19	124	15.32	translation regulator activity	MF	6.25E-09
GO:0005829	107	3368	3.18	cytosol	CC	3.36E-08
GO:0015934	16	108	14.81	large ribosomal subunit	CC	8.47E-08
GO:0032991	133	4804	2.77	protein-containing complex	CC	1.19E-07
GO:0022627	11	40	27.50	cytosolic small ribosomal subunit	CC	1.22E-07
GO:0022613	29	383	7.57	ribonucleoprotein complex biogenesis	BP	1.47E-07
GO:0015935	13	69	18.84	small ribosomal subunit	CC	3.92E-07
GO:0006418	11	39	28.21	tRNA aminoacylation for protein translation	BP	9.50E-07
GO:0090079	15	93	16.13	translation regulator activity, nucleic acid binding	MF	9.91E-07
GO:0002181	14	85	16.47	cytoplasmic translation	BP	1.11E-06
GO:0006520	22	231	9.52	cellular amino acid metabolic process	BP	1.13E-06
GO:0005737	218	10063	2.17	cytoplasm	CC	1.25E-06
GO:0004812	11	40	27.50	aminoacyl-tRNA ligase activity	MF	1.29E-06
GO:0016875	11	40	27.50	ligase activity, forming carbon-oxygen bonds	MF	1.29E-06
GO:0043039	11	41	26.83	tRNA aminoacylation	BP	1.65E-06
GO:0051082	14	83	16.87	unfolded protein binding	MF	1.80E-06
GO:0042254	23	270	8.52	ribosome biogenesis	BP	1.88E-06
GO:1901564	140	5351	2.62	organonitrogen compound metabolic process	BP	2.18E-06
GO:0043038	11	42	26.19	amino acid activation	BP	2.20E-06
GO:0006413	15	101	14.85	translational initiation	BP	3.14E-06
GO:0008135	13	74	17.57	translation factor activity, RNA binding	MF	7.31E-06
GO:0019843	12	66	18.18	rRNA binding	MF	7.37E-06
GO:0044267	114	4088	2.79	cellular protein metabolic process	BP	1.51E-05
GO:0034248	26	380	6.84	regulation of cellular amide metabolic process	BP	2.48E-05
GO:0005622	247	12396	1.99	intracellular anatomical structure	CC	2.51E-05
GO:0034641	135	5270	2.56	cellular nitrogen compound metabolic process	BP	3.72E-05
GO:0009058	129	4955	2.60	biosynthetic process	BP	4.53E-05
GO:0019538	122	4595	2.66	protein metabolic process	BP	4.91E-05
GO:1901576	127	4869	2.61	organic substance biosynthetic process	BP	6.09E-05
GO:0006457	16	149	10.74	protein folding	BP	6.59E-05
GO:0044249	125	4802	2.60	cellular biosynthetic process	BP	0.000104
GO:0003743	10	47	21.28	translation initiation factor activity	MF	0.000113
GO:0009059	110	4022	2.73	macromolecule biosynthetic process	BP	0.000121
GO:0044271	108	3934	2.75	cellular nitrogen compound biosynthetic process	BP	0.000131
GO:0034660	26	413	6.30	ncRNA metabolic process	BP	0.000141
GO:0034645	108	3987	2.71	cellular macromolecule biosynthetic process	BP	0.000311
GO:0006417	22	325	6.77	regulation of translation	BP	0.00067
GO:0006807	178	8065	2.21	nitrogen compound metabolic process	BP	0.001302
GO:0010467	125	5074	2.46	gene expression	BP	0.001618
GO:0042273	10	65	15.38	ribosomal large subunit biogenesis	BP	0.001831
GO:0001732	4	4	100.00	formation of cytoplasmic translation initiation complex	BP	0.002162
GO:0003729	20	290	6.90	mRNA binding	MF	0.002272
GO:0005198	27	538	5.02	structural molecule activity	MF	0.00309
GO:0045202	50	1387	3.60	synapse	CC	0.00539
GO:0140101	12	109	11.01	catalytic activity, acting on a tRNA	MF	0.007193
GO:0016874	14	152	9.21	ligase activity	MF	0.008562
GO:0044085	76	2663	2.85	cellular component biogenesis	BP	0.012715
GO:0044238	182	8541	2.13	primary metabolic process	BP	0.012787
GO:0008152	198	9611	2.06	metabolic process	BP	0.013424
GO:0044237	185	8743	2.12	cellular metabolic process	BP	0.014219
GO:0006364	15	194	7.73	rRNA processing	BP	0.015798
GO:0043231	198	9563	2.07	intracellular membrane-bounded organelle	CC	0.017884
GO:0016072	15	201	7.46	rRNA metabolic process	BP	0.025263
GO:0043227	207	10189	2.03	membrane-bounded organelle	CC	0.025361

Table S9. Overrepresented (p_{adj} ≤ 0.05) Gene Ontology categories among downregulated genes in *Lnc473*-expressing mouse neurons (goseq results).

GO cat.	NumDEInCat	NumInCat	%DEinCat	Term	Ontology	p_{Holm}
GO:0036477	93	1017	9.14	somatodendritic compartment	CC	1.96E-14
GO:0043005	115	1464	7.86	neuron projection	CC	3.09E-14
GO:0071944	216	4819	4.48	cell periphery	CC	6.26E-14
GO:0005886	197	4379	4.50	plasma membrane	CC	2.75E-12
GO:0099537	69	770	8.96	trans-synaptic signaling	BP	6.47E-12
GO:0007268	68	761	8.94	chemical synaptic transmission	BP	8.99E-12
GO:0098916	68	761	8.94	anterograde trans-synaptic signaling	BP	8.99E-12
GO:0120025	135	2169	6.22	plasma membrane bounded cell projection	CC	1.72E-11
GO:0030425	72	702	10.26	dendrite	CC	1.86E-11
GO:0097447	72	705	10.21	dendritic tree	CC	2.01E-11
GO:0099536	69	798	8.65	synaptic signaling	BP	5.14E-11
GO:0042995	141	2383	5.92	cell projection	CC	1.34E-10
GO:0023052	209	5109	4.09	signaling	BP	7.53E-10
GO:0043025	66	710	9.30	neuronal cell body	CC	1.95E-09
GO:0048699	111	1630	6.81	generation of neurons	BP	3.97E-09
GO:0022008	116	1751	6.62	neurogenesis	BP	4.06E-09
GO:0007154	208	5160	4.03	cell communication	BP	4.25E-09
GO:0007399	137	2288	5.99	nervous system development	BP	5.75E-09
GO:0045202	99	1387	7.14	synapse	CC	7.90E-09
GO:0098978	55	493	11.16	glutamatergic synapse	CC	8.88E-09
GO:0044297	68	802	8.48	cell body	CC	1.10E-08
GO:0098794	67	712	9.41	postsynapse	CC	1.38E-08
GO:0007267	94	1536	6.12	cell-cell signaling	BP	1.49E-08
GO:0031175	86	1082	7.95	neuron projection development	BP	7.53E-08
GO:0030182	101	1473	6.86	neuron differentiation	BP	1.03E-07
GO:0050804	50	572	8.74	modulation of chemical synaptic transmission	BP	1.32E-07
GO:0099177	50	573	8.73	regulation of trans-synaptic signaling	BP	1.34E-07
GO:0048666	91	1226	7.42	neuron development	BP	2.63E-07
GO:0000904	70	786	8.91	cell morphogenesis involved in differentiation	BP	6.96E-07
GO:0120039	66	694	9.51	plasma membrane bounded cell projection morphogenesis	BP	9.19E-07
GO:0032989	71	811	8.75	cellular component morphogenesis	BP	1.10E-06
GO:0016020	249	7354	3.39	membrane	CC	1.11E-06
GO:0048858	66	699	9.44	cell projection morphogenesis	BP	1.17E-06
GO:0048812	65	680	9.56	neuron projection morphogenesis	BP	1.37E-06
GO:0048667	61	628	9.71	cell morphogenesis involved in neuron differentiation	BP	1.72E-06
GO:0030424	61	698	8.74	axon	CC	2.33E-06
GO:0006812	66	1060	6.23	cation transport	BP	2.38E-06
GO:0032990	66	718	9.19	cell part morphogenesis	BP	2.43E-06
GO:0000902	82	1069	7.67	cell morphogenesis	BP	3.83E-06
GO:0030054	115	1954	5.89	cell junction	CC	4.53E-06
GO:0034703	29	210	13.81	cation channel complex	CC	9.90E-06
GO:0048468	119	2178	5.46	cell development	BP	1.01E-05
GO:0097060	46	451	10.20	synaptic membrane	CC	1.12E-05
GO:0098793	49	564	8.69	presynapse	CC	1.34E-05
GO:0050808	48	468	10.26	synapse organization	BP	1.42E-05
GO:0042734	29	191	15.18	presynaptic membrane	CC	1.42E-05
GO:0065008	159	3765	4.22	regulation of biological quality	BP	1.85E-05
GO:0120036	97	1507	6.44	plasma membrane bounded cell projection organization	BP	2.01E-05
GO:0030030	98	1547	6.33	cell projection organization	BP	2.03E-05
GO:0034330	60	708	8.47	cell junction organization	BP	2.64E-05
GO:0034702	32	280	11.43	ion channel complex	CC	4.46E-05
GO:0099572	44	420	10.48	postsynaptic specialization	CC	6.03E-05
GO:0098655	50	711	7.03	cation transmembrane transport	BP	7.52E-05
GO:0032879	124	2647	4.68	regulation of localization	BP	7.66E-05
GO:0051049	90	1739	5.18	regulation of transport	BP	0.0001042
GO:1902495	32	296	10.81	transmembrane transporter complex	CC	0.000106
GO:0098984	43	414	10.39	neuron to neuron synapse	CC	0.0002569
GO:0098590	74	1176	6.29	plasma membrane region	CC	0.0002647
GO:1990351	32	309	10.36	transporter complex	CC	0.0002822
GO:0014069	41	384	10.68	postsynaptic density	CC	0.0003055
GO:0048167	28	294	9.52	regulation of synaptic plasticity	BP	0.0003912
GO:0032279	41	389	10.54	asymmetric synapse	CC	0.0004666
GO:0051960	43	519	8.29	regulation of nervous system development	BP	0.0007457
GO:0007610	51	705	7.23	behavior	BP	0.000862
GO:0061564	45	506	8.89	axon development	BP	0.0011398
GO:0050877	64	1215	5.27	nervous system process	BP	0.001636
GO:0007611	30	292	10.27	learning or memory	BP	0.0016539
GO:0023051	132	3116	4.24	regulation of signaling	BP	0.0024905
GO:0007409	43	467	9.21	axonogenesis	BP	0.0025804
GO:0016021	146	4301	3.39	integral component of membrane	CC	0.0031299
GO:0031224	149	4451	3.35	intrinsic component of membrane	CC	0.0031726
GO:0010646	131	3104	4.22	regulation of cell communication	BP	0.0036424
GO:0044304	17	80	21.25	main axon	CC	0.0047578
GO:0051128	111	2324	4.78	regulation of cellular component organization	BP	0.0065859
GO:0048731	176	4412	3.99	system development	BP	0.0077835
GO:0035254	14	64	21.88	glutamate receptor binding	MF	0.0078622
GO:0007165	168	4598	3.65	signal transduction	BP	0.0083725
GO:0099601	13	64	20.31	regulation of neurotransmitter receptor activity	BP	0.0094145
GO:0005216	32	396	8.08	ion channel activity	MF	0.0103586
GO:0005261	28	310	9.03	cation channel activity	MF	0.0114291
GO:0090066	39	544	7.17	regulation of anatomical structure size	BP	0.0118585
GO:0050890	30	325	9.23	cognition	BP	0.012803
GO:0032501	225	6604	3.41	multicellular organismal process	BP	0.0136155
GO:0032535	33	394	8.38	regulation of cellular component size	BP	0.0139152
GO:0098662	41	628	6.53	inorganic cation transmembrane transport	BP	0.0144813
GO:0010975	44	534	8.24	regulation of neuron projection development	BP	0.0164753
GO:0022890	37	543	6.81	inorganic cation transmembrane transporter activity	MF	0.0167943
GO:0050767	36	427	8.43	regulation of neurogenesis	BP	0.0190883
GO:0043269	67	1297	5.17	regulation of ion transport	BP	0.0199906
GO:0098660	43	683	6.30	inorganic ion transmembrane transport	BP	0.0201309
GO:0015267	32	436	7.34	channel activity	MF	0.0258625
GO:0022803	32	436	7.34	passive transmembrane transporter activity	MF	0.0258625
GO:0060560	28	267	10.49	developmental growth involved in morphogenesis	BP	0.0278352
GO:0009653	122	2633	4.63	anatomical structure morphogenesis	BP	0.0443481
GO:0099056	17	112	15.18	integral component of presynaptic membrane	CC	0.0457006
GO:0008361	23	210	10.95	regulation of cell size	BP	0.0470224

Table S10. Overrepresented ($p_{adj} \leq 0.05$) Human Phenotype Ontology categories among downregulated genes in *Lnc473*-expressing mouse neurons (g:Profiler results).

HP cat.	NumDEInCat	NumInCat	%DEinCat	Term	p_{Bonf}
HP:0002133	29	164	17.68	Status epilepticus	1.13E-10
HP:0007359	34	252	13.49	Focal-onset seizure	1.96E-09
HP:0011146	29	185	15.68	Dialeptic seizure	2.91E-09
HP:0006919	36	291	12.37	Abnormal aggressive, impulsive or violent behavior	5.12E-09
HP:0002069	31	248	12.50	Bilateral tonic-clonic seizure	1.99E-07
HP:0033259	25	162	15.43	Non-motor seizure	2.00E-07
HP:0002121	24	149	16.11	Generalized non-motor (absence) seizure	2.01E-07
HP:0002384	19	94	20.21	Focal impaired awareness seizure	4.36E-07
HP:0020219	32	280	11.43	Motor seizure	1E-06
HP:0001336	33	308	10.71	Myoclonus	2.8E-06
HP:0032794	23	155	14.84	Myoclonic seizure	2.92E-06
HP:0011182	30	260	11.54	Interictal epileptiform activity	3.24E-06
HP:0025373	30	260	11.54	Interictal EEG abnormality	3.24E-06
HP:0003808	93	1730	5.38	Abnormal muscle tone	3.44E-06
HP:0002197	32	294	10.88	Generalized-onset seizure	3.6E-06
HP:0011153	17	83	20.48	Focal motor seizure	3.62E-06
HP:0002342	26	209	12.44	Intellectual disability, moderate	1.04E-05
HP:0000729	42	497	8.45	Autistic behavior	1.45E-05
HP:0002020	34	358	9.50	Gastroesophageal reflux	3.65E-05
HP:0001250	86	1596	5.39	Seizure	4.24E-05
HP:0000718	24	193	12.44	Aggressive behavior	4.68E-05
HP:0032677	22	164	13.41	Generalized-onset motor seizure	4.99E-05
HP:0011198	26	225	11.56	EEG with generalized epileptiform discharges	5.12E-05
HP:0002353	38	445	8.54	EEG abnormality	7.8E-05
HP:0004305	60	928	6.47	Involuntary movements	7.95E-05
HP:0032792	15	78	19.23	Tonic seizure	9.35E-05
HP:0030178	39	468	8.33	Abnormality of central nervous system electrophysiology	9.54E-05
HP:0001249	86	1624	5.30	Intellectual disability	0.000111
HP:0025100	13	58	22.41	Abnormal hippocampus morphology	0.000122
HP:0002123	19	130	14.62	Generalized myoclonic seizure	0.000132
HP:0010818	14	69	20.29	Generalized tonic seizure	0.000137
HP:0007343	13	59	22.03	Abnormal morphology of the limbic system	0.000152
HP:0100716	20	147	13.61	Self-injurious behavior	0.000193
HP:0000733	24	209	11.48	Stereotypy	0.000228
HP:0011442	85	1618	5.25	Abnormal central motor function	0.000238
HP:0200134	16	96	16.67	Epileptic encephalopathy	0.000263
HP:0012638	126	3008	4.19	Abnormal nervous system physiology	0.000273
HP:0100851	39	491	7.94	Abnormal emotion/affect behavior	0.000364
HP:0011804	99	2055	4.82	Abnormal muscle physiology	0.000384
HP:0001311	41	547	7.50	Abnormal nervous system electrophysiology	0.000788
HP:0012433	20	164	12.20	Abnormal social behavior	0.001229
HP:0001288	58	947	6.12	Gait disturbance	0.001237
HP:0100022	87	1730	5.03	Abnormality of movement	0.001316
HP:0001252	78	1482	5.26	Hypotonia	0.001775
HP:0000736	31	370	8.38	Short attention span	0.003385
HP:0002266	7	18	38.89	Focal clonic seizure	0.003521
HP:0011185	16	117	13.68	EEG with focal epileptiform discharges	0.004374
HP:0012759	104	2300	4.52	Neurodevelopmental abnormality	0.004419
HP:0025270	43	627	6.86	Abnormality of esophagus physiology	0.004508
HP:0001256	29	337	8.61	Intellectual disability, mild	0.004743
HP:0011446	103	2279	4.52	Abnormality of higher mental function	0.006119
HP:0005324	8	27	29.63	Disturbance of facial expression	0.006215
HP:0002493	63	1119	5.63	Upper motor neuron dysfunction	0.006427
HP:0032679	11	57	19.30	Focal non-motor seizure	0.007307
HP:0001270	42	616	6.82	Motor delay	0.007428
HP:0003011	105	2356	4.46	Abnormality of the musculature	0.008308
HP:0001276	54	902	5.99	Hypertonia	0.008958
HP:0010819	13	83	15.66	Atonic seizure	0.009974
HP:0000734	19	172	11.05	Disinhibition	0.011518
HP:0000717	18	159	11.32	Autism	0.015089
HP:0000707	128	3225	3.97	Abnormality of the nervous system	0.01539
HP:0000750	47	753	6.24	Delayed speech and language development	0.018368
HP:0001290	54	924	5.84	Generalized hypotonia	0.019494
HP:0000752	32	422	7.58	Hyperactivity	0.019797
HP:0002463	48	781	6.15	Language impairment	0.021581
HP:0007334	7	23	30.43	Bilateral tonic-clonic seizure with focal onset	0.023563
HP:0012758	88	1863	4.72	Neurodevelopmental delay	0.025122
HP:0100710	17	150	11.33	Impulsivity	0.028196
HP:0002317	16	136	11.76	Unsteady gait	0.032908
HP:0002311	20	203	9.85	Incoordination	0.036629
HP:0012179	12	80	15.00	Craniofacial dystonia	0.03916
HP:0001268	25	296	8.45	Mental deterioration	0.041002
HP:0007270	7	25	28.00	Atypical absence seizure	0.043673

Table S11. Overrepresented (*Z*-score > 2) conserved transcription factor (TF) binding sites (TFBS) in the promoters of upregulated (± 2 kb of TSS) genes in *Lnc473*-expressing mouse neurons (oPOSSUM3 results).

TF	JASPAR ID	Target gene hits	Target gene non-hits	Target proportion	Enrichment over backg.	Backg. gene hits	Backg. gene non-hits	Backg. proportion	Target TFBS hits	Target TFBS nucleotide rate	Backg. TFBS hits	Backg. TFBS nucleotide rate	TFBS enrichment	Z-score	Fisher score
SRF	MA0083.1	4	274	0.01	3.65	68	17204	0.00	6	5.52E-04	70	9.84E-05	5.61	16.36	3.60
Nkx2-5	MA0063.1	126	152	0.45	1.14	6854	10418	0.40	350	1.88E-02	19188	1.57E-02	1.20	8.79	3.41
ARID3A	MA0151.1	109	169	0.39	1.08	6245	11027	0.36	303	1.39E-02	16489	1.16E-02	1.20	7.87	1.82
Ar	MA0007.1	1	277	0.00	4.44	14	17258	0.00	1	1.69E-04	14	3.61E-05	4.68	7.74	1.55
ELF5	MA0136.1	110	168	0.40	1.18	5814	11458	0.34	192	1.32E-02	10553	1.11E-02	1.19	7.25	3.75
Stat3	MA0144.1	35	243	0.13	1.37	1589	15683	0.09	40	3.06E-03	1827	2.14E-03	1.43	7.19	3.27
CEBPA	MA0102.2	45	233	0.16	1.07	2601	14671	0.15	73	5.03E-03	3610	3.81E-03	1.32	7.17	1.12
Mycn	MA0104.2	35	243	0.13	1.39	1560	15712	0.09	40	3.06E-03	1836	2.15E-03	1.42	7.09	3.50
Prrx2	MA0075.1	106	172	0.38	1.09	6063	11209	0.35	296	1.13E-02	16315	9.56E-03	1.18	6.59	1.82
IRF1	MA0050.1	14	264	0.05	1.53	567	16705	0.03	15	1.38E-03	614	8.63E-04	1.60	6.30	2.53
IRF2	MA0051.1	1	277	0.00	3.88	16	17256	0.00	1	1.38E-04	16	3.38E-05	4.08	6.24	1.44
CREB1	MA0018.2	39	239	0.14	1.28	1892	15380	0.11	45	2.76E-03	2209	2.07E-03	1.33	5.43	2.71
Sox5	MA0087.1	53	225	0.19	1.15	2855	14417	0.17	81	4.34E-03	4251	3.49E-03	1.24	5.23	1.91
Pdx1	MA0132.1	116	162	0.42	1.07	6757	10515	0.39	332	1.53E-02	19404	1.36E-02	1.13	5.03	1.58
SRY	MA0084.1	73	205	0.26	1.09	4174	13098	0.24	134	9.24E-03	7584	8.00E-03	1.16	5.02	1.48
NFATC2	MA0152.1	101	177	0.36	1.08	5808	11464	0.34	210	1.13E-02	12129	9.95E-03	1.14	4.77	1.67
Myc	MA0147.1	26	252	0.09	1.23	1309	15963	0.08	30	2.30E-03	1502	1.76E-03	1.31	4.61	1.83
GABPA	MA0062.2	33	245	0.12	1.16	1763	15509	0.10	39	3.29E-03	2080	2.68E-03	1.23	4.20	1.58
Nobox	MA0125.1	59	219	0.21	1.12	3272	14000	0.19	98	6.01E-03	5520	5.18E-03	1.16	4.17	1.68
TBP	MA0108.2	24	254	0.09	1.16	1289	15983	0.07	27	3.10E-03	1464	2.57E-03	1.21	3.75	1.34
RORA_1	MA0071.1	24	254	0.09	1.12	1337	15935	0.08	28	2.15E-03	1486	1.74E-03	1.24	3.47	1.14
FEV	MA0156.1	72	206	0.26	1.07	4179	13093	0.24	106	6.50E-03	6210	5.82E-03	1.12	3.19	1.29
Nr2e3	MA0164.1	33	245	0.12	1.17	1755	15517	0.10	40	2.15E-03	2188	1.79E-03	1.20	2.96	1.61
STAT1	MA0137.2	12	266	0.04	1.22	610	16662	0.04	12	1.38E-03	629	1.11E-03	1.24	2.93	1.26
ELK4	MA0076.1	15	263	0.05	1.15	809	16463	0.05	17	1.17E-03	881	9.29E-04	1.26	2.84	1.12
Esrrb	MA0141.1	29	249	0.10	1.15	1569	15703	0.09	32	2.94E-03	1814	2.55E-03	1.15	2.77	1.40
SPI1	MA0080.2	130	148	0.47	1.07	7582	9690	0.44	287	1.54E-02	17753	1.46E-02	1.05	2.49	1.68
Gata1	MA0035.2	58	220	0.21	1.07	3373	13899	0.20	84	7.08E-03	5107	6.58E-03	1.08	2.20	1.17
MEF2A	MA0052.1	19	259	0.07	1.13	1047	16225	0.06	21	1.61E-03	1182	1.39E-03	1.16	2.14	1.11
Zfx	MA0146.1	45	233	0.16	1.16	2400	14872	0.14	54	5.79E-03	3279	5.38E-03	1.08	2.02	1.85

Table S12. Overrepresented (*Z*-score > 2) conserved transcription factor (TF) binding sites (TFBS) in the promoters of downregulated (± 2 kb of TSS) genes in *Lnc473*-expressing mouse neurons (oPOSSUM3 results).

TF	JASPAR ID	Target gene hits	Target gene non-hits	Target propotion	Enrichment over backg.	Backg. gene hits	Backg. gene non-hits	Backg. propotion	Target TFBS hits	Target TFBS nucleotide rate	Backg. TFBS hits	Backg. TFBS nucleotide rate	TFBS enrichment	Z-score	Fisher score
REST	MA0138.2	10	381	0.03	7.36	60	17212	0.00	11	6.47E-04	62	1.53E-04	4.23	23.86	12.70
MZF1_1-4	MA0056.1	286	105	0.73	1.52	8330	8942	0.48	1566	2.63E-02	30538	2.15E-02	1.22	20.00	51.79
SP1	MA0079.2	203	188	0.52	1.75	5129	12143	0.30	556	1.56E-02	11135	1.30E-02	1.20	13.31	43.67
Pax5	MA0014.1	3	388	0.01	7.80	17	17255	0.00	3	1.68E-04	17	3.98E-05	4.22	12.01	4.68
Esrrb	MA0141.1	81	310	0.21	2.28	1569	15703	0.09	105	3.53E-03	1814	2.55E-03	1.38	11.58	26.14
CTCF	MA0139.1	23	368	0.06	3.07	331	16941	0.02	23	1.22E-03	336	7.48E-04	1.63	10.38	12.33
ZNF354C	MA0130.1	301	90	0.77	1.43	9294	7978	0.54	1546	2.60E-02	33412	2.35E-02	1.11	9.84	46.78
MZF1_5-13	MA0057.1	162	229	0.41	1.75	4084	13188	0.24	365	1.02E-02	7453	8.73E-03	1.17	9.58	31.99
Klf4	MA0039.2	225	166	0.58	1.70	5858	11414	0.34	565	1.58E-02	12099	1.42E-02	1.11	8.34	47.14
PPARG::RXRA	MA0065.2	22	369	0.06	2.83	344	16928	0.02	23	9.67E-04	361	6.35E-04	1.52	7.84	10.64
EWSR1-FLI1	MA0149.1	2	389	0.01	5.89	15	17257	0.00	2	1.01E-04	15	3.16E-05	3.20	7.20	2.93
ZEB1	MA0103.1	263	128	0.67	1.51	7688	9584	0.45	843	1.42E-02	18327	1.29E-02	1.10	6.80	42.78
Zfp423	MA0116.1	23	368	0.06	2.06	494	16778	0.03	29	1.22E-03	526	9.25E-04	1.32	5.76	6.68
PLAG1	MA0163.1	6	385	0.02	2.88	92	17180	0.01	7	2.75E-04	94	1.54E-04	1.79	5.72	3.83
Sox2	MA0143.1	6	385	0.02	2.65	100	17172	0.01	7	2.94E-04	102	1.79E-04	1.64	5.06	3.49
TLX1::NFIC	MA0119.1	4	387	0.01	3.61	49	17223	0.00	4	1.57E-04	49	8.04E-05	1.95	5.01	3.52
Tcfcp2l1	MA0145.1	65	326	0.17	2.17	1324	15948	0.08	73	2.86E-03	1501	2.46E-03	1.16	4.82	18.93
RXR::RAR_DR5	MA0159.1	3	388	0.01	2.50	53	17219	0.00	4	1.91E-04	56	1.12E-04	1.71	4.39	2.06
PBX1	MA0070.1	8	383	0.02	2.49	142	17130	0.01	9	3.03E-04	146	2.05E-04	1.48	4.00	3.98
EBF1	MA0154.1	95	296	0.24	1.66	2525	14747	0.15	155	4.34E-03	3368	3.95E-03	1.10	3.76	14.64
Myf	MA0055.1	73	318	0.19	1.82	1776	15496	0.10	99	3.33E-03	2134	3.00E-03	1.11	3.56	14.28
NHLH1	MA0048.1	29	362	0.07	2.09	613	16659	0.04	32	1.08E-03	646	9.08E-04	1.19	3.29	8.35
RREB1	MA0073.1	4	387	0.01	2.64	67	17205	0.00	4	2.24E-04	67	1.57E-04	1.43	3.13	2.63
NR3C1	MA0113.1	4	387	0.01	2.64	67	17205	0.00	4	2.02E-04	67	1.41E-04	1.43	2.97	2.63
NR4A2	MA0160.1	149	242	0.38	1.86	3548	13724	0.21	225	5.04E-03	5017	4.70E-03	1.07	2.96	33.43
MEF2A	MA0052.1	47	344	0.12	1.98	1047	16225	0.06	56	1.57E-03	1182	1.39E-03	1.13	2.93	11.52
Egr1	MA0162.1	45	346	0.12	2.08	956	16316	0.06	50	1.54E-03	1064	1.37E-03	1.12	2.71	12.22
Arnt::Ahr	MA0006.1	201	190	0.51	1.78	4984	12288	0.29	428	7.20E-03	9723	6.84E-03	1.05	2.59	45.29
Mycn	MA0104.2	69	322	0.18	1.95	1560	15712	0.09	84	2.35E-03	1836	2.15E-03	1.09	2.59	16.11
MYC::MAX	MA0059.1	17	374	0.04	2.28	329	16943	0.02	17	5.24E-04	338	4.36E-04	1.20	2.49	6.26
CEBPA	MA0102.2	110	281	0.28	1.87	2601	14671	0.15	161	4.06E-03	3610	3.81E-03	1.07	2.44	23.70
Ar	MA0007.1	1	390	0.00	3.16	14	17258	0.00	1	6.16E-05	14	3.61E-05	1.71	2.40	1.25
RORA_2	MA0072.1	12	379	0.03	2.28	232	17040	0.01	12	4.71E-04	238	3.90E-04	1.21	2.38	4.78
HIF1A::ARNT	MA0259.1	186	205	0.48	1.74	4715	12557	0.27	359	8.05E-03	8234	7.72E-03	1.04	2.23	38.06
USF1	MA0093.1	72	319	0.18	2.05	1552	15720	0.09	84	1.65E-03	1838	1.51E-03	1.09	2.13	18.69
TAL1::TCF3	MA0091.1	35	356	0.09	2.14	721	16551	0.04	36	1.21E-03	778	1.09E-03	1.11	2.08	10.33

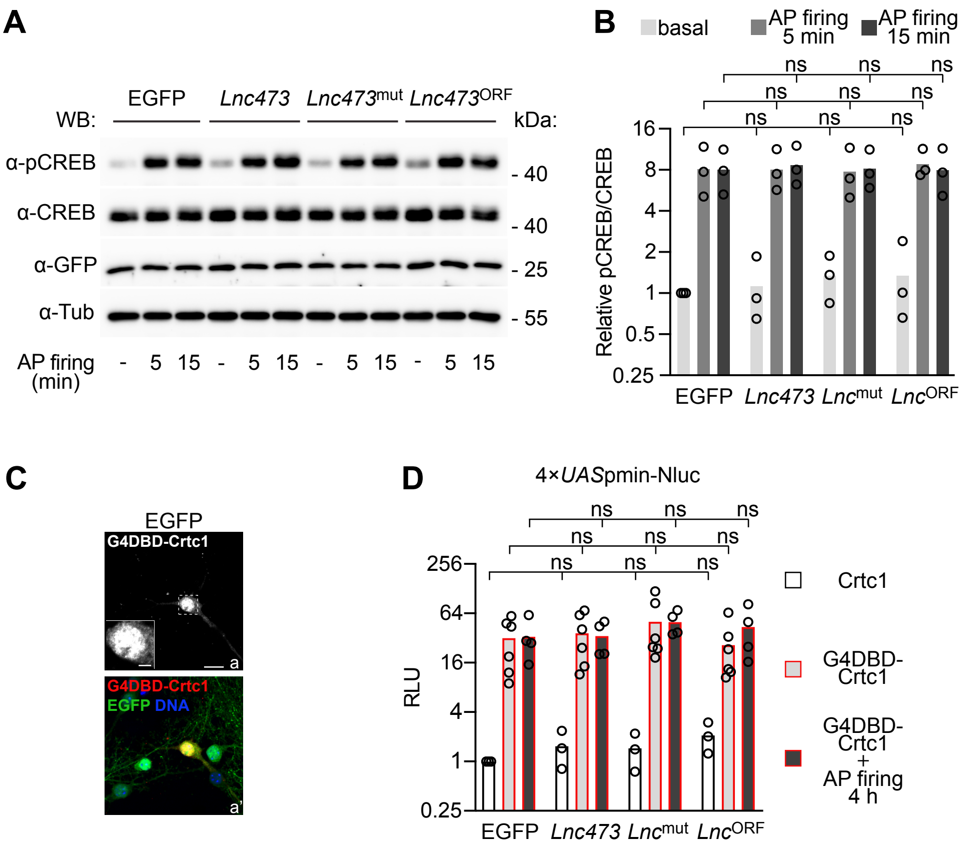

Figure S3. *Lnc473* RNA neither stimulates CREB phosphorylation nor Crtc1 transactivation capacity. Mouse primary neurons were infected with either the rAAV encoding wild-type human *Lnc473* and EGFP (*Lnc473*), the rAAV encoding *Lnc473* with mutated translational start codon of its ORF (UniProt A8K010) and EGFP (*Lnc473*^{mut}), the rAAV encoding *Lnc473* ORF (under control of p*SYN1*) and EGFP (*Lnc473*^{ORF}), or the rAAV encoding EGFP (EGFP). Where indicated AP firing was induced with Bic (50 μM). *A*, Representative western blot result of CREB phosphorylation at S133 (pCREB). Total CREB levels were analyzed for normalization. EGFP and Tubulin were analyzed for monitoring infection efficacy and sample loading, respectively. *B*, Quantification of *A*. pCREB levels were normalized to CREB levels and are presented relative to untreated EGFP-expressing control cells. *C*, Representative immunocytochemistry result of Gal4 DNA binding domain (G4DBD)-fused Crtc1 localization in neurons. rAAV/EGFP-infected neurons were transfected with a construct encoding G4DBD-Crtc1 tagged with HA. G4DBD-Crtc1 localization was monitored in basal conditions (shown) and after 1 h of AP firing, which displayed similar results. *D*, Results of a reporter assay for G4DBD-Crtc1-dependent transcription. Plasmids encoding Nluc under the control of four upstream activation sequences (UAS; G4DBD-binding *cis*-elements) and a minimal promoter (4×*UAS*pmin), and either Crtc1 or G4DBD-Crtc1, were transfected into neurons infected with the indicated rAAVs. Luminescence was measured in basal conditions and after 4 h of AP firing as indicated. RLUs are relative to the mean baseline levels of EGFP-expressing Crtc1-transfected control cells and normalized to firefly luciferase (FFluc) activity obtained with co-transfection of a pEF1α-FFluc construct.
Lnc^{mut} and *Lnc*^{ORF} in *B* and *D* designate *Lnc473*^{mut} and *Lnc473*^{ORF}, respectively.
Scale bars in *C* are 20 μm (large image) and 5 μm (inset).
Bars in *B* and *D* show means. n = 3 (*B*) or n = 3 - 6 wells from 3 preparations (*D*). ns = not significant. Two-way ANOVAs ($F_{3, 24}$ = 0.04, *B* virus; $F_{2, 24}$ = 32.91, *B* treatment; $F_{3, 40}$ = 0.66, *D* virus; $F_{2, 40}$ = 11.63, *D* treatment) with Dunnett's tests.

Table S13. Electrophysiological properties of mouse hippocampal primary neurons expressing EGFP or *Lnc473* and EGFP (*Lnc473*).

		EGFP	*Lnc473*	D value	*p* value
Membrane capacitance	pF	127.0 ± 38.0 (88)	126.5 ± 48.5 (81)	0.146	0.290
Membrane resistance	MΩ	183.2 ± 131 (88)	175.0 ± 87.1 (81)	0.167	0.164
V_{rest}	mV	-67.0 ± 8.3 (88)	-68.2 ± 9.3 (81)	0.095	0.796
Rheobase	pA	50 ± 50 (69)	47 ± 40 (62)	0.112	0.743
*AP threshold	mV	-42.3 ± 4.9 (67)	-43.5 ± 4.3 (61)	0.237	0.045*
*AP amplitude	mV	67.3 ± 10.8 (68)	71.1 ± 9.3 (61)	0.275	0.013*
#AP half width	ms	1.52 ± 0.75 (68)	1.44 ± 0.74 (61)	0.234	0.051#
AHP amplitude	mV	-14.0 ± 6.2 (68)	-13.4 ± 6.1 (61)	0.147	0.442
AHP peak delay	ms	20.0 ± 24.2 (68)	19.4 ± 19.5 (61)	0.149	0.426
*AP input/output slope	APs/pA	0.106 ± 0.064 (49)	0.130 ± 0.061 (42)	0.323	0.013*
#Accommodation index	AU	1.85 ± 1.02 (68)	1.61 ± 1.22 (60)	0.228	0.059#
Kir conductance	nS	8.05 ± 5.40 (74)	7.86 ± 5.12 (70)	0.135	0.475
HCN conductance	nS	2.00 ± 3.03 (73)	1.94 ± 2.84 (69)	0.119	0.627
Kv4.2 conductance	nS	38.1 ± 28.8 (41)	41.6 ± 29.0 (41)	0.146	0.779
mEPSC IEI	ms	146.4 ± 126.2 (56)	149.2 ± 199.3 (49)	0.173	0.364
mEPSC amplitude	pA	16.77 ± 6.56 (56)	18.83 ± 5. 49 (49)	0.202	0.184

Summary statistics obtained from whole-cell patch-clamp recordings in mouse hippocampal primary cultures expressing EGFP or *Lnc473* and EGFP. Data indicate median ± interquartile range (number of cells) and the D statistic and probability values ($^{\#}p \leq 0.1$, $^{*}p \leq 0.05$) determined from Kolmogorov-Smirnov tests.
Vrest, resting membrane potential; AP, action potential; AHP, afterhyperpolarization potential; HCN, hyperpolarization-activated cyclic nucleotide regulated; Kir, potassium inward rectifier; mEPSC, miniature excitatory postsynaptic current; IEI, inter-event interval; AU, arbitrary units. See Methods section for analyses details.

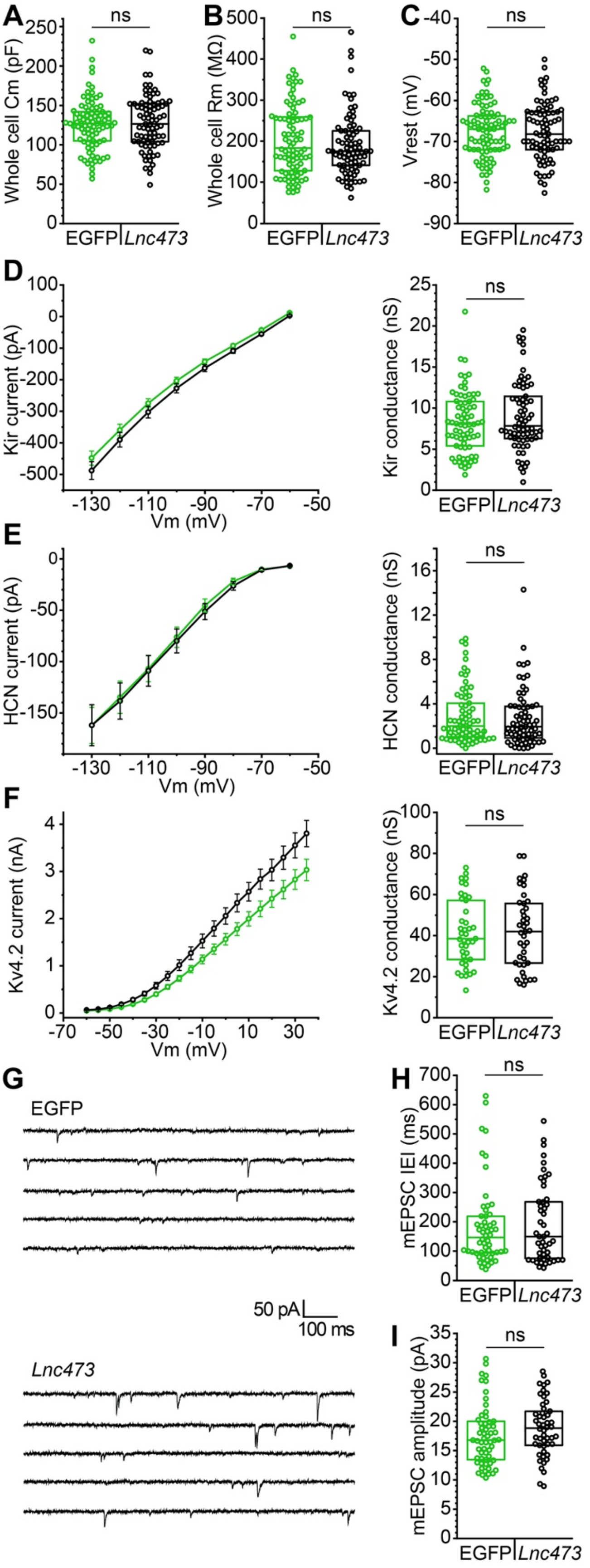

Figure S4. *Lnc473* expression does not affect passive properties, voltage activated conductances or mEPSC frequency and amplitude in mouse hippocampal primary cultures.
Whole cell patch clamp analyses of the effects of *Lnc473* expression in day *in vitro* 10 - 11 cultures of mouse primary neurons infected with an rAAV containing an expression cassette for either EGFP or *Lnc473* and EGFP (*Lnc473*).
A-C, Plots show whole cell membrane capacitance (Cm, *A*); whole cell membrane resistance (Rm, *B*) and resting membrane potential (Vrest, *C*).
D-F, Quantification of K^+ inward rectifier channels (Kir, *D*), hyperpolarization-activated cyclic nucleotide gated channels (HCN, *E*) and transient A-type potassium currents mediated by putative Kv4.2 channels (Kv4.2, *F*). Current voltage relationships on the left show peak current amplitudes. Conductances plotted on the right are the slope values of linear fits of these currents in the range -110 to -130 mV for HCN, -20 to +35 mV for Kv4.2 and as the difference in slope conductance between -70 to -90 mV and -110 to -130 mV for Kir.
G-I, Analysis of mEPSCs. Shown are representative examples of mEPSC recordings from EGFP- and *Lnc473*-expressing cells (*G*) and the quantification of mEPSC inter-event interval (IEI, the inverse of frequency) (*H*) and amplitude (*I*). See Methods section for details of the analyses.
In *A-C*, in *D-F* (right), and in *H* and *I* the values from individual cells (circles), their median (line) and interquartile range (box) are shown. *D-F* (left) show mean ± SEM. For n numbers see Supplementary Table 13. ns, not significant from Kolmogorov-Smirnov tests.

Table S14. Expression of the mouse orthologs of 83 known human epilepsy genes in *Lnc473*-supplemented mouse neurons.

	**Regulation different from chance? (Binominal test)**
**Upregulated 0/83,** i.e., **0%**	**No**, ***p* = 0.7**, chance level 0.422 %<br>95 % CI of observed 0 to 4.42 (%)
**Downregulated 14/83**, i.e., **16.87%**	**Yes**, ***p* < 1e-15,**<br>**ca. 40× more** than chance level of 0.422 %<br>95 % CI of observed 10.32 to 26.34 (%)

Downregulated ortholog	Refs. for epilepsy in human
Cacna1a	Damaj, L. 2015 (84); Reinson, K. 2016 (85)
Dnm1	von Spiczak, S. 2017 (86)
Grin2a	Carvill, G. L. 2013 (120)
Kcnb1	Torkamani, A. 2014 (50)
Kcnc1	Muona, M. 2015 (42)
Kcnma1	Bailey, C. S. 2019 (121)
Kcnq2	Nappi, P. 2020 (51)
Kcnq3	Nappi, P. 2020 (51)
Kcnt1	Bonardi, C. M. 2021 (122)
Pcdh19	Samanta, D. 2020 (87)
Scn1a	Parihar, R. 2013 (123)
Slc12a5	Puskarjov, M. 2014 (124)
Szt2	Basel-Vanagaite, L. 2013 (125)
Tbc1d24	Balestrini, S. 2016 (126)

For this analysis epilepsy genes that when mutated in human cause pure or relatively pure epilepsies, or syndromes with epilepsy as the central symptom (Table 1 from Wang *et al.*, 2017 (127)), were used. *Cacna1h* was removed from the analysis (128).

References

1. Pruunsild, P., Bengtson, C. P., and Bading, H. (2017) Networks of Cultured iPSC-Derived Neurons Reveal the Human Synaptic Activity-Regulated Adaptive Gene Program. *Cell Rep* **18**, 122-135

42. Muona, M., Berkovic, S. F., Dibbens, L. M., Oliver, K. L., Maljevic, S., Bayly, M. A., Joensuu, T., Canafoglia, L., Franceschetti, S., Michelucci, R., Markkinen, S., Heron, S. E., Hildebrand, M. S., Andermann, E., Andermann, F., Gambardella, A., Tinuper, P., Licchetta, L., Scheffer, I. E., Criscuolo, C., Filla, A., Ferlazzo, E., Ahmad, J., Ahmad, A., Baykan, B., Said, E., Topcu, M., Riguzzi, P., King, M. D., Ozkara, C., Andrade, D. M., Engelsen, B. A., Crespel, A., Lindenau, M., Lohmann, E., Saletti, V., Massano, J., Privitera, M., Espay, A. J., Kauffmann, B., Duchowny, M., Moller, R. S., Straussberg, R., Afawi, Z., Ben-Zeev, B., Samocha, K. E., Daly, M. J., Petrou, S., Lerche, H., Palotie, A., and Lehesjoki, A. E. (2015) A recurrent de novo mutation in KCNC1 causes progressive myoclonus epilepsy. *Nat Genet* **47**, 39-46

50. Torkamani, A., Bersell, K., Jorge, B. S., Bjork, R. L., Jr., Friedman, J. R., Bloss, C. S., Cohen, J., Gupta, S., Naidu, S., Vanoye, C. G., George, A. L., Jr., and Kearney, J. A. (2014) De novo KCNB1 mutations in epileptic encephalopathy. *Ann Neurol* **76**, 529-540

51. Nappi, P., Miceli, F., Soldovieri, M. V., Ambrosino, P., Barrese, V., and Taglialatela, M. (2020) Epileptic channelopathies caused by neuronal Kv7 (KCNQ) channel dysfunction. *Pflugers Arch* **472**, 881-898

63. Benito, E., Valor, L. M., Jimenez-Minchan, M., Huber, W., and Barco, A. (2011) cAMP response element-binding protein is a primary hub of activity-driven neuronal gene expression. *J Neurosci* **31**, 18237-18250

84. Damaj, L., Lupien-Meilleur, A., Lortie, A., Riou, E., Ospina, L. H., Gagnon, L., Vanasse, C., and Rossignol, E. (2015) CACNA1A haploinsufficiency causes cognitive impairment, autism and epileptic encephalopathy with mild cerebellar symptoms. *Eur J Hum Genet* **23**, 1505-1512

85. Reinson, K., Oiglane-Shlik, E., Talvik, I., Vaher, U., Ounapuu, A., Ennok, M., Teek, R., Pajusalu, S., Murumets, U., Tomberg, T., Puusepp, S., Piirsoo, A., Reimand, T., and Ounap, K. (2016) Biallelic CACNA1A mutations cause early onset epileptic encephalopathy with progressive cerebral, cerebellar, and optic nerve atrophy. *Am J Med Genet A* **170**, 2173-2176

86. von Spiczak, S., Helbig, K. L., Shinde, D. N., Huether, R., Pendziwiat, M., Lourenco, C., Nunes, M. E., Sarco, D. P., Kaplan, R. A., Dlugos, D. J., Kirsch, H., Slavotinek, A., Cilio, M. R., Cervenka, M. C., Cohen, J. S., McClellan, R., Fatemi, A., Yuen, A., Sagawa, Y., Littlejohn, R., McLean, S. D., Hernandez-Hernandez, L., Maher, B., Moller, R. S., Palmer, E., Lawson, J. A., Campbell, C. A., Joshi, C. N., Kolbe, D. L., Hollingsworth, G., Neubauer, B. A., Muhle, H., Stephani, U., Scheffer, I. E., Pena, S. D. J., Sisodiya, S. M., Helbig, I., Epi, K. C., and Euro, E.-R. E. S. N. W. G. (2017) DNM1 encephalopathy: A new disease of vesicle fission. *Neurology* **89**, 385-394

87. Samanta, D. (2020) PCDH19-Related Epilepsy Syndrome: A Comprehensive Clinical Review. *Pediatr Neurol* **105**, 3-9

103. Anders, S., Reyes, A., and Huber, W. (2012) Detecting differential usage of exons from RNA-seq data. *Genome Res* **22**, 2008-2017

106. Love, M. I., Huber, W., and Anders, S. (2014) Moderated estimation of fold change and dispersion for RNA-seq data with DESeq2. *Genome Biol* **15**, 550

116. Impey, S., McCorkle, S. R., Cha-Molstad, H., Dwyer, J. M., Yochum, G. S., Boss, J. M., McWeeney, S., Dunn, J. J., Mandel, G., and Goodman, R. H. (2004) Defining the CREB regulon: a genome-wide analysis of transcription factor regulatory regions. *Cell* **119**, 1041-1054

117. Zhang, X., Odom, D. T., Koo, S. H., Conkright, M. D., Canettieri, G., Best, J., Chen, H., Jenner, R., Herbolsheimer, E., Jacobsen, E., Kadam, S., Ecker, J. R., Emerson, B., Hogenesch, J. B., Unterman, T., Young, R. A., and Montminy, M. (2005) Genome-wide analysis of cAMP-response element binding protein occupancy, phosphorylation, and target gene activation in human tissues. *Proc Natl Acad Sci U S A* **102**, 4459-4464

118. Xu, W., Kasper, L. H., Lerach, S., Jeevan, T., and Brindle, P. K. (2007) Individual CREB-target genes dictate usage of distinct cAMP-responsive coactivation mechanisms. *EMBO J* **26**, 2890-2903
119. Pardo, L., Valor, L. M., Eraso-Pichot, A., Barco, A., Golbano, A., Hardingham, G. E., Masgrau, R., and Galea, E. (2017) CREB Regulates Distinct Adaptive Transcriptional Programs in Astrocytes and Neurons. *Sci Rep* **7**, 6390
120. Carvill, G. L., Regan, B. M., Yendle, S. C., O'Roak, B. J., Lozovaya, N., Bruneau, N., Burnashev, N., Khan, A., Cook, J., Geraghty, E., Sadleir, L. G., Turner, S. J., Tsai, M. H., Webster, R., Ouvrier, R., Damiano, J. A., Berkovic, S. F., Shendure, J., Hildebrand, M. S., Szepetowski, P., Scheffer, I. E., and Mefford, H. C. (2013) GRIN2A mutations cause epilepsy-aphasia spectrum disorders. *Nat Genet* **45**, 1073-1076
121. Bailey, C. S., Moldenhauer, H. J., Park, S. M., Keros, S., and Meredith, A. L. (2019) KCNMA1-linked channelopathy. *J Gen Physiol* **151**, 1173-1189
122. Bonardi, C. M., Heyne, H. O., Fiannacca, M., Fitzgerald, M. P., Gardella, E., Gunning, B., Olofsson, K., Lesca, G., Verbeek, N., Stamberger, H., Striano, P., Zara, F., Mancardi, M. M., Nava, C., Syrbe, S., Buono, S., Baulac, S., Coppola, A., Weckhuysen, S., Schoonjans, A. S., Ceulemans, B., Sarret, C., Baumgartner, T., Muhle, H., des Portes, V., Toulouse, J., Nougues, M. C., Rossi, M., Demarquay, G., Ville, D., Hirsch, E., Maurey, H., Willems, M., de Bellescize, J., Altuzarra, C. D., Villeneuve, N., Bartolomei, F., Picard, F., Hornemann, F., Koolen, D. A., Kroes, H. Y., Reale, C., Fenger, C. D., Tan, W. H., Dibbens, L., Bearden, D. R., Moller, R. S., and Rubboli, G. (2021) KCNT1-related epilepsies and epileptic encephalopathies: phenotypic and mutational spectrum. *Brain*
123. Parihar, R., and Ganesh, S. (2013) The SCN1A gene variants and epileptic encephalopathies. *J Hum Genet* **58**, 573-580
124. Puskarjov, M., Seja, P., Heron, S. E., Williams, T. C., Ahmad, F., Iona, X., Oliver, K. L., Grinton, B. E., Vutskits, L., Scheffer, I. E., Petrou, S., Blaesse, P., Dibbens, L. M., Berkovic, S. F., and Kaila, K. (2014) A variant of KCC2 from patients with febrile seizures impairs neuronal Cl- extrusion and dendritic spine formation. *EMBO Rep* **15**, 723-729
125. Basel-Vanagaite, L., Hershkovitz, T., Heyman, E., Raspall-Chaure, M., Kakar, N., Smirin-Yosef, P., Vila-Pueyo, M., Kornreich, L., Thiele, H., Bode, H., Lagovsky, I., Dahary, D., Haviv, A., Hubshman, M. W., Pasmanik-Chor, M., Nurnberg, P., Gothelf, D., Kubisch, C., Shohat, M., Macaya, A., and Borck, G. (2013) Biallelic SZT2 mutations cause infantile encephalopathy with epilepsy and dysmorphic corpus callosum. *Am J Hum Genet* **93**, 524-529
126. Balestrini, S., Milh, M., Castiglioni, C., Luthy, K., Finelli, M. J., Verstreken, P., Cardon, A., Strazisar, B. G., Holder, J. L., Jr., Lesca, G., Mancardi, M. M., Poulat, A. L., Repetto, G. M., Banka, S., Bilo, L., Birkeland, L. E., Bosch, F., Brockmann, K., Cross, J. H., Doummar, D., Felix, T. M., Giuliano, F., Hori, M., Huning, I., Kayserili, H., Kini, U., Lees, M. M., Meenakshi, G., Mewasingh, L., Pagnamenta, A. T., Peluso, S., Mey, A., Rice, G. M., Rosenfeld, J. A., Taylor, J. C., Troester, M. M., Stanley, C. M., Ville, D., Walkiewicz, M., Falace, A., Fassio, A., Lemke, J. R., Biskup, S., Tardif, J., Ajeawung, N. F., Tolun, A., Corbett, M., Gecz, J., Afawi, Z., Howell, K. B., Oliver, K. L., Berkovic, S. F., Scheffer, I. E., de Falco, F. A., Oliver, P. L., Striano, P., Zara, F., Campeau, P. M., and Sisodiya, S. M. (2016) TBC1D24 genotype-phenotype correlation: Epilepsies and other neurologic features. *Neurology* **87**, 77-85
127. Wang, J., Lin, Z. J., Liu, L., Xu, H. Q., Shi, Y. W., Yi, Y. H., He, N., and Liao, W. P. (2017) Epilepsy-associated genes. *Seizure* **44**, 11-20
128. Calhoun, J. D., Huffman, A. M., Bellinski, I., Kinsley, L., Bachman, E., Gerard, E., Kearney, J. A., and Carvill, G. L. (2020) CACNA1H variants are not a cause of monogenic epilepsy. *Hum Mutat* **41**, 1138-1144
